# Pharmacokinetic and safety profiles of mesalazine enema in healthy Chinese subjects: A single- and multiple-dose study

**DOI:** 10.1371/journal.pone.0296940

**Published:** 2024-02-02

**Authors:** Yuran Cao, Jingjing Wang, Xingyu Tang, Yan Tian, Jicheng Yu, Hong Liang, Jufang Wu, Yuancheng Chen, Guoying Cao, Jing Zhang

**Affiliations:** 1 Clinical Trial Institute, Huashan Hospital, Fudan University, Shanghai, China; 2 Phase I Clinical Research Center, Huashan Hospital, Fudan University, Shanghai, China; University of Milan, ITALY

## Abstract

Mesalazine is a well-established treatment for ulcerative colitis by oral or topical administration. However, the pharmacokinetic (PK) and safety profiles of mesalazine administered by an enema has not been clarified in Chinese population. We conducted an open-label study to assess the PK and safety profiles of mesalazine in 11 healthy Chinese subjects after receiving mesalazine enema (1 g/100 mL) once daily for 7 consecutive days. Blood and urine samples were collected for assay of mesalazine and N-acetyl mesalazine by liquid chromatography-tandem mass spectrometry. The PK and safety data were summarized using descriptive statistics. The mean (standard deviation) maximum plasma concentration (C_max_), area under plasma drug concentration-time curve from time 0 to the last measurable plasma concentration time point (AUC_0-_t) and elimination half-life (t_1/2_) of mesalazine were 1007.64 (369.00) ng/mL, 9608.59 (3533.08) h·ng/mL and 3.33 (1.99) h, respectively after the first dose administration. In multiple-dose study, the estimated accumulation factor of mesalazine was 1.09. The cumulative urinary excretion rate of parent and major metabolite of mesalazine was 27.77%. After the last doe administration, 2.21% of the administered dose was excreted as mesalazine and 24.47% as N-acetyl mesalazine in urine within 24 h. Overall, 9 adverse events (AEs) were reported in 4 of the 11 subjects (36.4%), including oral ulcer, toothache, upper respiratory tract infection (1 each) and laboratory abnormalities (6 cases). All AEs were mild and recovered spontaneously without treatment, and were not considered as related to mesalazine. Mesalazine enema (1 g/100 mL) was safe and well tolerated in healthy Chinese subjects. These findings support further clinical trials in Chinese patients.

**Trial registration:** This trial was registered to Chinese Clinical Trial Registry (ChiCTR) at https://www.chictr.org.cn (registration number: ChiCTR2300073148).

## Introduction

Ulcerative colitis (UC) is a chronic idiopathic inflammatory disease of the colon and rectum, manifested as persistent or recurrent diarrhea, mucous purulent blood stool, and abdominal pain, and characterized by recurrent disease flares and periods of remission (quiescence). The disease can be recognized as proctitis, left-sided colitis, extensive colitis, or pancolitis. The long-lasting symptoms can cause significant impairment of quality of life. UC is considered a progressive disease due to the risks of proximal extension, colectomy, and colorectal cancer [1]. The annual incidence of UC is 1.18 per 100,000 person-years in China. About 69.8% of the Chinese UC patients had moderate-to-severe disease [2].

Mesalazine (5-aminosalicylate) is a well-established treatment for UC administered orally (tablets, capsules or sachets) or topically (liquid or foam suspensions, gels, suppositories). Currently, it is the standard of care for induction and maintenance of remission in patients with mild-to-moderate disease [3–8]. It is reported that mesalazine exerts extensive anti-inflammatory effects by inhibition of leukotriene and IL-1 production, impairment of TNFα and NF-κB transcription signaling, and acting as a free-radical scavenger [9, 10]. Mesalazine therefore can reduce mucosal inflammation by acting on colonic epithelial cells and infiltrating leukocytes. The clinical efficacy of mesalazine is correlated with its local concentration in the gut [11]. In some cases, mesalamine concentration is insufficient in the inflamed distal colon after oral administration. For this reason, topical therapy with mesalazine enema (1g/d) is recommend by practice guidelines as the preferred option to maintain remission in distal mild-to-moderate UC, particularly in proctitis and left-sided colitis [3, 7]. Topical therapy can provide some advantages such as quicker response to treatment, lower dosing frequency, and less systemic absorption compared to oral therapy.

Mesalazine enema is available with different strengths, including 1 g/100 mL, 2 g/30 mL, and 4 g/60 mL. The clinical pharmacokinetic (PK) studies of mesalazine enema in foreign populations [12–14] demonstrated that for patients with mild to moderate active UC, 1 g mesalazine enema was an adequate therapeutic dose [13]. In contrast to 2 g/30 mL and 4 g/60 mL enemas, 1 g/100 ml mesalazine enema provided higher local bioavailability and wider distribution in the descending colon due to its larger volume [14]. However, 1 g/100 mL mesalazine enema is not available in China. The safety and systemic exposure of 1 g/100 mL mesalazine enema after rectal administration should be clarified in Chinese population before it can be safely and effectively used in Chinese patients.

## Methods

The study was performed at the Phase I Clinical Research Center, Huashan Hospital, Fudan University (Shanghai, China), after approval by the local ethics committee and the National Medical Products Administration (NMPA). The study was conducted in compliance with the protocol, the principles of the Declaration of Helsinki, Good Clinical Practice and applicable local regulations. Written informed consent was obtained from all participants before study. The trial was previously registered at Chinese Clinical Trial Registrysponsored by the Center for Drug Evaluation of NMPA (registration number: CTR20181364). Unfortunately, the registry is not in the World Health Organization list of approved registries. For the purpose of international access to the information of this trial, we also retrospectively registered this trial to Chinese Clinical Trial Registry (ChiCTR) at https://www.chictr.org.cn with an identifier ChiCTR2300073148. We confirmed that all ongoing and related trials for this intervention are registered.

### Study design

This was an open-label, single- and multiple-dose study in healthy Chinese subjects. Subjects were screened from day -14 to day -1 before the first administration of investigational medical product. It was expected that 10 eligible subjects were admitted to the study site 1 day before treatment, received mesalazine enema (1 g/100 mL, batch number: K15446A, supplied by Ferring Pharmaceuticals A/S, Copenhagen, Denmark) at 9:00–10:00 pm, once daily from days 1–7. The enema was dispensed and administered by a trial nurse, and retained for at least 8 hours after administration. Subjects were fasted and deprived of water from 2 hours before administration until 8 hours post administration. Approximately 2 hours before each administration, subjects were required to empty their intestines as much as possible. Warm water enema was used to empty the intestine before the first and last doses, but not before the second to sixth doses. In addition, subjects were required to empty their bladder approximately 10 minutes before each administration. The subjects were discharged after the end-of-trial examination on day 9.

The primary study objectives were to determine the PK parameters of mesalazine after rectal administration of single and multiple doses of mesalazine enema in healthy Chinese subjects. The secondary objectives were to assess the safety and total cumulative amount of mesalazine and N-acetyl-mesalazine excreted in urine after rectal administration of single and multiple doses of the enema.

### Subjects

The inclusion criteria included: healthy Chinese males or females (non-pregnant and non-lactating), 18 to 45 years of age, body weight ≥ 50 kg for males or ≥ 45 kg for females and a body mass index (BMI) of 19–24 kg/m^2^; in good general health based on a thorough health checkup and screening tests; and bowel movement frequency from once a day to three times a week within 3 months before this study. Female subjects have a menstrual cycle of 28–35 days and a menstrual period of 3–7 days within 6 months prior to screening, with the end of menstruation within 7 days prior to day -1 of the study. From day -14 to day -2, subjects were able to retain a dose of placebo enema for at least 30 minutes and the weight of the diaper increased no more than 20 g; On day -1, subjects were able to retain a dose of placebo enema for at least 8 hours and did not gain more than 20 g of diaper weight. Placebo enema (100 mL) was identical to the mesalazine enema in appearance, but did not contain mesalazine. The compliance of treatment was evaluated using the weight gain of a diaper.

Subjects were excluded if they had any of the following conditions: allergy to any drug, history of drug abuse, smoking or alcoholism before the study; presence or history of clinically significant renal, hepatic, gastrointestinal, cardiovascular, musculoskeletal, psychiatric, immune, endocrine, or metabolic disease; had cancer in the past 5 years, except for adequately treated basal cell carcinoma and squamous cell carcinoma of the skin; prior gastrointestinal surgery; history of anorectal disease, including fistulas, structural abnormalities, and rectal pain that interfere with the retention of bowel fluid; blood donation or loss of more than 500 mL within 12 weeks prior to administration; consumption of large amounts of caffeinated beverages per day prior to the study; participation in another clinical study within 12 weeks prior to the study; disagreed to remain abstinent from the time they signed the informed consent until 3 days after the end-of-trial examination. No deviation from the clinical protocol was found during the entire study. The authors were not blinded to the information that could identify individual participants during data collection due to the open-label nature of study design.

### Collection and analysis of blood and urine samples

Blood samples were collected immediately before administration and 0.5, 1, 2, 3, 4, 5, 6, 7, 8, 10, 12, 14, 16, 20 and 24 h after administration for the first, 5^th^ and 7^th^ dose of mesalazine enema. Approximately 4 mL of blood were collected at each time point. Urine samples were collected for analysis of mesalazine and its major metabolite N-acetyl mesalazine. Blank urine (5–10 mL) were collected prior to administration of the first dose, and all urine samples were collected within 0–12 h and 12–24 h after administration of the 7^th^ dose.

The concentrations of mesalazine in human plasma and mesalazine and N-acetyl mesalazine in human urine were detected by WuXi AppTec Co., Ltd (Shanghai, China). Two liquid chromatographic- tandem mass spectrometric (LC-MS/MS) methods were developed and validated for quantification of mesalazine and N-acetyl mesalazine according to current regulatory standards for bioanalysis. In brief, 100 μL plasma was placed into a 96-well plate and mixed with 20 μL internal standard solution and 280 μL acetonitrile. The mixture was vortexed for 10 min and then centrifuged for 10 min at 4000 rpm. The upper organic layer was transferred into another 96-well plate, and mixed with 50 μl mobile phase. Finally, 5 μL aliquot was injected into the LC-MS/MS system. For urine samples, 50 μL aliquot was placed into a 96-well plate and mixed with 250 μL internal standard solution, and vortexed for at least 5 min. The upper organic layer was transferred into another 96-well plate, mixed with 450 μl mobile phase, and then vortexed for at least 5 min. Finally, 10 μL aliquot was injected into the LC-MS/MS system.

The LC-MS/MS analysis of mesalazine in human plasma was carried out with an Applied Biosystems / Sciex API 4000 mass spectrometer coupled with an Agilent 1200 series LC liquid phase system. Chromatographic separation was achieved on an Atlantis HILIC Silica 3 µm 2.1 x 100 mm (from Waters) column with a mobile phase gradient. The mass spectrometer was operated in negative electrospray ionization mode and selected multiple response monitoring model. Selected reaction monitoring transitions were m/z 152.0→108.0 for mesalazine. The LC-MS/MS assay of mesalazine and N-acetyl mesalazine in human urine was carried out with an Applied Biosystems / Sciex API 4000 mass spectrometer coupled with an Shimadzu LC-20AD LC liquid phase system. Chromatographic separation was achieved on a Gemini C18 3 µm 2.0 x 150 mm (Phenomenex) column with a mobile phase gradient. Selected reaction monitoring transition was m/z 152.0→107.0 for mesalazine and m/z 194.0→150.0 for N-acetyl mesalazine.

The LC-MS/MS methods were fully validated in terms of specificity, matrix effect, recovery, carryover, accuracy, precision, dilutions and stability under different conditions. The lower limit of quantitation (LLOQ) of mesalazine was 2.00 ng/mL (range from 2.00 to 1200 ng/mL) in human plasma. The LLOQ of mesalazine and N-acetyl mesalazine was 200 ng/mL (range: 200 to 100000 ng/mL) and 1000 ng/mL (range: 1000 to 500000 ng/mL) in human urine, respectively. No significant matrix effects were observed. The relative standard deviations of inter-day and intra-day accuracy and precision were within 20% of LLOQ level and within 15% of the other quality control (QC) levels. The coefficient of variation was less than 20% and 15% of the LLOQ level and the other QC levels, respectively. The average recovery of mesalazine was 89.0% from plasma and 88.8% from urine. The average recovery of N-acetyl mesalazine was 100.9% from urine. The analytes were stable in human plasma after five freeze-thaw cycles, for 24.8 hours at room temperature and up to 61 days at—70°C or lower. The analytes were stable in human urine after four freeze-thaw cycles, for 29 hours at room temperature and for up to 42 days at -20°C and -80°C.

### PK assessment

The plasma PK parameters of mesalazine were calculated by non-compartmental model using Phoenix software (WinNonlin version 6.4; Pharsight Corp, Cary, NC, USA). Actual sampling time points after administration were used for non-compartmental analysis and individual plasma concentration-time curves. When a plasma concentration was lower than the LLOQ, it was calculated as 0 if the sample was taken before reaching the maximum plasma concentration (C_max_), or as not detectable (ND) if the sample was taken after reaching the C_max_. PK parameters were estimated based on the measurements on days 1–2 (the first dose) in the single-dose part, days 5–6 (the 5^th^ dose) and days 7–8 (the 7^th^ dose) in the multiple-dose part, including C_max_, time to C_max_ (T_max_), area under plasma drug concentration-time curve (AUC, which was calculated by linear trapezoidal method) from time 0 to the last measurable plasma concentration time point (AUC_0-t_), elimination half-life (t_1/2_), apparent volume of distribution (V_z_/F), apparent clearance (CL/F), and average plasma concentration in the multiple dose study (C_avg_). In addition, the accumulation factor (R) and its 90% confidence interval (CI) were used to evaluate the potential accumulation of mesalazine after multiple-dose administration, which were calculated by comparing the AUC after the first dose and the AUC from time zero up to the end of the dosing period (time tau, 24 h after last dose) (AUC_tau_) after the 7^th^ dose. Urine PK parameters were also calculated, including the cumulative urinary excretion amount (A_e_) and the cumulative urinary excretion rate (F_e_) of mesalazine and N-acetyl mesalazine. A_e_ of N-acetyl-mesalazine was calculated as cumulative urinary excretion amount /195.17 (molecular weight of N-acetyl-mesalazine) × 153.14 (molecular weight of mesalazine).

### Safety assessment

Vital signs, physical examination, electrocardiogram, safety laboratory variables, and adverse events (AEs) were monitored throughout the study. Laboratory measures included hematology, clinical chemistry, urinalysis, and serum pregnancy test (women of childbearing potential only). The subjects were instructed to notify the study physicians or nurses of any AEs that occurred during the study. All AEs reported by subjects or detected in the assessments were recorded. The investigators assessed the relatedness of AEs to the study drug.

### Statistical analysis

The sample size was determined per the NMPA guidance on clinical PK study. No formal sample size calculations were performed for this trial. Missing values were not imputed. Descriptive statistical analysis was performed for PK parameters using SAS 9.2 statistical software (SAS Institute Inc., Cary, NC, USA). The accumulation factor R [AUC_tau_ (dose 7)/AUC (dose 1)] and the corresponding 90% CI were calculated using a mixed-effects model of the log-transformed exposure parameter (AUC), and day was fitted as a fixed effect in the model. Descriptive statistics were used for the safety analysis set, including all subjects who received at least one dose of the study drug and have post-administration safety evaluation. AEs were coded using the Medical Dictionary for Regulatory Activities (version 18.0). AEs were classified as either pre-treatment emergent or treatment-emergent (TEAE). Only TEAEs were presented in summary tables. No formal statistical comparisons or interim analysis were conducted for this study.

## Results

### Subject demographics

**Fig 1:**
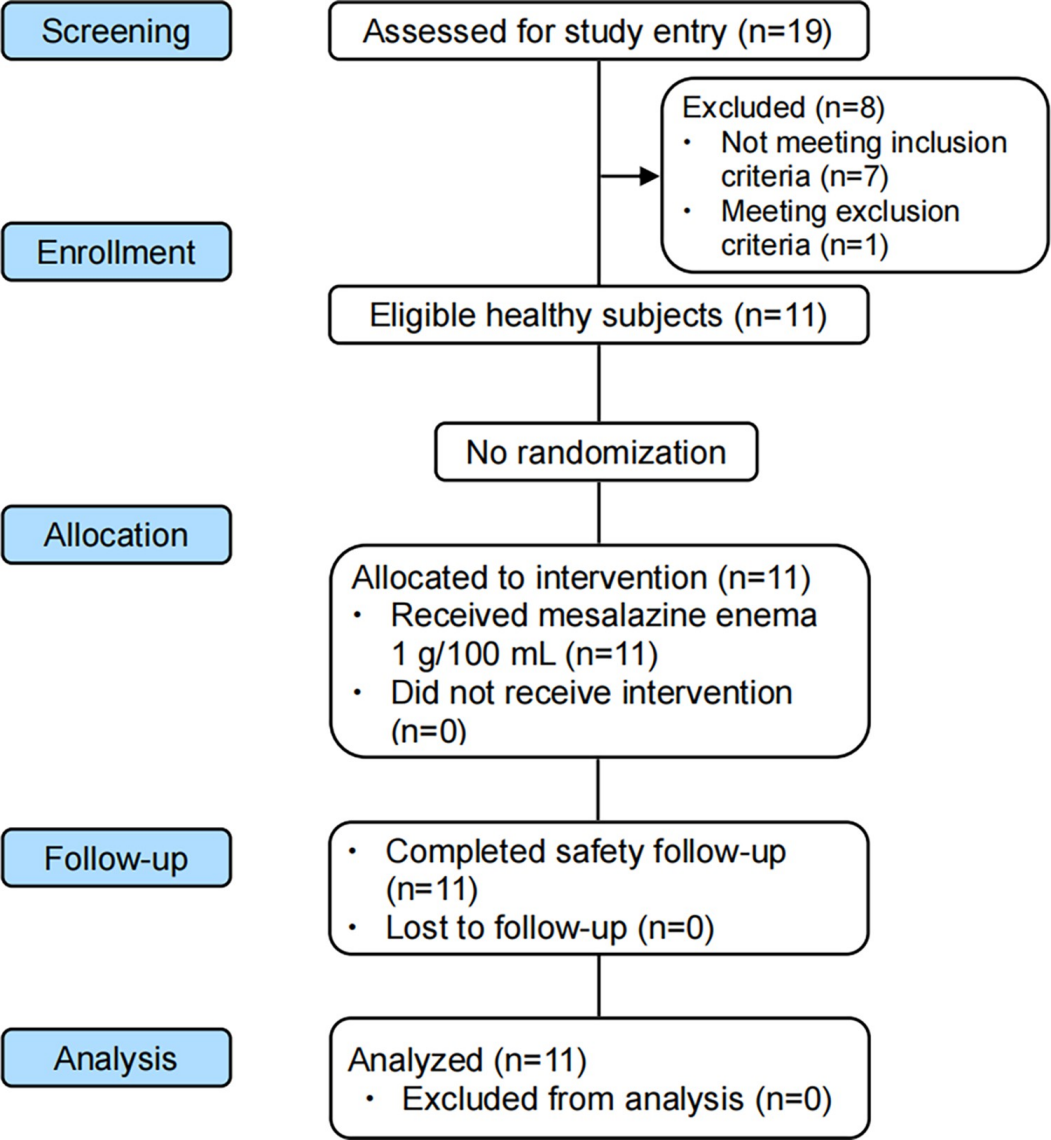
CONSORT flowchart showing the disposition of subjects.

**Fig 2:**
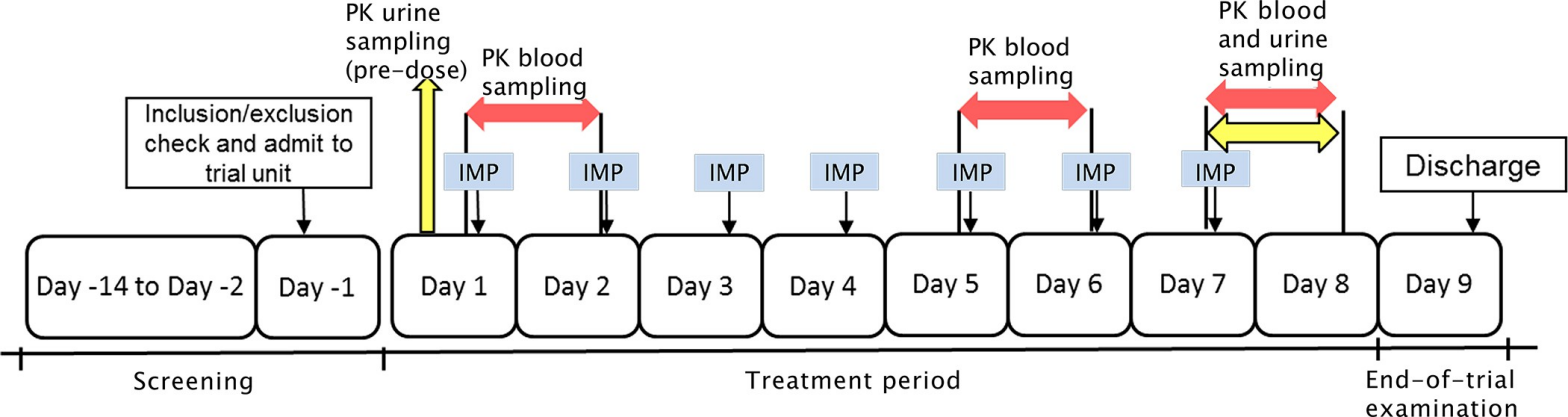
Study procedures. There is no Day 0 in the study. IMP, investigational medical product; PK, pharmacokinetic.

Of the 19 subjects screened, 11 were successfully enrolled in the study, including 7 males (63.64%) and 4 females (36.36%). The mean (standard deviation, SD) age of the subjects was 27.65 (2.68) years old, and mean (SD) BMI was 21.84 (1.41) kg/m^2^. The subjects were recruited during the period from July 28, 2015 to August 27, 2015. They were treated with the study drugs since August 27, 2015, and followed up until the end of the study on September 11, 2015. All subjects completed the study according to the study procedures (Figs 1 and 2).

### Pharmacokinetic results

**Fig 3:**
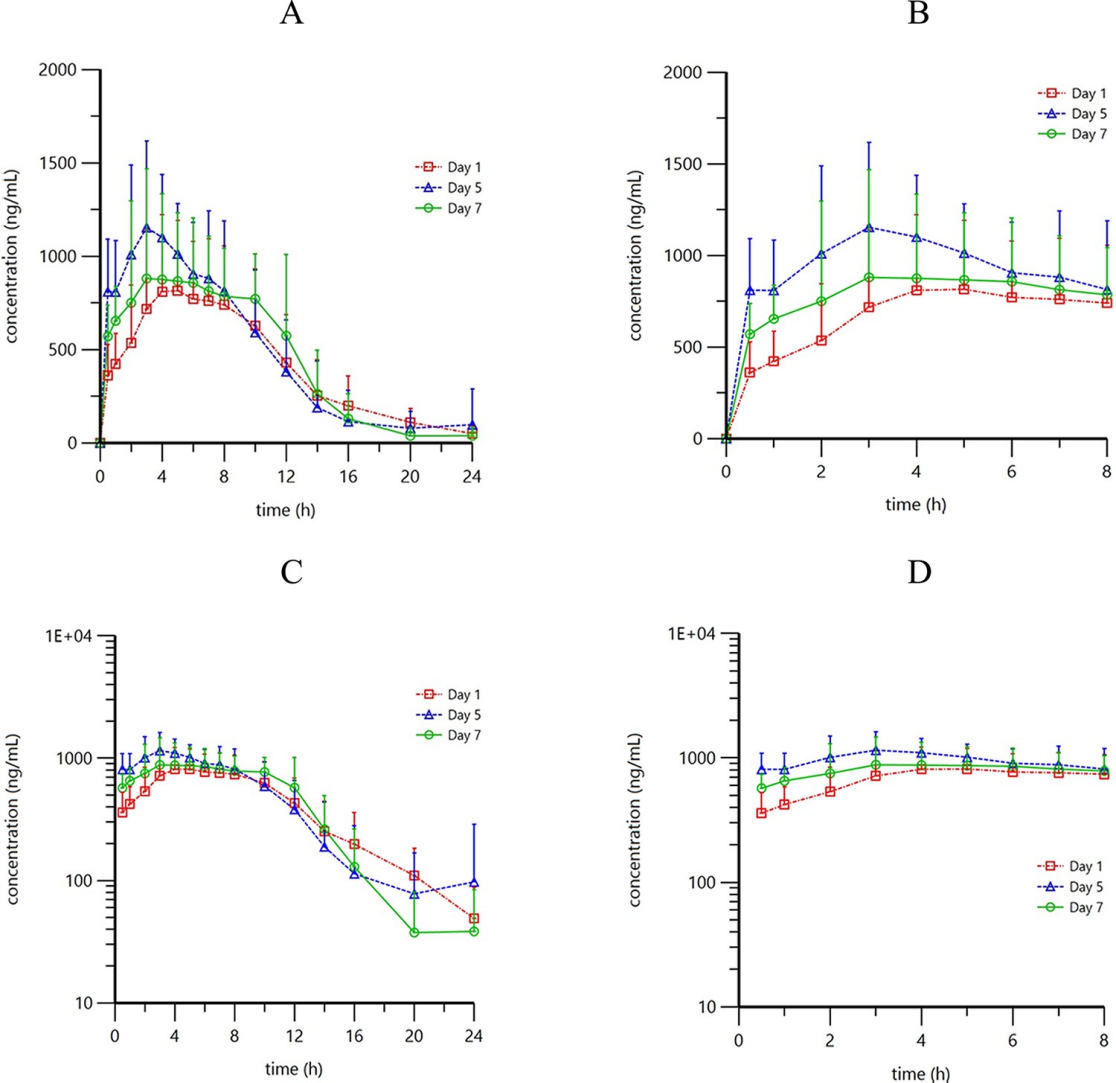
Mean plasma mesalazine concentration-time profiles (linear and semi-logarithmic) after the first, 5^th^ and 7^th^ dose administration of 1 g/100 mL mesalazine enema in healthy Chinese subjects. (A) Linear concentration plot; (B) Zoom in view of panel A from 0 to 8 hours; (C) Semi-logarithmic concentration plot; (D) Zoom in view of panel C from 0 to 8 hours. Each plot represents the mean (standard deviation) concentration of mesalazine.

The mean plasma concentration-time profiles of mesalazine after the first, 5^th^ and 7^th^ dose administration in healthy Chinese subjects are presented in Fig 3. Following a single dose administration of 1 g/100 mL mesalazine enema, the mean (SD) T_max_, C_max_, AUC_0-t_, and t_1/2_ were 7.01 (2.90) h, 1007.64 (369.00) ng/mL, 9608.59 (3533.08) h·ng/mL, and 3.33 (1.99) h, respectively. After administration of 1 g/100 mL mesalazine enema once daily for 5 days, the mean (SD) T_max_, C_max_, AUC_tau_, and t_1/2_ were 4.47 (2.16) h, 1318.82 (455.76) ng/mL, 11196.88 (3370.60) h·ng/mL, and 2.59 (2.32) h. After 7-day treatment, the corresponding values were 6.46 (3.45) h, 1122.73 (551.92) ng/mL, 10705.86 (3868.87) h·ng/mL, and 2.07 (1.63) h, respectively (Table 1). The estimated R and its 90% CI were 1.09 (0.80, 1.47) after administration of 1 g/100 mL mesalazine enema for consecutive 7 days.

**Table 1:** Pharmacokinetic parameters of mesalazine after the first, 5^th^ and 7^th^ dose of 1 g/100 mL mesalazine enema in healthy Chinese subjects.

PK parameter	Statistic	After 1^st^ dose	After 5^th^ dose	After 7^th^ dose
		N = 11	N = 11	N = 11
C_max_ (ng/mL)	Mean (SD)	1007.64 (369.00)	1318.82 (455.76)	1122.73 (551.92)
	Min, Max	425.00, 1530.00	769.00, 2180.00	370.00, 2230.00
T_max_ (h)	Mean (SD)	7.01 (2.90)	4.47 (2.16)	6.46 (3.45)
	Min, Max	3.02, 12.02	2.02, 8.02	1.00, 12.00
t_1/2_ (h)	Mean (SD)	3.33 (1.99)	2.59 (2.32)	2.07 (1.63)
	Min, Max	0.84, 6.87	0.73, 8.33	0.59, 5.53
AUC_0-t_ (h·ng/mL)	Mean (SD)	9608.59 (3533.08)	11196.87 (3370.60)	10705.86 (3868.87)
	Min, Max	3752.00, 16353.05	6440.04, 16762.98	4057.33, 17247.26
C_avg_ (ng/mL)	Mean (SD)	NA	466.54 (140.44)	446.08 (161.20)
	Min, Max		268.33, 698.46	169.06, 718.64
CL/F (L/h)	Mean (SD)	118.49 (57.24)	NA	NA
	Min, Max	60.46, 265.53	NA	NA
V_z_/F (L)	Mean (SD)	478.78 (210.21)	NA	NA
	Min, Max	130.10, 762.37	NA	NA

C_max_, maximum plasma concentration; T_max_, time to the maximum plasma concentration; t_1/2_, elimination half-life; AUC_0-t_, area under plasma drug concentration-time curve from time 0 to the last measurable plasma concentration time point; C_avg_, average plasma concentration in multiple dose period; CL/F, apparent clearance; V_z_/F, apparent volume of distribution; SD, standard deviation; Min, minimum; Max, maximum; N, number of subject; NA, not available.

After the last dose administration of 1 g/100 mL mesalazine enema, 2.21% of the administered dose was excreted as mesalazine and 24.47% as N-acetyl mesalazine within 24 h. Based on the urinary excretion data, the cumulative urinary excretion rate of parent and major metabolite of mesalazine enema was approximately 27.77% after administration once daily for 7 days in healthy Chinese subjects (Table 2).

**Table 2:** Urinary excretion parameters of mesalazine and N-acetyl mesalazine within 24 h after the 7^th^ dose administration of 1 g/100 mL mesalazine enema in healthy Chinese subjects.

Analyte	Parameter	Statistic	Result
Mesalazine	Ae (g)	N (ND)	9 (2)
		Mean (SD)	0.022 (0.016)
		Min, Max	0.00, 0.050
	Fe (%)	N (ND)	9 (2)
		Mean (SD)	2.21 (1.62)
		Min, Max	0.40, 4.50
N-acetyl mesalazine	Ae (g)	N (ND)	11 (0)
		Mean (SD)	0.24 (0.10)
		Min, Max	0.11, 0.41
	Fe (%)	N (ND)	11 (0)
		Mean (SD)	24.47 (9.91)
		Min, Max	10.90, 40.80
Mesalazine + N-acetyl mesalazine	Ae (g)	N (ND)	9 (2)
		Mean (SD)	0.28 (0.12)
		Min, Max	0.12, 0.45
	Fe (%)	N (ND)	9 (2)
		Mean (SD)	27.77 (11.61)
		Min, Max	12.40, 45.10

A_e_, cumulative urinary excretion amount; F_e_, cumulative urinary excretion rate; N, number of subjects; N, number of subjects; ND, number of subjects with mesalazine concentrations not detectable; SD, standard deviation; Min, minimum; Max, maximum.

### Safety

Mesalazine enema (1 g/100 mL) was well-tolerated in all subjects. No deaths or serious adverse events (SAEs) were reported. No subjects discontinued the study due to AEs. Overall, 9 AEs were reported in 4 of the 11 subjects (36.4%), including oral ulcer, toothache, upper respiratory tract infection (1 each) and laboratory abnormalities (6 cases) (Table 3). All AEs were mild and recovered spontaneously without treatment, and were not considered as related to mesalazine. The initial onset of AE was on day 2 (before the second dose) for 4 cases and on days 3–9 for 5 cases. AE incidence was not correlated with the increasing doses of mesalazine enema.

**Table 3:** Summary of adverse events reported in 11 healthy Chinese subjects.

Adverse event	Incidence in 11 subjects	
	Number	%
Total	4	36.4
Gastrointestinal disorder	2	18.2
Oral ulcer	1	9.1
Toothache	1	9.1
Infections and infestations	1	9.1
Upper respiratory tract infection	1	9.1
Laboratory abnormalities	2	18.2
Increased white blood cell count in urine	1	9.1
Positive leukocyte esterase test	1	9.1
Urinary cast	1	9.1
Bacterial count in urine increased	1	9.1
Occult blood in urine	1	9.1
Red blood cells increased in urine	1	9.1

## Discussion

According to the NMPA guideline for clinical PK studies [15], 8 subjects were considered enough to provide sufficient information on the PK profile of mesalazine enema. Assuming a dropout rate of 20%, 10 eligible subjects will be enrolled for this study. However, in clinical practice, many patients do not tolerate mesalazine liquid enemas well or have difficulties in retaining such enemas [16]. In order to ensure sufficient data available for analysis, 19 subjects were screened and finally 11 subjects were enrolled in this study for the possibility of subject withdrawal.

The plasma exposure parameters (C_max_ and AUC_0-t_) of mesalazine after multiple-dose administration (1318.82 ± 455.76 ng/mL and 11196.88 ± 3370.60 h·ng/mL after 5 doses; 1122.73 ± 551.92 ng/mL and 10705.86 ± 3868.87 h·ng/mL after 7 doses) did not show significant difference from the results after a single-dose administration of 1 g/100 mL mesalazine enema (1007.64 ± 369.00 ng/mL and 9608.59 ± 3533.08 h·ng/mL). Furthermore, the systemic accumulation of mesalazine was minimal after rectal administration for consecutive 7 days (R = 1.09). Therefore, multiple-dose administration of 1 g/100 mL mesalazine enema did not significantly increase mesalazine exposure in human plasma. Mesalazine is metabolized to inactive metabolite N-acetyl mesalazine in the small intestinal mucosa (prior to systemic absorption) and in the liver (systemic absorption) [17, 18]. Both substances were considered to be excreted in the urine and feces. In the first 24 h after the last dose administration of 1 g/100 mL mesalazine enema, 2.21% of the administered dose was excreted as mesalazine and 24.47% as N-acetyl mesalazine. These findings were similar to previous report [19].

Systemic drug accumulation after multiple-dose administration was evaluated by comparing the AUC after the first dose with that after the last dose. A warm water enema was used to empty the intestine before the first and last dose administration in order to exclude potential effects of intestinal contents on absorption of mesalazine. This procedure is not exactly the same as the operation in clinical practice. Therefore, the real PK profiles in clinical use could not be fully reflected by the PK parameters after the first and last dose due to the presence of intestinal contents. Warm water enema was not used before the 5^th^ dose administration, the corresponding data were appropriate for evaluating the PK of mesalazine in real-world healthcare.

Mesalazine enema was administered as a 100 mL topical suspension. Subjects’ compliance with enema retainment (volume and time) was expected to affect the exposure of mesalazine. Unfortunately, the data regarding subjects’ compliance with enema retainment, including mesalazine enema and any other form of enemas, were very limited. In the current study, subjects were required to retain the enema for more than 8 hours (but no more than 10 hours), and the diaper weighing method was used to evaluate subjects’ compliance with enema retainment. Good compliance was considered if the diaper weight increased by no more than 20 g following the first and last dose administration, and no more than 50 g after the second to 6^th^ dose. All subjects demonstrated good compliance by adherence to all protocol details. After each administration, all the subjects retained enema for more than 8 hours, and the actual diaper weight increased by no more than 6.5 g. As a result, the systemic absorption of mesalazine enema was approximately 27.77% as measured by urinary excretion, which was higher than the previous report [12].

The AEs reported in this study did not show any trend as the mesalazine enema was administered from 1 to 7 days. This is consistent with the published report [20]. All AEs were mild and resolved spontaneously without sequelae. No subject withdrew from the study due to AEs. Therefore, the 1 g/100 mL mesalazine enema was well tolerated in healthy Chinese subjects.

## Conclusions

This study evaluated the safety and PK of 1 g/100 mL mesalazine enema in healthy Chinese subjects for the first time. After single- and multiple-dose administration, no clinically significant systemic accumulation (R = 1.09) or exposure (cumulative urinary excretion rate of parent and major metabolite = 27.77%) was observed. The 1 g/100 mL mesalazine enema showed good safety and tolerability in healthy Chinese subjects. These findings support further well-designed randomized clinical trials in Chinese patients.

## Supporting information

S1 Checklist (PDF)

S1 File (PDF)
