# Supplementary material for: Pharmacokinetic and safety profiles of mesalazine enema in healthy Chinese subjects: A single- and multiple-dose study
Source: PLoS One. 2024 Feb 2;19(2):e0296940. doi: 10.1371/journal.pone.0296940 (PMC10836682; doi:10.1371/journal.pone.0296940)
Supplement: S1 File — (PDF) [file pone.0296940.s002.pdf]

临床试验方案

中国健康受试者单次和多次给予颁得斯安®灌肠液后的药代动力学和安全性的开放研究

000166

欧盟临床试验数据库编号： 不适用

新药临床申请编号: 2012L01750

研究药物: 试验药
美沙拉秦灌肠液，1 g/100 mL
(颇得斯安®灌肠液)

适应症： 左半结肠溃疡性结肠炎

分期： I

申办者的名称和地址： 辉凌医药研究（北京）有限公司
中国北京市东城区建国门内大街 28 号民生金融中心 B 座 1001-1 室，邮编 100005
电话：+86 10 85295338
传真：+86 10 85295335

GCP 声明： *本试验将按照 GCP 要求进行。*

本文件中包含的信息为机密，归 ***Ferring International Pharma-Science Center (China) Company Limited [辉凌医药研究（北京）有限公司]*** *或* ***Ferring Group（辉凌集团）*** *所属公司所有。未经* ***Ferring International Pharma-Science Center (China) Company Limited [辉凌医药研究（北京）有限公司]*** *或* ***Ferring Group（辉凌集团）*** *所属公司授权主管事先书面同意，本文件中包含的信息不得以任何形式透露给任何第三方。*

方案概要

**试验题目**<br><br>中国健康受试者单次和多次给予颇得斯安®灌肠液后的药代动力学和安全性的开放研究	
**组长单位研究者**<br><br>张菁 教授，博士	
**研究中心**<br><br>复旦大学附属华山医院，上海市乌鲁木齐中路 12 号	
**计划试验周期**<br><br>首名受试者首次访视(FPFV)：<br>2015 年 6 月<br>末名受试者末次访视(LPLV)：<br>2015 年 9 月	**临床分期：**<br><br>I
**背景/原理**<br><br>美沙拉秦是轻度至中度活动性溃疡性结肠炎诱导缓解和维持治疗的主要一线药物。截至 2014 年 2 月，辉凌医药产品美沙拉秦直肠混悬液颇得斯安®灌肠液已在 71 个国家得到批准。<br>本研究是颇得斯安®灌肠液中国临床研发项目的一部分，目的是确定颇得斯安®灌肠液在中国健康受试者中单次和多次给药的药代动力学（PK）和安全性。	
**试验目的**<br><br>**主要目的：**<br>确定颇得斯安®灌肠液单次和多次直肠给药后，美沙拉秦在中国健康受试者中的药代动力学。<br>**次要目的：**<br>确定颇得斯安®灌肠液直肠给药后的尿药排泄。<br>评价颇得斯安®灌肠液在中国健康受试者中的单次和多次直肠给药后的安全性。	

评价指标

主要指标

- 药代动力学：
  - 单次给药期（第 1 次给药后）：美沙拉秦的 AUC_t、C_{max}、t_{max}。
  - 多次给药期（第 5 次给药后）：美沙拉秦的 AUC_{tau}、C_{max}、t_{max}。
  - 多次给药期（第 7 次给药后）：美沙拉秦的 AUC_{tau}、C_{max}、t_{max}。

次要指标

- 药代动力学：
  - 单次给药期（第 1 次给药后）：美沙拉秦的 AUC、CL/F、Vz/F、λ_z 和 $t_{½}$。
  - 多次给药期（第 5 次给药后）：美沙拉秦的 AUC、CL/F、Vz/F、C_{avg}、λ_z 和 $t_{½}$。
  - 多次给药期（第 7 次给药后）：美沙拉秦的 AUC、CL/F、Vz/F、C_{avg}、λ_z 和 $t_{½}$。
  - 美沙拉秦第 7 次给药后 24 h 内尿液中排泄的美沙拉秦和 N-乙酰基美沙拉秦的累积排泄量（Ae）。
  - 美沙拉秦第 7 次给药后 24 h 内以美沙拉秦和 N-乙酰基美沙拉秦形式经尿排出的药量占给药量的比例 （Fe）。
- 不良事件的类型、频率以及强度。
- 具有显著临床意义的生命体征、12 导联心电图（ECG）、临床化学、血液学和尿液分析的变化。

方法学

这是一项单次和多次给药的药代动力学开放研究。本研究有一个剂量组，由至少 8 名中国健康受试者组成。

筛选期为第-14 天至第-1 天。第-14 天至第-2 天，所有受试者将接受身体检查和实验室检测，包括一般体格检查、生命体征、12 导联心电图、直肠指检、粪便检查、血清妊娠试验（女性）、血清学/病毒学、临床化学、血液学、尿液分析、尿液药物筛查、酒精呼吸测试以及灌肠液保留测试（30 分钟）。

通过第-14 天至-2 天测试的受试者将于第-1 天进入试验中心。在第-1 天核对入选/排除标准，包括生命体征、血清妊娠试验（女性）、尿液药物筛查、酒精呼吸测试、临床化学、血液学、尿液分析以及 8 h 的灌肠液保留测试（包括灌肠液保留试验之前用温水灌肠排空肠道）。通过第-1 天测试的受试者可进行 IMP 给药。

合格的受试者将进入给药期（第 1 至 8 天）。在第 1-7 天晚上 9-10 点接受颇得斯安®灌肠液（1 g/100 mL）直肠给药，每天一次，并要保留灌肠液至 IMP 给药后 8 h 以上。第 1 次和第 7 次 IMP 给药约 2 h 使用温水灌肠排空肠道。在直肠给予 500~1000 mL 纯水后，受试者会被要求如厕排空肠道。第 2 至 6 次 IMP 给药前约 2 h，要求受试者如厕并尽可能排空肠道，但不使用温水灌肠。

用于美沙拉秦分析的血样分别在第 1 次、第 5 次和第 7 次给药后 24 h 内收集。三次给药对应的采样时间为：给药前即刻和给药后 0.5、1、2、3、4、5、6、7、8、10、12、14、16、20、24 h（下次给药前）。每个时间点约采血 4 mL。

采集尿液用于美沙拉秦及其主要代谢物 N-乙酰基美沙拉秦的分析。在第 1 次给药前采集 5~10 mL 空白尿液，第 7 次给药后分别于 0-12 h 和 12-24 h 时间段内采集所有排出的尿液。

给药期（第 1 至 8 天）每天测量生命体征。临床化学、血液学和尿液分析于第 2 天进行。

在第 9 天，受试者将进行试验结束检查，包括体格检查（身高除外）、生命体征、12 导联心电图、血清妊娠试验（女性）、临床化学、血液学和尿液分析。仅当试验结束检查结果为“正常”或“异常，不具有临床意义”时，受试者才能离开试验中心。如果此时有任何指标结果为“异常，具有临床意义”，受试者离开试验中心前，由研究者决定采取其他措施。

受试者数量

本试验未对样本数量进行正式计算。然而，根据 CFDA《化学药物临床药代动力学研究技术指导原则》，8 名受试者被认为能够提供足够的美沙拉秦药代动力学信息。假设脱落率为 20%，将招募 10 名合格受试者。

退出本试验的受试者可以被替换，以确保至少有 8 名受试者完成研究，其中每个性别至少 2 名受试者。

主要入选/排除标准

满足所有入选标准并且不满足任何一条排除标准的中国受试者：

入选标准

1. 签署书面知情同意文件（ICD）
2. 年龄为 18-45 岁。受试者签署 ICD 时，必须至少为 18 岁（包括 18 岁生日）且不大于 45 岁（截止 46 岁生日前一天）
3. 体重≥50 kg（男性）或≥45 kg（女性），体重指数（BMI）大于或等于 19.0 但不能超过 24.0 kg/m^2
4. 根据病史、体格检查、12 导联心电图、生命体征及实验室检查结果，受试者身体健康
5. 根据直肠指检和粪便检查结果，受试者的直肠和肛门处于健康状态
6. 根据病史和正常的排便频率（最近 3 个月内每天一次至每周三次），受试者的肠道处于健康状态
7. 第-14 天至第-2 天，能够保留一剂安慰剂灌肠液至少 30 分钟，纸尿裤重量增加不超过 20 g
8. 第-1 天，能够保留一剂安慰剂灌肠液至少 8 h，且纸尿裤重量增加不超过 20 g
9. 在筛选时，人类免疫缺陷病毒抗体（HIV-Ab）、乙肝表面抗原（HBs-Ag）、丙肝病毒抗体（HCV-Ab）和梅毒螺旋体抗体（由 TPPA 测试检测）的病毒学/血清学检查结果均为阴性
10. 在筛选时，尿液药物筛查和酒精呼气测试均为阴性
11. 从签署知情同意文件到试验结束检查后 3 天，受试者同意使用一种屏障避孕方法（例如，避孕套）
12. 不吸烟者或轻度吸烟者（每天不到五支烟，或者相当量）。如果是轻度吸烟者，入住试验中心期间禁止吸烟
13. 对于女性受试者：a）在筛选前 6 个月内，正常月经周期，平均为 28-35 天，b）在筛选

前 6 个月内，正常月经期 3-7 天，以及 c）月经结束日在试验第-1 天之前的 7 天内

排除标准

1. 有临床意义的肾、肝、胃肠道、心血管或肌肉骨骼系统的现病史或既往史，或者有临床意义的精神、免疫、内分泌或代谢性疾病的现病史或既往史
2. 过去 5 年内患有癌症，经过充分治疗的皮肤基底细胞癌和鳞状细胞癌除外
3. 重度变态反应或过敏性反应的现病史或既往史，例如对水杨酸类过敏
4. 正在妊娠或哺乳
5. 过去两年内药物滥用史或酗酒史，或者当前药物滥用或酗酒（＞40 g 酒精/天相当于＞1 L 啤酒/天、0.5 L 红酒/天或 6 杯（2 cL）烈酒/天）
6. 既往胃肠手术史
7. 肛门直肠疾病现病史或既往史，包括瘘管、结构异常、以及影响灌肠液保留能力的直肠张力疾病
8. 首次使用美沙拉秦前 2 周内或者药物的 5 个半衰期内（以时间较长者为准），服用过处方药、非处方（OTC）药或中药。治疗细菌或真菌感染的局部用药可被允许，前提是在首次使用 IMP 之前停止使用。
9. 筛选前 12 周内参加过任何临床试验，或筛选前更长时间内参加过临床试验且研究者认为所参加临床试验可能会影响本试验结果
10. 筛选前 12 周内每天饮用大量含有咖啡因的饮料（例如，五杯以上咖啡或者相当量），有在研究期间出现戒断症状的风险，可能混淆安全性评价
11. 第一天给药前 12 周内，献血或者大量失血（≥500 mL）
12. 曾被招募至本研究中并使用了至少一剂 IMP
13. 精神上无行为能力或者语言障碍，难以充分理解或配合
14. 月经期可能与计划给药期（第 1 至 8 天）重合的女性受试者
15. 因其他任何原因，被研究者认为不适合参加本研究

药物

研究药物（IMP）：

美沙拉秦灌肠液（颇得斯安®灌肠液，1 g/100 mL）

非研究药物（NIMP）：
安慰剂灌肠液（100 mL）

给药持续时间

受试者在第-1 天进入试验中心。颇得斯安®灌肠液将在第 1-7 天给药，每天一次。在第 1 天开始采集血样，并在第 8 天结束。尿样采集时间为第 1 天及第 7-8 天。受试者将在第 9 天离开试验中心。入住试验中心的总时长约为 10 天。

试验流程/评价

签署知情同意文件（ICD）后、在筛选时，所有受试者均接受一般体格检查、生命体征、12 导联心电图、直肠指检、粪便检查、血清妊娠试验（女性）、血清学/病毒学、临床化学、血液学检查、尿液分析和尿液药物筛查和酒精呼吸测试。将进行灌肠液保留测试（30 分钟）以排除对直肠给药敏感的受试者（必须在直肠指检之前进行灌肠液保留测试）。给予受试者一剂安慰剂灌肠液（100 mL）。将安慰剂灌肠液预热至 30~37℃，然后给药，给药方式与 IMP 相同。受试者使用称重后的纸尿裤。安慰剂灌肠液给药 30 分钟后再次称量纸尿裤的重量。应在脱掉纸尿裤后 10 分钟内称重。合格受试者应保留灌肠液至少 30 分钟，且纸尿裤重量增加小于或等于 20 g。检查月经史（女性）。合格女性受试者的月经期不太可能与给药期重合。根据第-14 天至第-2 天获得的受试者信息，核对入选/排除标准（参照表 1 试验流程图）。

第-1 天，受试者要接受入选/排除标准核对，包括生命体征、血清妊娠试验（女性）、尿液药物筛查、酒精呼气测试、临床化学、血液学检查、尿液分析和月经史（女性）。不过，如果第-1 天前 7 天内（即，第-8 天至第-2 天）进行了临床化学、血液学、尿液分析，则不再进行这些检查。第-1 天，进行灌肠液保留测试（8 h）（在测试前约 2 h 先用温水灌肠排空肠道）。第一次 IMP 给药前至少 22 h 给予受试者安慰剂灌肠液（100 mL）。受试者使用称重后的纸尿裤。安慰剂灌肠液给药 8 h 后再次称量纸尿裤的重量。应在脱掉纸尿裤后 10 分钟内称重。给予 NIMP 后 8 h 内需排尿的受试者将在床上使用便盆。合格受试者应保留灌肠液至少 8 h，且纸尿裤重量增加小于或等于 20 g。合格受试者将被视为筛选成功并进入

IMP给药阶段。

给药期间（第1-8天），受试者将在第1-7天晚上9-10点接受颇得斯安®灌肠液（1 g/100 mL）直肠给药，每天一次，并要保留灌肠液至IMP给药后8 h以上。将实施以下程序，提高并评价IMP保留的依从性：

- 给药前2 h（即晚上7-8点），受试者禁食禁水，一直到给药后8 h（即第二天上午5-6点）。
- 每次用药前约2 h，会要求受试者如厕，尽可能排空肠道。在第1次和第7次给药前，将使用温水灌肠以便排空肠道，但第2次至第6次给药前不使用温水灌肠。
- 每次用药前约10分钟都要求如厕以排空膀胱。
- 将带铝箔包装的IMP预热至30~37 ℃，擦干后，拆掉铝箔包装并称重。给予IMP后，再次称量使用过的IMP的重量。记录未使用和已使用IMP的重量。
- 使用成人纸尿裤对IMP保留的依从性进行评价。测量并记录新（未使用过的）纸尿裤的重量，之后让受试者使用此纸尿裤。给药8 h后，再次称取纸尿裤的重量。应在脱掉纸尿裤后10分钟内称重。如果第1剂或第7剂给药后纸尿裤重量增加20 g以上，第2-6剂某一剂给药后纸尿裤重量增加50 g以上，则出现该情况的受试者将退出本试验。
- 给药后8 h内需排尿的受试者将在床上使用便盆。
- 测量纸尿裤重量后，要求受试者在给药后8-10 h时间段内如厕，并记录排便时间（记录为进、出厕所的时间）。

用于美沙拉秦分析的血样分别在第1次、第5次和第7次给药后24 h内收集。3次给药对应的采样时间为：给药前即刻和给药后0.5、1、2、3、4、5、6、7、8、10、12、14、16、20、24 h（下次给药前）。每个时间点采血量约4 mL。

采集尿液用于美沙拉秦及其主要代谢物N-乙酰基美沙拉秦的分析。在第1次给药前采集5~10 mL空白尿液，第7次给药后分别于0-12 h和12-24 h时间段内采集所有排出的尿液。

给药期（第1至8天）每天测量生命体征。临床化学、血液学和尿液分析于第2天进行。

第 9 天，受试者接受试验结束检查，包括体格检查（体重除外）、生命体征、12 导联心电图、血清妊娠试验（女性）、临床化学、血液学检查和尿液分析。仅当试验结束检查结果为“正常”或“异常，不具有临床意义”时，受试者才能离开试验中心。如果此时有任何指标结果为“异常，具有临床意义”，受试者离开试验中心前由研究者决定采取其他措施。

入住试验中心的时间为第-1 天至第 9 天。每位受试者总共采集约 260 mL 全血（PK 血样约 200 mL，安全性检查用血样约 60 mL）。第-1 天至第 9 天提供标准餐。

研究期间不允许合并用药，治疗不良事件所必需的除外。如果合并用药，必须进行记录。

统计方法

样本量：

本试验未对样本数量进行正式计算。然而，根据 CFDA《化学药物临床药代动力学研究技术指导原则》，8 名受试者被认为能够提供足够的美沙拉秦药代动力学信息。假设脱落率为 20%，将招募 10 名合格受试者。退出试验的受试者可被替换，以确保至少有 8 名受试者完成试验（每个性别至少有 2 名受试者）。

药代动力学分析：

单次给药：用非房室分析求算 PK 参数。PK 参数将根据第 1 天至第 2 天获得的测定结果进行计算，并在总结表、图表和列表中列出。

多次给药：用非房室分析求算 PK 参数。PK 参数将分别根据第 5-6 天和第 7-8 天获得的测定结果进行计算，并在总结表、图表和列表中列出。计算第 7 次给药后的 Ae 和 Fe，并在总结表和列表中列出。

根据第 1 次给药后的 AUC（如未计算 AUC，则根据 AUC_t）和第 7 次给药后的 AUC_{tau}，评价药物在体内的蓄积。

安全性分析：

不良事件将在总结表和列表中列出。安全性实验室检测、生命体征和 12 导联心电图数据将在总结表和列表中列出，包括相对于基线的变化。数据还将在散点图和曲线图中列出。

表 1 试验流程图

评估	筛选		给药期								试验结束检查
研究日	-14至-2	-1	1	2	3	4	5	6	7	8	9
知情同意	X										
入选/排除标准	X	X	X								
人口统计学	X										
病史	X										
体格检查	X										X[a]
直肠指检	X										
粪便检查	X										
生命体征[b]	X	X	X	X	X	X	X	X	X	X	X
12导联心电图	X										X
血清妊娠试验 (女性)[c]	X	X									X
尿液药物筛查	X	X									
酒精呼吸测试	X	X									
血清学/病毒学[c]	X										
临床化学[c]	X	X[d]		X[e]							X
血液学[c]	X	X[d]		X[e]							X
尿液分析[c]	X	X[d]		X[e]							X
灌肠液保留测试 (30分钟)[f]	X										
月经史检查 (女性)[g]	X	X									
灌肠液保留测试 (8 h)[h]		X									
进入试验中心		X									
标准餐		X	X	X	X	X	X	X	X	X	X
禁食禁水[i]		X	X	X	X	X	X	X	X		
肠道排空[j]		X	X	X	X	X	X	X	X		
膀胱排空[k]		X	X	X	X	X	X	X	X		
成人纸尿裤应用和重量测量[l]		X	X	X	X	X	X	X	X		
(N)IMP称重[m]		X	X	X	X	X	X	X	X		
给予IMP[n]			X	X	X	X	X	X	X		
PK血样采集[o]			X	X			X	X	X	X	
尿样采集[p]			X						X	X	
IMP保留[q]			X	X	X	X	X	X	X		
使用便盆[r]		X	X	X	X	X	X	X	X		
排便以及排便时间记录[s]			X	X	X	X	X	X	X	X	
不良事件	X[t]	X	X	X	X	X	X	X	X	X	X
既往/合并用药	X	X	X	X	X	X	X	X	X	X	X
离开研究中心[u]											X

[a] 试验结束检查时不测量体重。

[b] 第-14 天至-2 天、第-1 天、给药期（第 1-8 天）每天、及作为试验结束检查的一部分，在每天上午 8-10 点，休息 5 分钟后，测量生命体征（血压、脉率、腋下温度和呼吸频率）。测量仰卧位血压。

[c] 在当地实验室进行分析。

[d] 如果在第-1 天前 7 天内（即第-8 天至第-2 天）进行了这些检查，那么第-1 天就不再进行。

[e] 第 2 次给药前采集血液和尿液样本。

[f] 必须在 DRE 之前进行灌肠液保留测试（30 分钟，第-14 天至第-2 天进行）。给予受试者一剂安慰剂灌肠液（100 mL）。将安慰剂灌肠液预热至 30~37℃，然后给药，给药方式与 IMP 相同。受试者使用称重过的纸尿裤。给药 30 分钟后再次称量纸尿裤的重量。应在脱掉纸尿裤后 10 分钟内称重。合格的受试者应保留灌肠液至少 30 分钟，纸尿裤的重量增加不超过 20 g。

[g] 第-14 天至第-2 天和第-1 天检查月经史（女性）。合格女性受试者的月经期不可能与给药期重合。

[h] 第-1 天，进行灌肠液保留测试（8 h）。第 1 剂 IMP 给药前至少 22 h 给予受试者安慰剂灌肠液（100 mL）。所有与 IMP 依从性改善和评价有关的步骤均与 IMP 给药相同，包括禁食和禁水 [i]，肠道排空 [j]，膀胱排空 [k]，成人纸尿裤应用和重量测量 [l]，（N）IMP 称重 [m]，（N）IMP 保留 [p]，使用便盆 [q]，排便和排便时间记录 [r]。合格受试者应保留灌肠液至少 8 h，纸尿裤重量增加小于或等于 20 g。

[i] 受试者在给药前 2 h 到给药后至少 8 h 禁食禁水。

[j] 第-1 天在第-1 天 NIMP 给药前、第 1 次和第 7 次 IMP 给药前约 2 h 受试者使用温水灌肠，以促进肠道排空。在第 2 次至第 6 次 IMP 给药前约 2 h，会要求受试者如厕，尽量排空肠道，但不使用温水灌肠。

[k] NIMP 给药前（第-1 天）和每次 IMP 用药前（第 1 至 7 天）约 10 分钟都要求如厕以排空膀胱。

[l] 使用成人纸尿裤，对（N）IMP 保留的依从性进行评价。测量并记录新的（未使用过的）纸尿裤的重量，之后让受试者使用此纸尿裤。给药 8 h 后，再次称取纸尿裤的重量。应在脱掉纸尿裤后 10 分钟内称重。

[m] 将铝箔包装的（N）IMP 预热至 30~37℃，擦干后，拆掉铝箔包装并称重。给予（N）IMP 后，再次称量使用过的（N）IMP 的重量。记录未使用和已使用（N）IMP 的重量。

[n] 在第 1-7 天晚上 9-10 点左右给予 IMP。

[o] 在第 1 次、第 5 次和第 7 次给药后 24 h 内重复采集用于美沙拉秦分析的血样。第 1 次、第 5 次和第 7 次给药对应的采样时间为：给药前即刻，及给药后 0.5、1、2、3、4、5、6、7、8、10、12、14、16、20、24 h（下次给药前）。

[p] 对于药物排泄测定，在第 7 次给药后（第 7 次给药后 0-12 h 和 12-24 h 内）采集尿样。在第 1 次给药前采集空白尿样。

[q] 应当保留 IMP 到给药后至少 8 h。

[r] 给药后 8 h 内需排尿的受试者将在床上使用便盆。

[s] 测量纸尿裤重量后会要求受试者在给药后 8-10 h 如厕，并记录排便时间（记录出、入为厕所的时间）。

[t] 签署 ICD 后开始收集 AE。

[u] 如果试验结束检查的结果被认为是“正常”或“异常但无临床意义”，受试者才可离开试验中心。如果此时任何指标被认为是“异常且有临床意义”，受试者离开试验中心前由研究者决定采取其他措施。

目录

页码

方案概要........2

表目录........18

图目录........18

缩略词和术语定义列表........19

1 前言........23

1.1 背景........23

1.2 进行本试验的科学依据........24

1.3 收益/风险评估........24

2 试验目的和评价指标........25

2.1 目的........25

2.2 评价指标........26

3 研究计划........26

3.1 总体试验设计........26

3.1.1 试验设计图........26

3.1.2 总体设计和对照方法........27

3.1.3 试验时间表........29

3.2 计划的研究中心和受试者数量........29

3.3 中期分析........29

3.4 数据监管委员会 (DMC)........30

3.5 总体试验设计讨论........30

3.5.1 试验设计........30

3.5.2 评价指标的选择........31

3.5.3 设盲........31

3.5.4 研究药物剂量的选择........31

3.5.5 受试者给药剂量和给药时间的选择........31

3.5.6 试验人群的选择........32

3.5.7 随访流程........32

4 研究人群的选择........32

4.1 试验人群 32
4.1.1 入选标准 32
4.1.2 排除标准 33
4.2 受试者分配至给药组的方法 34
4.2.1 招募 34
4.3 限制条件 34
4.3.1 既往和合并治疗 34
4.3.2 禁用治疗 35
4.3.3 其他限制条件 35
4.4 退出标准 36
5 给药 37
5.1 给予的药物 37
5.1.1 研究药物（IMP） 37
5.1.2 非研究药物（NIMP） 38
5.2 药品的特征与来源 38
5.2.1 研究药物 38
5.2.2 非研究药物 38
5.3 包装和标签 39
5.3.1 研究药物 39
5.3.2 非研究药物 39
5.4 药物贮藏和使用条件 39
5.4.1 研究药物 39
5.4.2 非研究药物 39
5.5 设盲/揭盲 39
5.6 治疗依从性 40
5.6.1 药物的发放和清点 40
5.6.2 灌肠液保留评估 40
5.7 医疗用品和辅助用品的归还与销毁 40
6 试验流程 40
6.1 试验流程图 40
6.2 筛选 43

6.3 给药期......44
6.4 试验结束检查......46
7 试验评估......46
7.1 评价指标相关的评估......47
7.1.1 药代动力学评估......47
7.1.2 生命体征......47
7.1.3 心电图......47
7.1.4 不良事件......48
7.1.5 临床化学......48
7.1.6 血液学......49
7.1.7 尿液分析......50
7.1.8 体格检查......51
7.2 其他评估......51
7.2.1 直肠指检......51
7.2.2 粪便检查......51
7.2.3 血清学和病毒学......52
7.2.4 血清妊娠试验（女性）......52
7.2.5 尿液药物筛查和酒精呼气测试......52
7.2.6 人口统计数据和基线数据......53
7.2.7 病史......53
7.2.8 既往和伴随用药......53
7.2.9 灌肠液保留测试（30分钟）......53
7.2.10灌肠液保留测试（8 h）......53
7.3 药物浓度测定......54
7.4 生物样本处理......54
8 不良事件......55
8.1 不良事件定义......55
8.2 不良事件的收集和记录......56
8.2.1 不良事件的收集......56
8.2.2 不良事件的记录......56
8.3 妊娠和妊娠转归......59

8.4 严重不良事件 59
8.4.1 严重不良事件的定义 59
8.4.2 严重不良事件的收集、记录和报告 60
8.5 不良事件和严重不良事件的随访 62
8.5.1 试验期间发生的不良事件的随访 62
8.5.2 末次访视后发生的严重不良事件的收集 62
9 统计方法 62
9.1 样本量的确定 62
9.2 受试者分析 62
9.3 试验方案偏离 63
9.4 分析集 63
9.4.1 意向治疗分析数据集（ITT） 63
9.4.2 全分析集（FAS）/疗效人群 63
9.4.3 符合方案数据集 (PP) 63
9.4.4 安全性数据集 63
9.5 试验人群 63
9.5.1 人口统计学和其他基线特征 63
9.5.2 病史, 既往 / 合并用药和其他安全评价 64
9.5.3 给药依从性 64
9.6 评价指标评估 64
9.6.1 一般考虑事项 64
9.6.2 主要药代动力学指标 64
9.6.3 次要药代动力学指标 65
9.7 给药的依从性 65
9.8 安全性 65
9.8.1 一般考虑事项 65
9.8.2 不良事件 66
9.8.3 实验室检查数据 67
9.8.4 生命体征 67
9.8.5 ECG 68
9.8.6 体格检查 68
9.9 中期分析 68

10 数据处理 68
10.1 原始数据和原始文件 68
10.2 CRF 69
10.3 数据管理 69
10.4 附加信息的提供 70
11 监查程序 70
11.1 定期监查 70
11.2 稽查和视查 70
11.3 受试者信息的机密性 71
12 试验操作的变更 71
12.1 试验方案修订 71
12.2 试验试验方案偏离 71
12.3 提前终止试验 71
13 报告和发表 72
13.1 临床试验报告 72
13.2 研究数据的机密性和所有权 72
13.3 发表和公开披露 72
13.3.1 发表政策 72
13.3.2 公开披露政策 72
14 伦理监管 73
14.1 伦理委员会 73
14.2 监管当局授权/批准/通知 73
14.3 试验结束和试验结束通知 73
14.4 试验的伦理行为准则 73
14.5 受试者告知信息和知情同意 73
14.6 受试者信息卡 74
14.7 依从性参考文件 74
15 责任和保险 74
15.1 ICH-GCP/GCP责任 74
15.2 责任和保险 74
16 存档 74

16.1 研究者文档 74
16.2 试验主文档 75

17 参考文献 76

附件 77

附件1 研究药物给药介绍 77
附件2 使用温水灌肠排空肠道的说明 78

表目录

表 1 试验流程图........10
表 2 试验设计示意图........27
表 3 安全性实验室参数–临床化学........49
表 4 安全性实验室参数–血液学........50
表 5 安全性实验室参数–尿液分析........51

图目录

图 1 试验示意图........42
图 2 与 IMP 给药依从性提高和评估相关的程序........44

缩略词和术语定义列表

ADR	药物不良反应<br>Adverse Drug Reaction
AE	不良事件<br>Adverse Event
ATC	药品解剖学、治疗学及化学分类系统<br>Anatomical Therapeutic Chemical Code
BMI	体重指数<br>Body mass index
CD	克罗恩病<br>Crohn’ s disease
CFDA	中国国家食品药品监督管理总局<br>China Food and Drug Administration
CRF	病例报告表<br>Case report form
CRO	合同研究组织<br>Contract Research Organization
DMC	数据监管委员会<br>Data Monitoring Committee
DRE	直肠指检<br>Digital rectal examination
ECG	心电图<br>Electrocardiogram
EC	伦理委员会<br>Ethics Committee
EU	欧盟<br>European Union
FAS	全分析集<br>Full Analysis Set
GCP	药品临床试验管理规范<br>Good clinical practice
GMP	药品生产质量管理规范<br>Good Manufacturing Practice
IBD	炎症性肠病<br>Inflammatory bowel disease
ICD	知情同意文件<br>Informed Consent Documents
ICH	人用药品注册技术要求国际协调会议

	International Conference on Harmonization
INN	国际非专利药名<br>International non-proprietary name
IEC	独立伦理委员会<br>Independent Ethics Committee
IMP	研究药物<br>Investigational Medicinal Products
IND	药品临床试验(申请)<br>Investigational New Drug (Application)
ITT	意向治疗分析集<br>Intent-to-treat
LLQ	定量下限<br>Lower limit of quantification
MedDRA	药事管理的标准医学术语集<br>Medical Dictionary for Regulatory Activities
ND	未检出<br>Not detectable
NIMP	非研究药物<br>Non-investigational Medicinal Products
PK	药物代谢动力学（的）<br>Pharmacokinetic(s)
PP	符合方案<br>Per Protocol
SAE	严重不良事件<br>Serious Adverse Event
SDV	原始数据核对<br>Source data verification
SOC	系统器官分类<br>System organ class
SUSAR	疑似非预期严重不良反应<br>Suspected Unexpected Serious Adverse Reaction
UC	溃疡性结肠炎<br>Ulcerative colitis
WHO	世界卫生组织<br>World Health Organisation

术语解释

入组	签署知情同意文件（ICD）时，受试者入组。
试验持续时间	从签署 ICD 至离开试验中心的时间。
给药时间	从首次给予试验用药物（IMP)至最后一次 PK 血样采集访视的时间。
试验结束	最后一次与进行试验的最后一名受试者联系。

药代动力学术语

NCA 非房室分析

λ_z 一级消除速率常数，用血浆药物浓度-时间曲线末端（对数线性）部分中，以血浆药物浓度的对数值对时间进行线性回归进行估算。

AUC 零到无限大时间的血浆药物浓度-时间曲线下面积，按照以下公式计算：

$$AUC = AUC_t + \frac{C_{last}}{\lambda_z}$$

其中 C_{last} 为最后一次可测定的血浆药物浓度。

AUC_t 从时间零至时间 t 的血浆药物浓度-时间曲线下面积，其中 t 为受试者显示定量下限以上浓度的最后一个时间点（LLQ；t_{last} 时最后一次可测定（非零）浓度的时间)。按照以下公式，采用线性梯形（线性插值）法计算 AUC_t：

$$AUC\Big|_{t_1}^{t_2} = \delta t \times \frac{C_1 + C_2}{2}$$

其中 C_1 为时间点一（t_1）时的血浆浓度，C_2 为时间点二（t_2）时的血浆浓度，δt 为（t_2-t_1）。

F 生物利用度

CL 清除率

CL/F 表观清除率=剂量/AUC。

C_{max} 最大血浆药物浓度（观测值）。

t_{max}	口服给药后达到 C_{max} 的时间。
$t_{½}$	终末消除半衰期。
Vz/F	表观分布容积=λ_z 剂量/AUC。
C_{avg}	多次给药期的平均血浆药物浓度=AUC_{tau}/时间 tau。
Ae	尿液中排泄的美沙拉秦和 N-乙酰基美沙拉秦的总蓄积量
Fe	以美沙拉秦和 N-乙酰基美沙拉秦形式经尿排出的药量占给药量的比例

1 前言

1.1 背景

溃疡性结肠炎（ulcerative colitis, UC），炎症性肠病（inflammatory bowel disease, IBD）的一种，是病因和发病机制复杂的一种慢性炎症性肠病，特征是从直肠起始的结肠粘膜连续性炎症。疾病程度可从直肠炎至左半结肠炎和广泛性结肠炎或全结肠炎。溃疡性结肠炎在女性比男性中稍常见。发病年龄呈双峰分布，峰值在 15-25 岁，在 55-65 岁有一个较小的峰，但该疾病可发生在任何年龄[1]。UC 在人群中的年度发病率，美国为每 100,000 人中 10.4-12 例病例，欧洲为每 100,000 人中 8 例病例。关于患病率，美国为每 100,000 人中 35-100 例病例，欧洲为每 100,000 人中 21-243 例病例。有一些数据表明过去 20 年间中国 UC 病例的例数快速增加，尽管尚未对基于人群的 UC 发病率进行广泛研究。在中国湖北省武汉市进行的一项近年来患病率的流行病学研究确定在中国的这一地区，年度发病率为 1.45 例/100,000 人[2]。基于中国的多医院病例计算，中国的 UC 患病率至少为 11.6 例/100,000 人[3]。

UC 的活动性发作明显特征是便血和大便粘液、腹泻以及表现为排便紧迫感的腹痛以及排便不尽感（里急后重）[4]。大多数重型病例中，可能发生全身症状，包括发热、食欲减退和体重减轻。在 UC 患者中，约 60%患者为轻度，25%为中度，15%为重度。据估计每 2 名 UC 患者中约有 1 名会在 1 年内复发，2 年内累积复发概率为 80%，10 年内为 95% [5]。

美沙拉秦是轻度至中度活动性溃疡性结肠炎诱导缓解和维持治疗的主要一线药物[6]。口服及直肠给药后美沙拉秦的治疗作用可能是由于对炎性肠组织的局部作用，而不是由于全身作用[7]。炎性肠组织内白细胞迁移增多、细胞因子生成异常、花生四烯酸代谢产物的产量增加（特别是白三烯 B_4）及自由基的形成增多可见于所有炎症性肠病患者。美沙拉秦具有体外和体内药理学作用，可抑制白细胞趋化，降低细胞因子和白三烯产量，并清除自由基[8-11]。目前尚不清楚其中哪种机制在美沙拉秦的临床疗效中发挥主导作用。

辉凌公司生产的药品美沙拉秦，商品名为颇得斯安®，1986 年在丹麦首次获得批准，获批剂型为颇得斯安®缓释片 250 mg 和颇得斯安®直肠混悬液 1 g/100 mL（灌肠液）。这些剂型在不久以后上市，并且在之后的几年内，几种延伸产品也获得批准：颇得斯安®缓释片 500 mg、颇得斯安®栓剂 1 g 以及颇得斯安®缓释颗粒 1 g 和 2 g。截止 2014 年 2 月，颇得斯安®直肠混悬液已在 71 个国家获得批准。

已使用颇得斯安®灌肠液完成了三项临床药代动力学研究。1 g/100 mL 美沙拉秦灌肠液给药后 4 h 进行的闪烁成像研究显示，在健康男性受试者中，72%的混悬液留在直肠和乙状结肠内，25%扩散到降结肠，其余 3%到达横结肠[12]。涉及血浆药代动力学和尿药回收率研究的其他两项试验显示，直肠给药后，美沙拉秦的全身生物利用度较低。根据稳态条件下的尿药回收情况，健康受试者使用直肠混悬液（灌肠液）后，约 15-20%的药物被吸收[7, 13]。不过，这些研究并未确定直肠混悬液给药后美沙拉秦的半衰期，其他研究显示静脉注射给药后美沙拉秦的半衰期为 0.5~1 h[14, 15]。

1.2 进行本试验的科学依据

本研究是颇得斯安®灌肠液中国临床研究的一部分，目的是确定中国健康受试者中的单次和多次给药后颇得斯安®灌肠液的药代动力学（PK）和安全性。

尽管颇得斯安®灌肠液被认为是一种局部疗法，但其全身生物利用度并非可忽略。健康受试者研究显示，颇得斯安®灌肠液的生物利用度为 15-20%。不过，尚不清楚直肠混悬液给药后美沙拉秦在中国人群中的全身暴露特征。本研究将确定美沙拉秦灌肠液（颇得斯安®灌肠液）在健康中国受试者中单次和多次直肠给药后的药代动力学特征，并证实其安全性（例如多次给药后的药物可能蓄积）。

1.3 收益/风险评估

自颇得斯安®首次于 1986 年获得批准以来，已经确立了广泛的安全性。在中国，颇得斯安®片剂和栓剂从 1993 年上市，在中国患者中也表现出类似的安全性和耐受性。

截止 2013 年 2 月 28 日，基于产品销量数据，估计全世界口服制剂和直肠制剂的总累计患者暴露量为 8,036,415 患者年，估计全世界颇得斯安®直肠制剂的累计患者暴露量为 432,533 患者年。估计暴露量时，假设口服制剂的每日剂量为 1.5 g，直肠制剂的每日剂量为 1 剂栓剂或 1 剂灌肠液，不论规格如何，并且 100%的已销售制剂都被使用。截止 2013 年 2 月 28 日，在临床试验中使用该药的患者人数约为 10,600，其中包括溃疡性结肠炎（UC）患者和克罗恩病（CD）患者（CD 是美沙拉秦的另一个适应症）。

由于本研究涉及健康受试者，因此参加研究不会有直接的健康收益。本研究将揭示美沙拉秦灌肠液在中国人群中的生理处置情况。

颇得斯安®已上市逾 25 年。根据颇得斯安®（所有剂型）临床研究和上市后监测，常见（≥1%且<10%）不良事件为头痛、腹泻、腹痛、恶心、呕吐和皮疹（包括荨麻疹、红斑疹）。这些不良反应都为轻度。

偶尔可能发生超敏反应和药物热。直肠给药后，可能发生瘙痒、直肠不适和排便急迫等局部反应。

选择参加这项试验的健康受试者可能发生上述任何一种已鉴别事件，或者发生既往未报告过的事件。不过，针对上述任一风险，参加这项研究的受试者发生有临床意义事件的可能性被认为较低，并且从伦理方面来讲是可接受的。由于参加研究会造成潜在的风险，因此本试验的潜在受试者将在知情同意过程中（即，阅读知情同意文件[ICD]和与研究者讨论时）被充分告知参加试验的可能风险，以便他们单独决定是否希望参加本研究。

2 试验目的和评价指标

2.1 目的

主要目的

确定颇得斯安®灌肠液单次和多次直肠给药后，美沙拉秦在中国健康受试者中的药代动力学。

次要目的

确定颇得斯安®灌肠液直肠给药后的尿药排泄。

评价颇得斯安®灌肠液在中国健康受试者中的单次和多次直肠给药后的安全性。

2.2 评价指标

主要指标

- 药代动力学：
  - 单次给药期（第 1 次给药后）：美沙拉秦的 AUC_t、C_{max}、t_{max}。
  - 多次给药期（第 5 次给药后）：美沙拉秦的 AUC_{tau}、C_{max}、t_{max}。
  - 多次给药期（第 7 次给药后）：美沙拉秦的 AUC_{tau}、C_{max}、t_{max}。

次要指标

- 药代动力学：
  - 单次给药期（第 1 次给药后）：美沙拉秦的 AUC、CL/F、Vz/F、λ_z和 $t_{½}$。
  - 多次给药期（第 5 次给药后）：美沙拉秦的 AUC、CL/F、Vz/F、C_{avg}、λ_z和 $t_{½}$。
  - 多次给药期（第 7 次给药后）：美沙拉秦的 AUC、CL/F、Vz/F、C_{avg}、λ_z和 $t_{½}$。
  - 美沙拉秦第 7 次给药后 24 h 内尿液中排泄的美沙拉秦和 N-乙酰基美沙拉秦的累积排泄量（Ae）
  - 美沙拉秦第 7 次给药后 24 h 内以美沙拉秦和 N-乙酰基美沙拉秦形式经尿排出的药量占给药量的比例
- 不良事件的类型、频率以及强度。
- 具有显著临床意义的生命体征、12 导联心电图（ECG）、临床化学、血液学和尿液分析的变化。

3 研究计划

3.1 总体试验设计

3.1.1 试验设计图

试验设计示意图见表 2。

表 2 试验设计示意图

筛选	给药期	试验结束检查
入选/排除	IMP 给药/<br>评估	评估
第-14 天至第-1 天*	第 1 天至第 7 天/第 1 天至第 8 天	第 9 天

***注**：本研究没有第 0 天。

3.1.2 总体设计和对照方法

这是在中国健康受试者中实施的一项开放、单次和多次给药研究。

受试者将接受每天一剂 IMP，连续 7 天。第 1 次给药后将考察单次给药的药代动力学。第 5 次和第 7 次给药后将考察多次给药的药代动力学。

计划进行三次研究访视：

- 第 1 次 IMP 给药前第-14 天至第-1 天进行的筛选。
- 持续 8 天（第 1 天至第 8 天）的一次住院访视，在此期间将进行 IMP 给药并采集血样和尿样。
- 一次试验结束检查，在第 9 天进行。

签署知情同意文件（ICD）后筛选期第-14 天至第-2 天，所有受试者均接受一般体格检查、生命体征、12 导联心电图、直肠指检、粪便检查、血清妊娠试验（女性）、血清学/病毒学、临床化学、血液学检查、尿液分析、尿液药物筛查和酒精呼吸测试。将进行灌肠液保留测试（30 分钟）以排除对直肠给药敏感的受试者（必须在 DRE 之前进行灌肠液保留测试）。给予受试者一剂安慰剂灌肠液（100 mL）。将安慰剂灌肠液预热至 30~37℃，然后以与 IMP 相同的方式给药。受试者使用称重过的纸尿裤。安慰剂灌肠液给药 30 分钟后再次称量纸尿裤的重量。应在脱掉纸尿裤后 10 分钟内称重。合格受试者应保留灌肠液至少 30 分钟，纸尿裤重量增加小于或等于 20 g。核查月经史（女性）。合格女性受试者的月经期需不太可能与给药期（第 1 至 8 天）重合。根据第-14 至第-2 天获得的受试者信息，核对入选/排除标准。

受试者将在第-1 天接受入选/排除标准核查，包括生命体征、血清妊娠试验（女性）、尿液药物筛查、酒精呼气测试、临床化学检查、血液学、尿液分析、月经史（女性）。不过，如果第-1 天前 7 天内（即，第-8 天至第-2 天）进行了临床化学检查、血液学和尿液分析，则不再进行这些检查。第-1 天，进行灌肠液保留测试（8 h）。（在测试前约 2 h 先用温水灌肠排空肠道。）第 1 剂 IMP 给药前至少 22 h 给予受试者安慰剂灌肠液（100 mL）。受试者使用称重后的纸尿裤。安慰剂灌肠液给药 8 h 后再次称量纸尿裤的重量。应在脱掉纸尿裤后 10 分钟内称重。给予 NIMP 后 8 h 内需要排尿的受试者将在床上使用便盆。合格受试者应保留灌肠液至少 8 h，纸尿裤重量增加小于或等于 20 g。合格受试者将被视为筛选成功并可以接受 IMP 给药。

给药期（第 1 至 8 天）受试者将在第 1-7 天晚上 9-10 点接受每天一次的颇得斯安®灌肠液（1 g/100 mL）直肠给药，并要求 IMP 给药后保留灌肠液至少 8 h。实施以下程序，以提高并评价 IMP 保留的依从性：

- 给药前 2 h（即晚上 7-8 点），受试者禁食禁水，一直到给药后 8 h（即第二天上午 5-6 点）。
- 每次用药前约 2 h，会要求受试者如厕，尽可能排空肠道。在第 1 次和第 7 次给药前，将使用温水灌肠以便排空肠道，但第 2 次至第 6 次给药前不使用温水灌肠。
- 每次用药前约 10 分钟都要求如厕以排空膀胱。
- 将带铝箔包装的 IMP 预热至 30~37 ℃，擦干后，拆掉铝箔包装并称重。给予 IMP 后，再次称量使用过的 IMP 的重量。记录未使用和已使用 IMP 的重量。
- 使用成人纸尿裤对 IMP 保留的依从性进行评价。测量并记录新（未使用过的）纸尿裤的重量，之后让受试者使用此纸尿裤。给药 8 h 后，再次称取纸尿裤的重量。应在脱掉纸尿裤后 10 分钟内称重。如果第 1 剂或第 7 剂给药后纸尿裤重量增加 20 g 以上，第 2-6 剂某一剂给药后纸尿裤重量增加 50 g 以上，则出现该情况的受试者将退出本试验。
- 给药后 8 h 内需排尿的受试者将在床上使用便盆。
- 测量纸尿裤重量后，要求受试者在给药后 8-10 h 时间段内如厕，并记录排便时间（记录为进、出厕所的时间）。

第 1 次、第 5 次和第 7 次给药后 24 h 内重复收集用于美沙拉秦分析的血样。三次给药对应的采样时间为：给药前即刻和给药后 0.5、1、2、3、4、5、6、7、8、10、12、14、16、20、24 h（下次给药前）。每个时间点约采血 4 mL。

采集尿液用于美沙拉秦及其主要代谢物 N-乙酰基美沙拉秦的分析。在第 1 次给药前采集 5~10 mL 空白尿液，第 7 次给药后分别于 0-12 h 和 12-24 h 时间段内采集所有排出的尿液。

从第 1 天至第 8 天每天测量生命体征。第 2 天测量临床化学、血液学、尿液分析。

在第 9 天，受试者将接受试验结束检查，包括体格检查（体重除外）、生命体征、12 导联心电图、血清妊娠试验（女性）、临床化学检查、血液学和尿液分析。如果试验结束检查的结果被认为是“正常”或“异常但无临床意义”，受试者才可离开试验中心。如果此时任何指标被认为是“异常且有临床意义”，那么在受试者离开前，将根据研究者的意见采取其他措施。

入住试验中心的时间为第-1 天至第 9 天。在此期间将提供标准饮食。

每名受试者约采样共计 260 mL（PK 血样约 200 mL，安全性检查用血样约 60 mL）。

研究期间不允许合并用药，为治疗不良事件所必需的除外。记录用于治疗不良事件的药物。

3.1.3 试验时间表

计划在 2015 年 6 月开始筛选。计划在 2015 年 6 月至 9 月进行 IMP 给药和 PK 血样和尿样采集。末名受试者末次访视的时间计划为 2015 年 9 月。

3.2 计划的研究中心和受试者数量

本研究将在中国的单个 I 期研究中心开展。两个性别 10 名合格受试者将入选本试验，确保 8 名受试者完成研究，其中至少每个性别 2 名受试者完成研究。

未完成研究的受试者可被替换，以确保有至少 8 名受试者完成研究。

3.3 中期分析

未计划进行正式统计期中分析。

3.4 数据监管委员会 (DMC)

本研究不设数据监测委员会。

3.5 总体试验设计讨论

3.5.1 试验设计

本研究设计是在中国健康受试者中实施的一项开放、单次和多次给药研究。

由于这是 IMP 的一项 PK 研究，不设对照，因此是一项开放研究。选择中国健康受试者作为试验人群的目的是反映 IMP 本身的特征，这也是中国国家食品药品管理总局（CFDA）的一般要求[16]。

仅选择一个剂量水平（更多信息见第 3.5.4 节），来反映用于临床的剂量水平。将研究第 1 次和第 7 次给药后的 PK，并对 PK 参数（第 1 次给药后的 AUC 和第 7 次给药后的 AUC_{tau}）进行比较，以便揭示多次给药后潜在的药物蓄积。

美沙拉秦直肠给药后的全身暴露率较低，并且取决于剂量、剂型和扩散程度。8 h 被认为是临床应用中通常的 IMP 保留时间。三次给药每次 PK 血样采集将持续 24 h，以获取给药间隔时间内美沙拉秦血药浓度-时间曲线。

本研究的主要目的是确定健康中国受试者经直肠单次和多次给予美沙拉秦（颇得斯安®，1 g/100 mL）后的药代动力学。比较单次给药（第 1 次给药）和多次给药（第 7 次给药）PK 参数（即 AUC）以评价多次给药后药物累积情况。因此，第 1 次和第 7 次与 IMP 给药的相关程序应相同，并且，还应消除可能影响美沙拉秦吸收的因素（除给药次数外）或者第 1 次和第 7 次给药时这些影响因素相同。因此，为了避免肠内容物对美沙拉秦吸收的潜在影响，第 1 次和第 7 次给药前使用温水灌肠将肠道排空。在临床应用中，建议颇得斯安®灌肠液给药前如厕。在本研究中，在第 2 次至第 6 次 IMP 给药前要求如厕，但不使用温水灌肠。第 2 次至第 6 次给药产生的体内药物蓄积（如果有）应反映临床实践中的蓄积情况，因为在临床实践中和本试验中使用了相同的给药前程序（即如厕）。

然而，温水灌肠的一个问题是，其程序与临床实践并不完全相同。尽管第 1 次和第 7 次给药后获得的 PK 特征反映了临床实践中最大吸收的可能，但是第 1 次或第 7 次给药后不能充分反映临床应用中 PK 特征的真实情况，即肠内可能有内容物的情况未被研究。因此还要对未进行温水灌肠的第 5 次给药后的 PK 特征进行研究。

由于灌肠液的保留时间影响美沙拉秦的全身暴露量，因此在这项试验中，受试者保留灌肠液的时间必须能够达到所要求的时间，即 8 h。因此，分别在第-14 天至第-2 天和第-1 天进行灌肠液保留时间为 30 分钟和 8 h 的灌肠液保留测试。受试者将接受安慰剂灌肠液，其外观与颇得斯安®灌肠液相同，但是不含有活性成分美沙拉秦。30 分钟的测试用于排除对直肠给药方式敏感的受试者。8 h 的测试用于排除不能够保留灌肠液 8 h 的受试者。其间潜在受试者应当会意识到灌肠液给药引发的不适，随后可决定是否继续参加这项试验。

还有必要评价 IMP 保留的依从性。使用成人纸尿裤，对 IMP 保留的依从性进行评价。每次 IMP 给药前都让受试者使用新的纸尿裤，并在给药前和给药后 8 h 测量纸尿裤的重量。为了避免蒸发使重量减轻，应在脱掉纸尿裤后 10 分钟内称重。

3.5.2 评价指标的选择

主要指标基于单次和多次给药研究的标准 PK 参数。

3.5.3 设盲

由于本研究为开放性，因而不进行设盲。

3.5.4 研究药物剂量的选择

IMP 为美沙拉秦直肠混悬液，浓度为 1 g/100 mL 醋酸缓冲液。这是一种局部治疗，每天给药一次。在临床应用中不太可能调整 IMP 的剂量。因此，仅选择一个剂量水平，1 g/100 mL。

3.5.5 受试者给药剂量和给药时间的选择

所有受试者均被给予 1 g/100 mL 的固定剂量。IMP 将在入睡前（晚上 9-10 点）给药，与临床应用的时间大致相同。入睡前给药还可促进 IMP 的保留，主要是由于给药后受试者将躺在床上。

IMP 将每天给药一次，连续 7 天，以便能达到稳态（如果在多次给药后能达到稳态），并在此情况下考察 PK。约 10 名受试者将从第 1 天至第 7 天暴露于 IMP。

受试者在 IMP 给药前 2 h 和 IMP 给药后 8 h 禁食禁水。此限制将尽可能减少受试者的活动，即 IMP 给药后如厕，进而尽可能降低灌肠液流出的可能性。

3.5.6 试验人群的选择

选择的试验人群可代表两个性别的中国健康受试者。

IMP 经直肠给药，从直肠扩散到结肠脾曲，并在此处美沙拉秦被吸收进入全身循环。因此将入选胃肠道健康状态的受试者。有临床意义的胃肠道疾病现病史或既往史，胃肠手术史，或者肛门直肠疾病的现病史或既往史（包括瘘管、结构异常、影响保留灌肠液的能力直肠张力疾病）的受试者将被排除。入选的受试者应当是肛门直肠健康的受试者，并在筛选时进行病史、直肠指检和粪便检查予以确认。入选的受试者还应在筛选时通过灌肠液保留测试。

3.5.7 随访流程

由于估计美沙拉秦的全身暴露量在第 9 天可忽略不计，因此未计划随访流程。

4 研究人群的选择

4.1 试验人群

满足所有入选标准并且不满足任何一条排除标准的中国受试者：

4.1.1 入选标准

1. 签署书面知情同意文件（ICD）

2. 年龄为 18-45 岁。受试者签署 ICD 时，必须至少为 18 岁（包括 18 岁生日）且不大于 45 岁（截止 46 岁生日前一天）

3. 体重≥50 kg（男性）或≥45 kg（女性），体重指数（BMI）大于或等于 19.0 但不能超过 24.0 kg/m^2

4. 根据病史、体格检查、12 导联心电图、生命体征及实验室检查结果，受试者身体健康

5. 根据直肠指检和粪便检查结果，受试者的直肠和肛门处于健康状态

6. 根据病史和正常的排便频率（最近 3 个月内每天一次至每周三次），受试者的肠道处于健康状态

7. 第-14 天至第-2 天，能够保留一剂安慰剂灌肠液至少 30 分钟，纸尿裤重量增加不超过 20 g

8. 第-1 天，能够保留一剂安慰剂灌肠液至少 8 h，且纸尿裤重量增加不超过 20 g

9. 在筛选时，人类免疫缺陷病毒抗体（HIV-Ab）、乙肝表面抗原（HBs-Ag）、丙肝病毒抗体（HCV-Ab）和梅毒螺旋体抗体（由 TPPA 测试检测）的病毒学/血清学检查结果均为阴性

10. 在筛选时，尿液药物筛查和酒精呼气测试均为阴性

11. 从签署知情同意文件到试验结束检查后 3 天，受试者同意使用一种屏障避孕方法（例如，避孕套）

12. 不吸烟者或轻度吸烟者（每天不到五支烟，或者相当量）。如果是轻度吸烟者，入住试验中心期间禁止吸烟

13. 对于女性受试者：a）在筛选前 6 个月内，正常月经周期，平均为 28-35 天，b）在筛选前 6 个月内，正常月经期 3-7 天，以及 c）月经结束日在试验第-1 天之前的 7 天内

4.1.2 排除标准

1. 有临床意义的肾、肝、胃肠道、心血管或肌肉骨骼系统的现病史或既往史，或者有临床意义的精神、免疫、内分泌或代谢性疾病的现病史或既往史

2. 过去 5 年内患有癌症，经过充分治疗的皮肤基底细胞癌和鳞状细胞癌除外

3. 重度变态反应或过敏性反应的现病史或既往史，例如对水杨酸类过敏

4. 正在妊娠或哺乳

5. 过去两年内药物滥用史或酗酒史，或者当前药物滥用或酗酒（＞40 g 酒精/天相当于＞1 L 啤酒/天、0.5 L 红酒/天或 6 杯（2 cL）烈酒/天）

6. 既往胃肠手术史

7. 肛门直肠疾病现病史或既往史，包括瘘管、结构异常、以及影响灌肠液保留能力的直肠张力疾病

8. 首次使用美沙拉秦前 2 周内或者药物的 5 个半衰期内（以时间较长者为准），服用过处方药、非处方（OTC）药或中药。治疗细菌或真菌感染的局部用药可被允许，前提是在首次使用 IMP 之前停止使用。

9. 筛选前 12 周内参加过任何临床试验，或筛选前更长时间内参加过临床试验且研究者认为所参加临床试验可能会影响本试验结果

10. 筛选前 12 周内每天饮用大量含有咖啡因的饮料（例如，五杯以上咖啡或者相当量），有在研究期间出现戒断症状的风险，可能混淆安全性评价

11. 第一天给药前 12 周内，献血或者大量失血（≥500 mL）

12. 曾被招募至本研究中并使用了至少一剂 IMP

13. 精神上无行为能力或者语言障碍，难以充分理解或配合

14. 月经期可能与计划给药期（第 1 至 8 天）重合的女性受试者

15. 因其他任何原因，被研究者认为不适合参加本研究

4.2 受试者分配至给药组的方法

4.2.1 招募

被筛选的受试者将获得一个唯一的筛选编号，与受试者编号不同。筛选编号为唯一编号，在受试者首次到试验中心参加筛选时进行分配。接受 IMP 给药的合格受试者将按如下顺序分配受试者编号：01，02，03……。

4.3 限制条件

4.3.1 既往和合并治疗

既往用药定义为从签署 ICD 至给予首剂 IMP 之间任何时间使用的任何药物。伴随用药定义为给予首剂 IMP 后直至完成试验结束检查之间任何时间给予的任何药物。

除了对 AE 进行必需的治疗以外，不允许使用合并用药。研究过程中服用的任何既往或伴随用药将记录在病例报告表（CRF）中，包括剂量、给药方案以及处方的主要原因。

以下药物具有潜在的药物相互作用，因此用作治疗 AE 的合并疗法时需谨慎：

- 肾上腺皮质激素：与肾上腺皮质激素同时使用可能增加胃肠道出血的危险。
- 抗凝剂：与抗凝药物同时使用会增加出血倾向。
- 磺酰脲类口服降糖药：与磺酰脲类口服降糖药同时使用可能增加其降糖作用。
- 螺内酯、呋塞米：与螺内酯和呋塞米同时使用可能降低其利尿作用。
- 丙磺舒、苯磺唑酮：与丙磺舒和苯磺唑酮同时使用可能降低其排尿酸作用。
- 抗代谢药（如甲氨蝶呤、巯嘌呤和硫唑嘌呤）：与抗代谢药（如甲氨蝶呤、巯嘌呤和硫唑嘌呤）同时使用可能增加毒性。
- 利福平：与利福平同时使用可能降低其抗结核病作用。

将使用 ATC 代码标识这些药物。

4.3.2 禁用治疗

除了第 4.3.1 节中提到的治疗和药物之外，不得使用其他治疗或药物。如果受试者使用方案不允许的合并用药（例如，处理 AE 的药物之外的其他任何药物），那么研究者将决定（如果可能要与申办者达成一致后）是否允许受试者继续进行试验。

4.3.3 其他限制条件

为了避免本研究采用的酒精呼气测试出现阳性结果，受试者必须同意在签署 ICD 至试验结束检查访视期间不饮用含酒精饮料。

在签署 ICD 至试验结束检查访视期间，受试者不得饮用大量含咖啡因的饮料（>5 杯咖啡或相当饮料）。

筛选前 12 周内参加过任何临床试验，或筛选前更长时间内参加过临床试验且研究者认为所用药物可能会影响本试验结果，则不能入选本试验。

如果受试者在首次 IMP 给药前 2 周内服用过水杨酸类药物，或者在更长时间内服用但研究者认为可能影响本试验（例如，干扰血浆中美沙拉秦的生物分析），则不能入选本试验。

从首次 IMP 给药前 48 h 开始，一直到试验结束访视完成，受试者应避免进行每周常规活动之外的剧烈身体活动。

仅非吸烟者或轻度吸烟者有资格入选试验。在受试者参加研究的整个过程中，都不允许吸烟。

月经期可能与给药期（第 1 至 8 天）重合的女性受试者不能参加本试验。

如果对水杨酸盐类（例如，乙酰水杨酸和水杨酸钠）过敏，则受试者不能参加本试验。

4.4 退出标准

患者有权在任何时间以任何理由退出试验，不需要说明其决定的合理性。不过，如果受试者提供了退出的主要原因，研究者应当将其记录在受试者的 CRF 中。出于安全原因或者如果受试者不依从研究程序并经判断可能影响本研究的准确性，则研究者有权将受试者撤出。

第 1 次或第 7 次给药后灌肠液流出量多于 20 g 或第 2-6 次给药后灌肠液流出量多于 50 g 的受试者将被认为是对灌肠液给药和保留不依从。使用成人纸尿裤评价 IMP 依从性。第 1 次或第 7 次给药后纸尿裤重量增加超过 20 g，第 2-6 次某一剂给药后纸尿裤重量增加超过 50 g 的受试者将撤出本试验。

对于在给药期间（第 1 至 8 天）开始月经的女性受试者，如果符合以下情况，其应退出本试验：

- 月经在第 7 次 IMP 给药（即第 7 天下午 9-10 点）前开始。
- 月经在第 7 次 IMP 给药后开始，并且第 7 次给药后纸尿裤重量增加 20 g 以上。

在试验期间任何时间被发现怀孕的任何受试者均应立即退出。必须立即向申办者通报任何怀孕情况（见第 8.3 节）。

对于任何停药或退出情况，研究者必须获取方案要求的全部细节，其中包括提前终止日期，这将与主要退出原因一起记录在受试者的 CRF 中。

对于停药或退出研究的受试者，应在可能的情况下评估未报告的不良事件或者事先使用的伴随药物。另外，这些受试者应在试验结束检查时接受全面的后续评估，包括体格检查（身高除外）、生命体征、12 导联心电图、血清妊娠试验（女性）、临床化学、血液学检查和尿液分析。

如果停药或退出的原因是不良事件，则在 CRF 中记录具体事件或者主要的实验室检查异常。由于不良事件而停药或退出的所有受试者将以合适的时间间隔接受随访，目的是评价此不良事件的过程，确保其可逆或稳定。将记录此事件的结局。关于不良事件随访的进一步信息请参见第 8.5 节。

5　给药

5.1　给予的药物

5.1.1　研究药物（IMP）

IMP 为美沙拉秦的直肠混悬液，浓度为 1 g/100 mL 醋酸缓冲液，pH 值在 4.4 和 5.0 之间。使用混悬液而不是溶液被认为是一种优势，因为更多药物物质将与炎症粘膜接触更长时间。这一点非常重要，因为美沙拉秦的治疗作用取决于药物与肠粘膜疾病区域的接触。

受试者将在睡前接受 1 g/100 mL 美沙拉秦灌肠液，每天一次，持续 7 天。IMP 将由经过授权的人员在试验中心进行给药，以确保 IMP 依从性。不拆去药瓶的铝箔包装，将此灌肠液预热至 30~37 ℃。应在安慰剂灌肠液给药至少 22 h 后给予首剂 IMP。

IMP 给药方法：在即刻使用前，将灌肠剂药瓶从铝箔包装中拿出并充分摇匀。顺时针旋转管口一圈以打开密封（管口应处于和旋转前相同的方向）。将手放置于包装中提供的一个塑料袋中。左侧卧位躺下，左腿伸直，右腿弯曲，用于保持平衡并给药。小心的将灌肠剂药瓶的前端放入直肠中，并用手掌充分而稳定的力量将瓶内药物推入。瓶内药物应在最长 30 至 40 秒内完成给药。当瓶子清空后，撤回药瓶前端并保持药瓶处于压缩状态。灌肠剂应保留在肠道内。放松并保持给药姿势 5 至 10 分钟，或直到想排出灌肠剂的感觉消失为止。将塑料袋套在空药瓶外面，丢弃并洗手。更多 IMP 给药信息请参见 IMP 给药介绍（见附件 1 研究药物给药介绍）。

5.1.2 非研究药物（NIMP）

安慰剂灌肠液（100 mL）与 IMP 在外观上相同，但是不含有美沙拉秦。安慰剂灌肠液被用来在筛选时测试受试者保留灌肠液的能力。受试者接受两次安慰剂灌肠液。第一次给药是在第-14 天至第-2 天，要求保留此灌肠液 30 分钟。第二次给药是在第-1 天，要求保留此灌肠液 8 h。安慰剂灌肠液的给药方式与 IMP 相同，也进行预热。

5.2 药品的特征与来源

虽然 IMP 未在中国获得批准，但在其他 71 个国家已上市。IMP 由辉凌制药公司 A/S 提供。IMP 的生产和处理均符合“药品生产质量管理规范”（GMP）的原则。除了贴上试验专用标签外，不会改变其通常的销售状态。

NIMP 安慰剂灌肠液由辉凌制药公司 A/S 提供。其生产和处理均符合 “药品生产质量管理规范（GMP）的原则。

5.2.1 研究药物

商品名：	颇得斯安®。
活性物质：	美沙拉秦（5-氨基水杨酸，5-ASA）。
剂型：	直肠混悬液。
规格：	1 g/100 mL。
供应来源：	辉凌制药公司A/S，丹麦哥本哈根。

5.2.2 非研究药物

商品名：	不适用。
活性物质：	无。
剂型：	直肠混悬液。
规格：	100 mL。
供应来源：	辉凌制药公司A/S，丹麦哥本哈根。

5.3 包装和标签

5.3.1 研究药物

按照 GMP 和国家法规要求，由辉凌制药公司 A/S 的 IMP 部门负责进行研究药物的包装和标签工作。

提供的研究药物将是贴有试验专用标签的药盒。标签还将有一个自粘可撕开部分，可贴在药物清点表上。

5.3.2 非研究药物

按照 GMP 和国家法规要求，由辉凌制药公司 A/S 的 IMP 部门负责进行 NIMP 的包装和标签工作。

提供的 NIMP 将是贴有试验专用标签的药盒。标签还将有一个自粘可撕开部分，可贴在药物清点表上。

5.4 药物贮藏和使用条件

研究者将确保 IMP 和 NIMP 保存在可控制获取的的安全地方，并且保存条件适当。必须用温度监测器定期监测贮藏室并记录温度值。必须向申办方报告任何偏离指定贮藏温度的情况，不得延误，并且必须在收到申办方的进一步指示之后方可使用。

5.4.1 研究药物

研究药物只能发给符合筛选标准的受试者。IMP 应储存在 15~25 ℃。IMP 应在原包装内储存，因为该药物对光线敏感。

5.4.2 非研究药物

NIMP 应储存在 15~25 ℃。NIMP 应在原包装内储存。

5.5 设盲/揭盲

由于本试验为开放研究且无对照组，未采用盲法。

5.6 治疗依从性

5.6.1 药物的发放和清点

由授权的试验人员（如试验护士或药师）分发 IMP 和 NIMP。研究者（或他/她指定的人员，如试验护士）将保留药物分发表，详细记录每例受试者分发与使用研究药物和非研究药物的日期、药量和批号。试验监查员将核实试验期间的药物清点情况。

5.6.2 灌肠液保留评估

IMP 保留依从性被定义为第 1 次和第 7 次给药 8 h 后保留≥80%的灌肠液，第 2 到 6 次每次给药 8 h 后保留≥50%的灌肠液。将采用纸尿裤增量法评估给药依从性。每次给药前，测量新的（未使用过的）成人纸尿裤的重量并进行记录，然后让受试者使用纸尿裤。将在给药后 8 h 再次测量纸尿裤重量并进行记录。应在脱掉纸尿裤后 10 分钟内称重。第 1 次或第 7 次给药后纸尿裤重量增加 20 g 以上，或第 2 到 6 次某一次给药后纸尿裤重量增加 50 g 以上的受试者认为不依从于 IMP 给药，并将退出本试验。

5.7 医疗用品和辅助用品的归还与销毁

在药物清点结束、由监查员核实并由研究者署名后，所有未使用的 IMP 和 NIMP 将按照辉凌公司 IMP 部门的指示及当地的要求销毁或返还后销毁。

6 试验流程

6.1 试验流程图

试验流程见表 1 和图 1。

表 1 试验流程图

评估	筛选		给药期								试验结束检查
研究日	-14至-2	-1	1	2	3	4	5	6	7	8	9
知情同意	X										
入选/排除标准	X	X	X								
人口统计学	X										
病史	X										
体格检查	X										X[a]
直肠指检	X										
粪便检查	X										
生命体征[b]	X	X	X	X	X	X	X	X	X	X	X
12导联心电图	X										X
血清妊娠试验 (女性)[c]	X	X									X
尿液药物筛查	X	X									
酒精呼吸测试	X	X									
血清学/病毒学[c]	X										
临床化学[c]	X	X[d]		X[e]							X
血液学[c]	X	X[d]		X[e]							X
尿液分析[c]	X	X[d]		X[e]							X
灌肠液保留测试 (30分钟)[f]	X										
月经史检查 (女性)[g]	X	X									
灌肠液保留测试 (8 h)[h]		X									
进入试验中心		X									
标准餐		X	X	X	X	X	X	X	X	X	X
禁食禁水[i]		X	X	X	X	X	X	X	X		
肠道排空[j]		X	X	X	X	X	X	X	X		
膀胱排空[k]		X	X	X	X	X	X	X	X		
成人纸尿裤应用和重量测量[l]		X	X	X	X	X	X	X	X		
(N)IMP称重[m]		X	X	X	X	X	X	X	X		
给予IMP[n]			X	X	X	X	X	X	X		
PK血样采集[o]			X	X			X	X	X	X	
尿样采集[p]			X						X	X	
IMP保留[q]			X	X	X	X	X	X	X		
使用便盆[r]		X	X	X	X	X	X	X	X		
排便以及排便时间记录[s]			X	X	X	X	X	X	X	X	
不良事件	X[t]	X	X	X	X	X	X	X	X	X	X
既往/合并用药	X	X	X	X	X	X	X	X	X	X	X
离开研究中心[u]											X

[a] 试验结束检查时不测量体重。
[b] 第-14 天至-2 天、第-1 天、给药期（第 1-8 天）每天、及作为试验结束检查的一部分，在每天上午 8-10 点，休息 5 分钟后，测量生命体征（血压、脉率、腋下温度和呼吸频率）。测量仰卧位血压。
[c] 在当地实验室进行分析。
[d] 如果在第-1 天前 7 天内（即第-8 天至第-2 天）进行了这些检查，那么第-1 天就不再进行。
[e] 第 2 次给药前采集血液和尿液样本。

[f] 必须在 DRE 之前进行灌肠液保留测试（30 分钟，第-14 天至第-2 天进行）。给予受试者一剂安慰剂灌肠液（100 mL）。将安慰剂灌肠液预热至 30~37℃，然后给药，给药方式与 IMP 相同。受试者使用称重过的纸尿裤。给药 30 分钟后再次称量纸尿裤的重量。应在脱掉纸尿裤后 10 分钟内称重。合格的受试者应保留灌肠液至少 30 分钟，纸尿裤的重量增加不超过 20 g。

[g] 第-14 天至第-2 天和第-1 天检查月经史（女性）。合格女性受试者的月经期不可能与给药期重合。

[h] 第-1 天，进行灌肠液保留测试（8 h）。第 1 剂 IMP 给药前至少 22 h 给予受试者安慰剂灌肠液（100 mL）。所有与 IMP 依从性改善和评价有关的步骤均与 IMP 给药相同，包括禁食和禁水 [i]，肠道排空 [j]，膀胱排空 [k]，成人纸尿裤应用和重量测量 [l]，（N）IMP 称重 [m]，（N）IMP 保留 [p]，使用便盆 [q]，排便和排便时间记录 [r]。合格受试者应保留灌肠液至少 8 h，纸尿裤重量增加小于或等于 20 g。

[i] 受试者在给药前 2 h 到给药后至少 8 h 禁食禁水。

[j] 第-1 天在第-1 天 NIMP 给药前、第 1 次和第 7 次 IMP 给药前约 2 h 受试者使用温水灌肠，以促进肠道排空。在第 2 次至第 6 次 IMP 给药前约 2 h，会要求受试者如厕，尽量排空肠道，但不使用温水灌肠。

[k] NIMP 给药前（第-1 天）和每次 IMP 用药前（第 1 至 7 天）约 10 分钟都要求如厕以排空膀胱。

[l] 使用成人纸尿裤，对（N）IMP 保留的依从性进行评价。测量并记录新的（未使用过的）纸尿裤的重量，之后让受试者使用此纸尿裤。给药 8 h 后，再次称取纸尿裤的重量。应在脱掉纸尿裤后 10 分钟内称重。

[m] 将铝箔包装的（N）IMP 预热至 30~37 ℃，擦干后，拆掉铝箔包装并称重。给予（N）IMP 后，再次称量使用过的（N）IMP 的重量。记录未使用和已使用（N）IMP 的重量。

[n] 在第 1-7 天晚上 9-10 点左右给予 IMP。

[o] 在第 1 次、第 5 次和第 7 次给药后 24 h 内重复采集用于美沙拉秦分析的血样。第 1 次、第 5 次和第 7 次给药对应的采样时间为：给药前即刻，及给药后 0.5、1、2、3、4、5、6、7、8、10、12、14、16、20、24 h（下次给药前）。

[p] 对于药物排泄测定，在第 7 次给药后（第 7 次给药后 0-12 h 和 12-24 h 内）采集尿样。在第 1 次给药前采集空白尿样。

[q] 应当保留 IMP 到给药后至少 8 h。

[r] 给药后 8 h 内需排尿的受试者将在床上使用便盆。

[s] 测量纸尿裤重量后会要求受试者在给药后 8-10 h 如厕，并记录排便时间（记录出、入为厕所的时间）。

[t] 签署 ICD 后开始收集 AE。

[u] 如果试验结束检查的结果被认为是“正常”或“异常但无临床意义”，受试者才可离开试验中心。如果此时任何指标被认为是“异常且有临床意义”，受试者离开试验中心前由研究者决定采取其他措施。

图 1 试验示意图

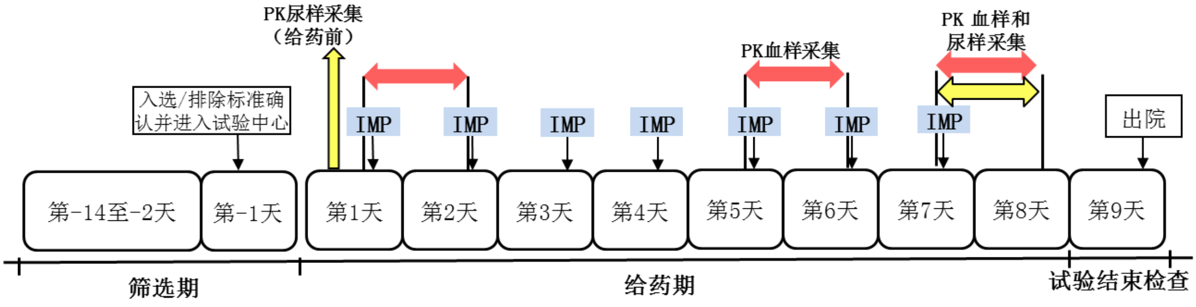

注：本研究没有第 0 天。

6.2 筛选

第-14 天至第-2 天

所有受试者将提供知情同意并接受入选检查，评估其健康状态。此检查将在计划的第 1 天首次 IMP 给药前的第-14 天至第-2 天之间实施。

筛选检查将包括以下各项：

- 第 7.2.7 节详述的病史；
- 第 7.1.8 节详述的体格检查；
- 第 7.2.6 节人口统计学信息的收集；
- 第 7.1.2 节详述的生命体征；
- 第 7.1.3 节详述的 12 导联心电图；
- 第 7.1.5 节详述的临床化学检查；
- 第 7.1.6 节详述的血液学检查；
- 第 7.1.7 节详述的尿液分析；
- 第 7.2.5 节详述的尿液药物筛查和酒精呼吸测试
- 第7.2.3节详述的血清学和病毒学检查；
- 第 7.2.1 节详述的直肠指检；
- 第 7.2.2 节详述的粪便检查；
- 第 7.2.8 节详述的既往用药；
- 第 7.2.4 节详述的血清妊娠试验（女性）；
- 第 7.2.9 节详述的灌肠液保留测试（30 分钟）；
- 第 7.1.4 节详述的治疗前 AE 评估；
- 过去 6 个月的月经史（女性），包括月经周期的平均天数、平均月经期天数和上次月经的开始日期。

第-1 天

受试者将在第-1 天入住试验中心。在第-1 天，受试者将接受入选/排除标准的简要核对，并进行入选/排除标准检查，包括以下各项：

- 第 7.1.2 节详述的生命体征；
- 第 7.2.4 节详述的血清妊娠试验（女性）；
- 第 7.2.5 节详述的尿液药物筛查和酒精呼气测试；
- 第 7.1.5 节详述的临床化学检查；
- 第 7.1.6 节详述的血液学检查；
- 第 7.1.7 节详述的尿液分析；
- 第 7.2.8 节详述的既往用药；
- 第 7.2.10 节详述的灌肠液保留测试（8 h）；
- 第 7.1.4 节详述的治疗前 AE 评估；
- 核对女性受试者的上个月经结束日期，此日期应在第 1 天前的 7 天内（第-7 天至第-1 天）。

如果第-1 天前 7 天内（即，第-8 天至第-2 天）进行了临床化学、血液学、尿液分析，则不进行这些检查。

合格受试者将被视为筛选成功并可以接受 IMP 给药。

6.3 给药期

给药期为第 1 天至第 8 天。

在第 1 天第 1 次 IMP 给药前评估既往用药和治疗前 AE。

图 2 与 IMP 给药依从性提高和评估相关的程序

每次 IMP 给药前后均实施以下程序。

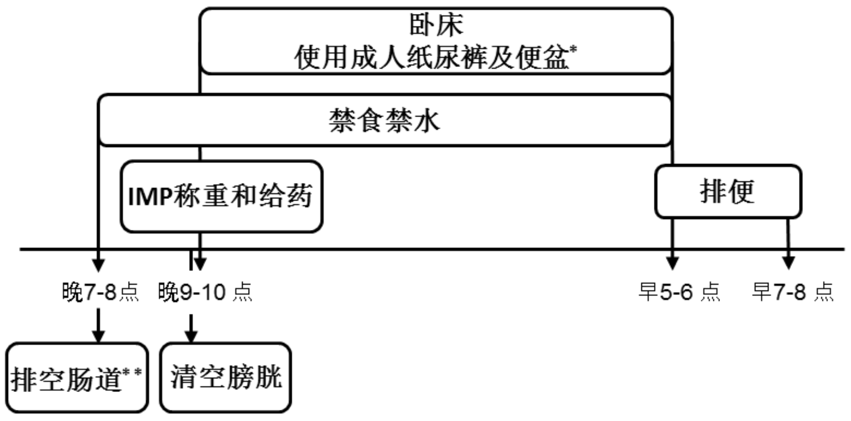

*在需要排尿时使用便盆。

**第 1 次和第 7 次给药前将给予温水灌肠排空肠道。

受试者将在第 1-7 天晚上 9-10 点接受每天一次的颇得斯安®灌肠液（1 g/100 mL）直肠给药，并按要求保留此灌肠液至少为 IMP 给药后 8 h。在每次 IMP 给药前后都要实施以下程序（图 2），以提高并评价 IMP 保留的依从性（除非有特殊说明）：

- 给药前 2 h（即晚上 7-8 点），受试者禁食禁水，一直到给药后 8 h（即第二天上午 5-6 点）。
- 每次用药前约 2 h，会要求受试者如厕，尽可能排空肠道。在第 1 次和第 7 次给药前，将使用温水灌肠以便排空肠道，但第 2 次至第 6 次给药前不使用温水灌肠。
- 每次用药前约 10 分钟都要求如厕以排空膀胱。
- 将带铝箔包装的 IMP 预热至 30~37 ℃，擦干后，拆掉铝箔包装并称重。给予 IMP 后，再次称量使用过的 IMP 的重量。记录未使用和已使用 IMP 的重量。
- 使用成人纸尿裤对 IMP 保留的依从性进行评价。测量并记录新（未使用过的）纸尿裤的重量，之后让受试者使用此纸尿裤。给药 8 h 后，再次称取纸尿裤的重量。应在脱掉纸尿裤后 10 分钟内称重。如果第 1 剂或第 7 剂给药后纸尿裤重量增加 20 g 以上，第 2-6 剂某一剂给药后纸尿裤重量增加 50 g 以上，则出现该情况的受试者将退出本试验。
- 给药后 8 h 内需排尿的受试者将在床上使用便盆。
- 测量纸尿裤重量后，要求受试者在给药后 8-10 h 时间段内如厕，并记录排便时间（记录为进、出厕所的时间）。

用于美沙拉秦分析的血样分别在第 1 次、第 5 次和第 7 次给药后 24 h 内收集。3 次给药对应的采样时间为：给药前即刻和给药后 0.5、1、2、3、4、5、6、7、8、10、12、14、16、20、24 h（下次给药前）。每个时间点采血量约 4 mL。

采集尿液用于美沙拉秦及其主要代谢物 N-乙酰基美沙拉秦的分析。在第 1 次给药前采集 5~10 mL 空白尿液，第 7 次给药后分别于 0-12 h 和 12-24 h 时间段内采集所有排出的尿液。

从第 1 天至第 8 天每天测量生命体征，生命体征描述见第 7.1.2 节。

除了生命体征，第 2 天还将进行以下检查：

- 第 7.1.5 节详述的临床化学检查；
- 第 7.1.6 节详述的血液学检查；
- 第 7.1.7 节详述的尿液分析；

受试者将在试验中心与经过培训的工作人员面谈，获取关于可能的不良事件和伴随用药的信息。如果报告有不良事件，研究者可决定进行/重复一次临床检查和/或实验室检查。

6.4 试验结束检查

试验结束检查将在第 9 天进行。此次检查包括以下各项：

- 体格检查（身高除外），如第 7.1.8 节所述；
- 第 7.1.2 节详述的生命体征；
- 第 7.1.3 节详述的 12 导联心电图；
- 第 7.1.5 节详述的临床化学检查；
- 第 7.1.6 节详述的血液学检查；
- 第 7.1.7 节详述的尿液分析；
- 第 7.2.4 节详述的血清妊娠试验（女性）；
- 不良事件和伴随用药的评估。

如果试验结束检查的结果被认为是“正常”或“异常但无临床意义”，受试者才可离开试验中心。如果此时任何指标被认为是“异常且有临床意义”，受试者离开试验中心前由研究者决定进行其他治疗。

正在进行的不良事件将根据第 8 节详述的各项程序进行随访。对有临床意义的实验室检查值进行随访，直到其恢复到参考值范围内，或者直到受试者的情况稳定。

7 试验评估

本试验中的大部分评估是基于实验室检测。实验室手册将描述与血浆中美沙拉秦测定相关的程序，包括采样和运输详情、联系信息、贮存条件等。该实验室手册将在试验开始前提供给试验中心。所有其他在本地实验室检测的程序将根据本地常规进行。

PK 血液采样和其他实验室评估的时间点在表 1 试验流程图中展示。

7.1 评价指标相关的评估

7.1.1 药代动力学评估

主要 PK 指标基于美沙拉秦血药浓度的测定。美沙拉秦血药浓度的生物分析将由一家有资质的生物分析实验室采用经确证的 LC-MS/MS 方法进行。

第 1 次、第 5 次和第 7 次给药后 24 h 内重复收集用于美沙拉秦分析的血样。三次给药对应的采样时间为：给药前即刻和给药后 0.5、1、2、3、4、5、6、7、8、10、12、14、16、20、24 h（下次给药前）。每个时间点将采血约 4 mL。

次要 PK 指标之一，尿液排泄中美沙拉秦和 N-乙酰基美沙拉秦的总蓄积量，根据尿液中美沙拉秦和 N-乙酰基美沙拉秦浓度和尿量来确定。第 7 次给药后 0-12 h 和 12-24 h 的总尿量由研究中心使用适合的量筒测量。尿液中美沙拉秦和 N-乙酰基美沙拉秦浓度由一家有资质的生物分析实验室采用经过验证的 LC-MS/MS 法进行同时测定。

第 1 次给药前采集 5~10 mL 空白尿液。第 7 次给药后分别于 0-12 h 和 12-24 h 时间段内采集所有排出的尿液。

实际采样时间将记录在 CRF 中，并用于 PK 参数计算。

7.1.2 生命体征

生命体征包括仰卧位血压、脉搏、腋下温度和呼吸频率。将在第-14 天至第-2 天、第-1 天、给药期（第 1 至 8 天）每一天以及第 9 天试验结束检查时进行生命体征测量。

每次测量仰卧位收缩压和舒张压将使用同一只手臂，并且在受试者仰卧位休息至少 5 分钟后测量。将使用标准设备进行所有记录，自动化或人工测量均可接受，但是研究者应继续使用与第一次测量时所使用的方法相同的方法进行个体受试者的所有评估。与基线相比，具有临床意义的异常值将报告为不良事件。

7.1.3 心电图

将在筛选时（第-14 天至第-2 天）和第 9 天试验结束检查时记录 12 导联心电图检查结果。

ECG 记录将收集至少 4 个 QRS 波群即 3 个可评估 RR 间期。研究者或指定人员将评估 ECG 的临床意义为“正常”、“异常但是没有临床意义”或者“异常且有临床意义”。将评估发生的去极化或复极化异常、心律失常或其他异常，并且将对与研究药物用药前相比的任何变化给出意见。与基线相比，异常具有临床意义的事件将报告为不良事件。

7.1.4　不良事件

将从受试者签署 ICD 直到试验结束检查完成的时间内收集关于不良事件的信息。对于每例不良事件，均在“不良事件表”中记录以下参数：事件描述、发作日期、强度、与 IMP 的因果关系、对 IMP 采取的措施、采取的其他措施、不良事件严重性、结局日期以及结局。

关于不良事件和严重不良事件的定义与报告的进一步信息，请参见第 8 节。

7.1.5　临床化学

将在第-14 天至第-2 天、第-1 天、给药期（第 1 至 8 天）的第 2 天以及第 9 天试验结束检查时由当地实验室进行表 3 列出的临床化学参数的检测。如果在第-1 天前 7 天内（即，第-8 天至第-2 天）进行了这些检查，那么第-1 天就不再进行。所有数据都将由研究者输入 CRF。

筛选过程中进行的、第-1 天再次进行（如果适用）的临床化学结果将被用来确定受试者是否有资格参加本试验。第-1 天的临床化学数据将用作分析安全性研究结果的基线；不过，如果第-1 天不进行检查，则将第-14 天至第-2 天获得的数据用作基线。如研究者认为筛选时或者第-1 天的实验室异常检查值有临床意义，受试者不应在第 1 天接受给药。

第 2 天和第 9 天将进行进一步的临床化学检查。

研究者将检查所有临床化学结果，并在当地实验室参照范围基础上评估结果是高、低、还是正常。另外，研究者将评估每个结果的临床意义，并记录为“正常”、“异常但无临床意义”或“异常且有临床意义”。实验室报告将由研究者签署并注明日期。与基线相比，实验室检查值中“异常且有临床意义”的变化应报告为不良事件（见第 8 节）。

表 3 安全性实验室参数–临床化学

白蛋白
碱性磷酸酶
丙氨酸转氨酶
天冬氨酸转氨酶
血氯
血钙
总胆固醇
血肌酐
血肌酐清除率*
γ-谷氨酰转移酶
血钾
血钠
总胆红素
血尿素氮
尿酸

*血肌酐清除率由 Cockcroft-Gualt 计算得出。

7.1.6 血液学

将在筛选时（第-14 天至第-2 天）、第-1 天、给药期（第 1 至 8 天）的第 2 天以及第 9 天试验结束检查时由当地实验室进行表 4 列出的血液学参数的检测。如果在第-1 天前 7 天内（即，第-8 天至第-2 天）进行了这些检查，那么第-1 天就不再进行。所有数据都将由研究者输入 CRF。

筛选过程中进行的、第-1 天再次进行（如果适用）的血液学结果将被用来确定受试者是否有资格参加本试验。第-1 天的血液学数据将用作分析安全性研究结果的基线；不过，如果第-1 天不进行检查，则将第-14 天至第-2 天获得的数据用作基线。如研究者认为筛选时（第-14 天至第-2 天）或者第-1 天的实验室异常检查值有临床意义，受试者不应在第 1 天接受给药。

第 2 天和第 9 天将进行进一步的血液学检查。

研究者将检查所有血液学结果，并在当地实验室参考范围基础上评估结果是高、低、还是正常。另外，研究者将评估每个结果的临床意义，并记录为“正常”、“异常但无临床意义”或“异常且有临床意义”。实验室报告将由研究者签署并注明日期。与基线相比，实验室检查值中“异常且有临床意义”的变化应报告为不良事件（见第 8 节）。

表 4 安全性实验室参数–血液学

红细胞压积
血红蛋白
红细胞平均血红蛋白浓度
平均细胞压积
血小板计数
红细胞计数
白细胞计数
白细胞计数分类：
中性粒细胞%
嗜酸性粒细胞%
嗜碱性粒细胞%
淋巴细胞%
单核细胞%

7.1.7 尿液分析

将在第-14 天至第-2 天、第-1 天、在给药期（第 1 至 8 天）的第 2 天以及第 9 天试验结束检查时由当地实验室进行表 5 列出的尿液分析参数的检测。如果在第-1 天前 7 天内（即，第-8 天至第-2 天）进行了这些检查，那么第-1 天就不再进行。尿液分析评估应使用中段尿样本进行。所有数据都将由研究者输入 CRF。

第-14 天至第-2 天进行的、第-1 天再次进行（如果适用）的尿液分析结果将被用来确定受试者是否有资格参加本试验。第-1 天的尿液分析数据将用作分析安全性研究结果的基线；不过，如果第-1 天不进行检查，则将筛选过程中获得的数据用作基线。如研究者认为第-14 天至第-2 天或者第-1 天的实验室异常检查值有临床意义，受试者不应在第 1 天接受给药。

第 2 天和第 9 天将进行进一步的尿液分析检查。

研究者将检查所有尿液分析结果，并在当地实验室参照范围基础上评估结果是高、低、还是正常。另外，研究者将评估每个结果的临床意义，并记录为“正常”、“异常但无临床意义”或“异常且有临床意义”。实验室报告将由研究者签署并注明日期。与基线相比，实验室检查值中“异常且有临床意义”的变化应报告为不良事件（见第 8 节）。

表 5 安全性实验室参数–尿液分析

尿潜血
尿葡萄糖
尿酮类
尿白细胞计数
pH 值
尿蛋白

7.1.8 体格检查

将在第-14 天第-2 天进行一次全面的体格检查，以便根据入选和排除标准确保合格性。第-14 天至第-2 天将测量身高和体重以计算体重指数。受试者离开试验中心前，还要在试验结束检查时进行体格检查；不过，身高仅在筛选访视时测量。

体格检查包括一般情况、中枢和周围神经系统、头颈部（包括耳、眼、鼻、口和喉）、呼吸系统、心血管系统、胃肠系统、淋巴系统、泌尿系统、生殖系统（包括乳房）、肌肉骨骼系统以及皮肤。

与基线相比，任何具有显著临床意义的异常记录为不良事件。

7.2 其他评估

7.2.1 直肠指检

直肠指检（DRE）是的目的是检查直肠下段和邻近器官或组织是否有异常。筛选过程中（第-14 天至第-2 天）将进行直肠指检，确保入选受试者的直肠和肛门处于健康状态。入选受试者的直肠指检结果应显示其具有健康的直肠和肛门状态。直肠指检结果显示有其他异常的受试者，例如前列腺异常，可根据第 4.1 节的入选/排除标准由研究者决定。

7.2.2 粪便检查

仅在筛选时（第-14 天至第-2 天）进行粪便检查。以下结果中有任何一项呈阳性的受试者不能入选本试验：

- 粪便隐血;
- 红细胞；
- 粘液；
- 虫卵；

- 原虫。

7.2.3 血清学和病毒学

血清学和病毒学评估（在血清中测量）仅在筛选时进行。以下任何一项检测为阳性的受试者不适于参加本试验：

- HIV-AB；
- HBs-AG；
- HCV-AB；
- TPPA。

7.2.4 血清妊娠试验（女性）

血清 β-人绒毛膜促性腺激素（βhCG）将在第-14 天至第-2 天、第-1 天和第 9 天采用经过确证的标准方法进行测定。所有血清妊娠试验（女性）的结果都将由研究者输入 CRF。

妊娠试验阳性/在研究期间任何时间怀孕的女性受试者不能继续参加研究，应立即退出（见第 4.4 节），同时向申办者报告此妊娠事件，如第 8.3 节所述。

7.2.5 尿液药物筛查和酒精呼气测试

研究期间的任何时间均不允许使用违禁药品。从签署 ICD 到试验结束检查，都不允许饮酒（见第 4.3.3 节）。

将第-1 天进行药物和酒精筛查评估。酒精阳性（呼气测试）或下述任何药物检测结果阳性的受试者，不能入组：

- 安非他命
- 巴比妥类
- 苯二氮卓类
- 大麻素类（大麻）
- 三环类抗抑郁药（去甲替林）
- 甲烯二氧甲苯丙胺（迷幻药）
- 美沙酮
- 可卡因
- 吗啡/鸦片
- 苯环己哌啶

应使用新鲜中段尿液进行药物筛查。

7.2.6 人口统计数据和基线数据

将在筛选期间收集人口统计数据和基线数据，包括性别和出生日期。这些数据将输入到CRF中。

7.2.7 病史

签署ICD前出现的具有临床意义的既往和伴随疾病信息（或者体征和症状，如果不能确定诊断），将作为筛选的病史记录在CRF中。有特定既往或目前疾病的受试者不能参加研究（参见第4.1节的入选/排除标准）。

7.2.8 既往和伴随用药

既往或伴随用药的定义在第4.3.1节中。

研究期间从最初筛选到第9天试验结束检查，服用的所有既往用药和任何伴随用药，将由研究者使用商品名（可用时，否则使用通用名）记录到CRF中。将记录的其他信息，包括服药的主要原因、用药途径和治疗时间。如果伴随用药是中药，应记录为“中药”，同时记录商品名和主要药材名称（在已知的情况下）。

7.2.9 灌肠液保留测试（30分钟）

筛选时第-14天至第-2天进行灌肠液保留测试（30分钟）。必须在直肠指检之前进行测试，原因是直肠指检可能影响肛门括约肌的功能，进而可能影响灌肠液保留测试。

给予受试者安慰剂灌肠液（100 mL）。将安慰剂灌肠液预热至30~37℃，然后给药，给药方式与IMP相同（附件1）。要求受试者保留灌肠液至少30分钟。

将使用纸尿裤评价安慰剂灌肠液保留的依从性。测量并记录新的（未使用过的）纸尿裤的重量，之后让受试者使用此纸尿裤。给予安慰剂灌肠液30分钟后，再次称取纸尿裤的重量。应在脱掉纸尿裤后10分钟内称重。纸尿裤重量增加20 g以上的受试者将不能入组。

7.2.10 灌肠液保留测试（8 h）

在第-1天使用安慰剂灌肠液（100 mL）进行灌肠液保留测试（8 h）。安慰剂灌肠液给药必须在第1次IMP给药前至少22 h进行。IMP依从性提高和评价使用的所有程序也将用于本次测试，包括以下内容：

- 给药前 2 h，受试者禁食禁水，一直到给药后 8 h。
- 用药前约 2 h，用温水灌肠排空肠道。
- 用药前约 10 分钟要求如厕以排空膀胱。
- 将带铝箔包装的 NIMP 预热至 30~37 ℃，擦干后，拆掉铝箔包装并称重。给予 NIMP 后，再次称量使用过的 NIMP 的重量。记录未使用和已使用 NIMP 的重量。
- 使用成人纸尿裤对 NIMP 保留的依从性进行评价。测量并记录新（未使用过的）纸尿裤的重量，之后让受试者使用此纸尿裤。给药 8 h 后，再次称取纸尿裤的重量。应在脱掉纸尿裤后 10 分钟内称重。
- 给药后 8 h 内需排尿的受试者将在床上使用便盆。
- 测量纸尿裤重量后，要求受试者在给药后 8-10 h 时间段内如厕，并记录排便时间（记录为出、入厕所的时间）。

纸尿裤重量增加超过 20 g 的受试者不允许给予 IMP。

7.3 药物浓度测定

人血浆中美沙拉秦以及人尿液中美沙拉秦和 N-乙酰基美沙拉秦的测定将由中国的一家具有资质的生物分析实验室进行。采用 LC-MS/MS 法进行测定，并且最好采用稳定同位素标记的内标。方法学验证和样本分析将按照相关指南[16]进行。

7.4 生物样本处理

血浆和尿液样本生物学分析：

每个样本粘贴标签，标签上的内容有试验编号、受试者编号、采样日期和给药相关的采样时间。在实验室手册中详细描述样本处理信息，包括装运信息。生物分析样本将储存至临床试验报告完成后 2 年。如果受试者撤出同意，并要求将其生物样本销毁，则立即销毁样本。样本销毁必须由申办者批准并具有销毁记录。销毁确认必须发送至申办者。

其他生物样本：

除了用于分析美沙拉秦和 N-乙酰基美沙拉秦生物分析样本，将在本地实验室进行所有其他实验室为基础的评估。未使用的样本将根据本地政策在完成检测后销毁。

不论在哪里进行分析，除了本方案中规定的分析，不会对收集的生物学样本进行另外的分析，除非从伦理委员会（EC）、监管机构（CFDA）和/或尽可能事先从试验受试者获得另外的批准/同意。如果经适当努力，不能联系到受试者获得同意，但是已经获得 EC 和/或 CFDA 批准，可以进行附加检测。

8 不良事件

8.1 不良事件定义

不良事件是指参与临床试验的受试者发生的任何不良医学事件。包括：

- 任何与研究药物使用有时间关系的不利或非预期的体征、症状或疾病，不论是否认为由研究药物引起。
- 常规观察到的不良事件以及由研究药物药理作用可预见的不良事件。
- 研究者认为具有临床意义的实验室异常、生命体征或体格检查发现（注意：筛选期间进行的评估和检查所发现的异常不是不良事件，而是作为病史记录）
- 意外伤害，调整用药（药物和/或剂量）的原因，任何导致进行医疗、护理或药理咨询的原因，导致住院或手术的原因。
- 用药过量和用药差错，无论是否有临床影响。

治疗前不良事件：

治疗前不良事件是指签署知情同意后到开始使用研究药物期间发生的不良事件，或者是在签署知情同意后到开始使用研究药物期间预先存在的医学情况加重。

治疗中出现的不良事件：

治疗中出现的不良事件是指在开始使用研究药物后到完成第 9 天的试验结束检查期间发生的不良事件，或者是治疗前不良事件或预先存在的医学情况加重。

8.2 不良事件的收集和记录

8.2.1 不良事件的收集

从获得签署知情同意书开始直至末次访视，研究者必须监测试验期间受试者的健康状况。

AE 的来源包括：

- 受试者对他/她健康状况问题的回答（每次访视提出标准的非引导性问题例如“自上次访视以来您感觉如何？”）。
- 受试者自发报告的症状
- 研究者评估认为其发现是具有临床意义的变化或异常的研究或检查
- 研究者获知的与受试者健康有关的其他信息（例如：住院）

8.2.2 不良事件的记录

研究者必须在每位受试者的 CRF 中提供的不良事件表中记录所有不良事件，其中应包括下述信息：

- 不良事件名称
- 开始的日期和时间（如果不适用，可以省略时间）
- 严重程度
- 与研究药物的关系
- 对研究药物采取的措施
- 采取的其他措施
- 转归的日期和时间（如果不适用，可以省略时间）
- 转归
- 严重程度

以下章节详细描述了不良事件记录中的每个项目。

<u>不良事件的名称</u>

如果可以，不良事件应该按照确切的诊断来记录。如果不能记录为确切的诊断，应分别记录单独的体征和症状。每个诊断/症状都应分别记录。

如果同一种不良事件在受试者身上发生一次以上，且受试者在不良事件之间康复过，则此不良事件应按照发生次数分别记录。如果不良事件发生期间其严重程度发生变化，记录此不良事件时应记录最差的情况，即记录最严重的程度和持续最长的时间[a]。

必须注意：采取的措施不是不良事件，采取措施的原因才是不良事件。住院不是不良事件，住院的原因才是不良事件。死亡不是不良事件，导致死亡的原因才是不良事件（不明原因的猝死例外，应记录为不良事件）。

开始的日期和时间

不良事件的开始时间是第一次观察到第一个体征或症状的日期。如果不良事件是具有临床意义的异常实验室检测或检查结果，开始时间即是采样日期或检查日期。

严重程度

必须利用以下 3 级量表对不良事件的强度进行分类：

轻度： 发现体征或症状，但是没有妨碍日常活动

中度： 事件足以影响日常活动（令人烦恼）

重度： 不能工作或进行日常活动（不可接受）

与研究药物的因果关系

不良事件与研究药物因果关系的可能性必须归类为以下一种：

可能有关： 有证据或论据表明研究药物与不良事件之间存在因果关系。不良事件可能是研究药物部分药理作用的结果或可能为非预期作用的结果。

举例：

- 不良事件不常见，但是已知与研究药物暴露有较大关联
- 不良事件一般与研究药物暴露无关，但是事件的出现与表明因果关

[a]例外：如果不良事件在研究药物首次给药前发生（即治疗前不良事件），且强度改变，则该不良事件必须记录为两个单独事件。最初的不良事件结局应记录为“尚未恢复”，并且结局的日期和时间应为强度发生变化时的时间。第二个不良事件的开始日期和时间应记录为强度发生变化的时间。

系的其他因素相关，例如明确的时间关系或再次给药时事件再次出现

可能无关 没有合理的证据或论据表明研究药物与不良事件之间存在因果关系。

举例：

- 为已知的潜在疾病或研究状况的后果。
- 不良事件在试验人群中常见，并且预期试验期间将会经常出现，不论是否暴露于研究药物。
- 与操作相关的不良事件。

对研究药物采取的措施

为处理不良事件而对研究药物采取的措施必须归类为以下一种：

- 继续/无变化（保持用药计划或不采取措施）
- 中止/退出

采取的其他措施

需要给予治疗的不良事件必须采用公认的医疗标准进行治疗，保护受试者的健康和利益。相应的心肺复苏设备和药品必须可以使用，确保紧急情况下提供可能的最佳治疗。

如果使用药物治疗不良事件，应在合并用药记录中记录使用的药物。

结果的日期和时间

受试者康复或死亡的日期和时间。

转归

不良事件的转归必须归类为以下一种：

- 康复但有后遗症（导致持续性或明显的残疾/功能障碍）
- 康复没有后遗症（完全康复或疾病恢复到开始治疗前观察到的水平）
- 症状持续/未康复
- 死亡

8.3 妊娠和妊娠转归

如确定妊娠，则应立即停止试验药物，怀孕受试者应退出试验，并且在 3 天内采用妊娠报告表，通知辉凌制药公司 A/S 的全球药物警戒部门（GPV）。注意，妊娠本身并不是严重不良事件。母亲和胎儿必须至少随访到分娩和婴儿出生后一个月。一般来说，随访将包括怀孕事件的进程，时间，怀孕的结果和新生儿健康状况。如果分娩的结果是异常的（例如出生缺陷、先天畸形、死胎、新生儿死亡等），必须按照第 8.4.2 节中描述的程序，将其作为严重不良事件（SAE）报告给辉凌公司和 CFDA。研究者和/或申办方认为与研究药物有关的任何异常妊娠结果都将作为加速报告处理。

关于男性受试者，如果父亲暴露于研究药物后，研究药物通过精子传递，胎儿也可能暴露于研究药物且如果怀孕导致异常结果（例如出生缺陷、先天畸形、死胎、新生儿死亡等），必须按照第 8.4.2 节中描述的程序，将其作为 SAE 报告给辉凌制药公司 A/S 的 GPV 和 CFDA。研究者和/或申办方认为与研究药物有关的任何妊娠异常结果都将作为加速报告处理。

8.4 严重不良事件

8.4.1 严重不良事件的定义

临床试验期间的 SAE

如果出现下述情况则事件定义为严重不良事件	*指导原则*
导致**死亡**	必须全面记录并报告导致致命性结果的任何事件，包括治疗结束后 4 周内出现的死亡，不论事件与研究药物是否存在因果关系。参加研究的受试者的死亡本身不属于不良事件，而是不良事件的结果。
**危及生命**	危及生命是指事件出现时，受试者面临死亡的直接风险，不是指如果情况更加严重可能导致死亡的事件。

需要**住院**或导致原有住院时间**延长**	指受试者由于事件的出现导致住院或原有住院时间延长。住院时间至少为 24 小时。在医院留院观察、在急诊室观察或在门诊治疗均不属于住院。但是，必须给予医学判断，并且有疑问时，应视为严重病例（即怀疑病例符合重要医学事件的标准）。为满足护理需要或社会管理需要的住院不属于 SAE。如果受试者参加试验前已经患有疾病并且试验期间疾病没有加重，那么执行的试验前已经计划的住院治疗和/或手术治疗不属于不良事件。
导致持续的或明显的**伤残/功能障碍**	残疾/功能障碍是指受试者进行正常生活的能力严重受损。若有怀疑，应由研究者做出医学判断。
**先天畸形/出生缺陷**	指在受试者接受研究药物治疗期间孕育的后代中观察到的先天畸形/出生缺陷。
**重要医学事件/其他**	重要医学事件是指可能不会立即危及生命，或导致死亡或住院的事件，但是可能会危害受试者，或需要给予治疗才能防止出现以上定义中列出的结果之一。重要医学事件的例子包括：表明重要危害的不良事件，禁忌症或危险因素，出现恶性肿瘤，或出现药物依赖性或药物滥用。应依据医学和科学的判断，来决定是否为重要医学事件时。<br>重要医学事件包括可疑的传染原通过医药产品的传播。任何致病性或非致病性生物体病毒或感染颗粒（例如：朊病毒蛋白传播的传染性海绵状脑病）均被认为是传染原。可以从暴露于药品的感染受试者的临床症状或实验室发现，推测传染原的传播。

试验后随访期间的严重不良事件

如果试验结束后研究者发现了严重不良事件且评估认为严重不良事件与研究药物有关，则必须按照第 8.4.2 节中规定的程序将该病例报告至辉凌公司；至于试验结束后多长时间发生了严重不良事件则不在考虑范围内。此类报告将考虑进行加速报告且由辉凌公司进行管理。

8.4.2 严重不良事件的收集、记录和报告

研究者报告严重不良事件

当发生 SAE 时，研究者应完整并及时地记录所有相关信息，记录应尽可能详细。研究者应在得知 SAE 发生的 24 h 内向 CFDA 和辉凌提交严重不良事件报告表。

研究者应提交的严重不良事件报告表如下：

- 向主管部门（CFDA）报告（传真至 010 88363228）
  - 研究者在得知事件发生（第 0 天）的 24 h（第 1 天）内，通过 CFDA 发放的严重不良事件报告表以中文版的形式报告
- 向辉凌报告（即向 Ferring International Pharma-Science Centre(China) Co. Ltd 的当地安全专员（LSO）报告）（电子邮件：SafetyMailboxChinaBeijing_BDC@ferring.com 和/或传真至 010 85295335）
  - 在研究者得知事件发生（第 0 天）的 24 h（第 1 天）内，通过 CFDA 发放的严重不良事件报告表以中文版的形式报告
  - 在研究者得知事件发生（第 0 天）的 24 h（第 1 天）内，通过 CFDA 发放的严重不良事件报告表以英文版的形式报告

将对所有报告的 SAE 表进行分析，并将国际医学科学组织理事会（CIOMS）表格发送给研究者。研究者应在 15 日内向伦理委员会提交严重不良事件及该 CIOMS 表格。

关于报告的 SAE 病例，CFDA SAE 报告表和辉凌批准的严重不良事件报告表应包括以下信息：严重度、与研究药物的关系、针对研究药物采取的措施的类型及其当前状态。

如果发生与辉凌 NIMP 相关的 SAE，则作为自发报告处理。所有与辉凌 NIMP 相关的 SAE 必须报告。

辉凌加速报告

辉凌将在规定的时限内向相关各方报告研究者或辉凌判断为**严重、非预期并与研究药物可能有因果关系的**的所有不良事件。

另外，在中国发生的所有 SAE，无论因果关系，辉凌将**尽快，**向中国监管机构报告。

不论是否按照方案规定、研究者手册和公司颁得斯安核心数据表（CCDS）（生效日期 2014 年 1 月 3 日）使用研究药物，都要考虑报告严重不良事件。

8.5 不良事件和严重不良事件的随访

8.5.1 试验期间发生的不良事件的随访

试验期间，研究者必须对每例不良事件进行随访，直至其缓解或受试者的健康状况稳定。

受试者末次访视后，研究者必须随访归类为严重或认为可能与研究药物有因果关系的不良事件，直至其缓解或直至受试者的健康状况稳定。所有相关随访信息必须报告给辉凌制药公司。如果事件为慢性疾病，研究者和辉凌可能会同意不需要进一步随访。

8.5.2 末次访视后发生的严重不良事件的收集

如果受试者末次访视后研究者发现 SAE，并且他/她认为 SAE 可能与研究药物有因果关系，事件必须报告辉凌制药，不论事件是在试验结束后多久出现的

9 统计方法

辉凌制药公司 A/S 的全球生物统计部门将负责统计分析。将在单独的统计分析计划中详细描述所有分析，该计划将在首名受试者首次访视前完成。

9.1 样本量的确定

本试验没有进行正式样本量计算。但是，根据 CFDA《化学药物临床药代动力学研究技术指导原则》[16]，8 例受试者完成研究并且可被包括在 PP 分析集中，认为足以提供关于颇得斯安®灌肠液的药代动力学的充分信息。

假设发生严重方案违背（例如，不依从研究药物保留）和发生不良事件（AE）导致撤出研究的比例共为 20%，则将招募 10 名合格受试者。8 名受试者将完成研究且可纳入到主要（PP 分析集）PK 分析人群中。退出的受试者将被替换以确保至少有 8 名受试者完成研究，其中每个性别至少有 2 名受试者。

9.2 受试者分析

受试者包括所有参加筛选且第-1 天通过入选/排除标准检查的受试者。所有入选/排除标准检查后退出的和中止试验的受试者将按照退出或中止的时间和原因进行总结。

如果受试者在研究药物首次给药前被发现不合格并因此退出试验，将视为筛选失败。筛选失败的例数将在临床试验报告中报告。筛选失败的受试者将不再纳入其他受试者分布的分析总结。

9.3 试验方案偏离

严重方案违背，例如显著不依从性或其他被认为会导致数据无效和影响试验结论的严重的无法预见的方案违背，相关数据将从符合方案分析集中排除出去。

轻度方案违背的数据不会从数据分析中排除出去。

在文件清理完毕和数据库锁定前的数据审查时，研究者和申办方将对方案违背为“轻度”或“严重”进行评估并达成一致。

9.4 分析集

9.4.1 意向治疗分析数据集（ITT）

ITT 分析集将包括所有第 1 天合格受试者的数据。ITT 数据集将用于报告受试者分配情况。

9.4.2 全分析集（FAS）/疗效人群

FAS 将包括所有给药受试者的数据。FAS 将用于报告依从性和所有基线特征（人口统计学、病史、既往和伴随用药以及体格检查）。

9.4.3 符合方案数据集 (PP)

PP 数据集将包括所有用药受试者的数据，因第 9.3 节定义的严重违背方案被排除的受试者除外。PP 分析集将用于报告 PK 终点。

9.4.4 安全性数据集

安全性分析集包括所有给药受试者，并根据接受的实际给药进行分析。

9.5 试验人群

9.5.1 人口统计学和其他基线特征

所有基线信息见数据列表。选择的基线数据将使用 FAS 按照给药组制成表。

9.5.2 病史, 既往 / 合并用药和其他安全评价

将使用描述性统计展示 FAS 的所有数据。

将利用药事管理的标准医学术语集（MedDRA）版本 17.0 或以上对病史进行编码。将对病史进行总结。

将利用世界卫生组织词典（WHO 药物；试验开始时的有效版本），国际非专利名称（INN；也叫做‘通用名’）和治疗用途（解剖学治疗学代码；ATC）对既往和伴随用药进行编码，并且按照 ATC 分类第 1 级（字母顺序），ATC 分类第 2 级（按照频率降序排列）以及给药组/剂量水平，进行总结。

9.5.3 给药依从性

将按照受试者列出用药时间、研究药物用药次数以及纸尿裤的重量增加情况。

9.6 评价指标评估

主要和次要终点与研究药物的 PK 相关。以下描述了这些数据的分析、统计分析以及数据呈现。

9.6.1 一般考虑事项

对于每个剂量和研究部分，将利用 PP 数据集按照给药组列出所有个体浓度数据和 PK 参数并以表格显示。缺失数据不进行插补。

将在总结表、图表和列表中给出所有估计的 PK 参数。

9.6.2 主要药代动力学指标

将由辉凌制药公司 A/S 的实验医学部门进行 PK 分析。使用 Phoenix® (Pharsight 公司，美国)，利用非房室分析（NCA）计算 PK 参数。给药后的实际采样时间点将用于 NCA 分析和个体血浆药物浓度浓度-时间曲线图。对于血浆浓度低于定量下限的样品，在进行药代动力学分析时，在达到 C_{max} 以前取样的样品应以零值计算，在达到 C_{max} 以后取样的样品应以无法定量（Not detectable, ND）计算。将从 NCA 中排除缺失值（例如：没有收集血样或者分析时没有获得数值）。没有计划对“异常值”进行正式分析。

将根据单次给药部分第 1 天到第 2 天、多次给药部分第 5-6 天和第 7-8 天的测定值，估计 PK 参数。

参与λ_z计算 $t_{½}$的数据点选择：

- 利用线性梯形法则计算 AUC；
- 用 Phoenix®自动范围选择功能优化最佳时间点数量，用于计算λ_z；
- λ_z计算中将包括对数线性消除相内至少三个高于 LLQ 的样本。
- 参与λ_z计算的样本选择最终基于以个体血浆药物浓度-时间半对数曲线的肉眼观测。

如可能将根据美沙拉秦血浆浓度时间数据估计以下单次给药和多次给药 PK 参数：美沙拉秦的 AUC、AUC_t、AUC_{tau}、C_{max}、C_{avg}、t_{max}、CL/F、Vz/F、$t_{1/2}$ 及λ_z。将提供 PK 参数统计学汇总。

用第 1 次给药后的 AUC（如果 AUC 未计算，则使用 AUC_t）和第 7 次给药后的 AUC_{tau}评价多次给药后药物在体内可能产生的蓄积。

9.6.3 次要药代动力学指标

如果可能，用第 7 次给药后的体积和尿液中美沙拉秦和 N-乙酰基美沙拉秦的浓度估算 Ae 和 Fe。将提供 Ae 和 Fe 的统计学汇总。

9.7 给药的依从性

按给药列出 FAS 和 PP 分析集中给药期（第 1 至 8 天）给药次数和用药的受试者例数。

9.8 安全性

9.8.1 一般考虑事项

将评估安全性分析数据集的安全性参数。

将根据 MedDRA，版本 17.0 或以上，对不良事件进行编码。将按照受试者列出所有数据。

不良事件将分类为治疗前出现或治疗中出现的事件（定义参见第 8.1 节定义）。只有治疗中出现的不良事件将被列在总结表中。将提供定义为治疗前不良事件的单独数据列表。

缺失数值将作为缺失处理，除了不良事件的因果关系、严重程度、严重性和转归。将使用“最差情况”方法：如果因果关系缺失，不良事件将被视为与研究药物有关；如果不良事件的严重程度缺失，将被视为重度；如果严重性缺失，不良事件将被视为严重不良事件；如果转归缺失并且没有转归日期，不良事件结果将被视为“尚未康复”。

9.8.2 不良事件

治疗前不良事件

将按照受试者列出从签署知情同意书到第 1 天接受首剂研究药物之间发生的事件。

治疗中出现的不良事件概述

将给出治疗中出现的不良事件总结表，包括每个治疗中报告至少 1 例不良事件的受试者人数和比例以及报告的下述每种类型事件的总数：

- 所有不良事件；
- 重度不良事件；
- 严重不良事件；
- 药物不良反应（ADRs）

不良事件与研究药物的因果关系被视为“可能无关”的将归类为与研究药物无关。不良事件与研究药物的因果关系被视为“可能有关”的将归类为与研究药物有关，并考虑是药物不良反应；

- 导致退出研究的不良事件
- 死亡

治疗中出现的不良事件的发生率

将按照 MedDRA 系统器官分类（SOC）和首选术语，准备每个治疗治疗中出现的不良事件发生率的总结表。将给出报告不良事件的受试者人数和比例（%）以及报告的事件数量。将准备下述总结表：

- 所有治疗中出现的不良事件；
- 按照因果关系分类的不良事件；
- 按照严重程度分类的不良事件；

- 按照严重程度分类的药物不良反应

严重不良事件、死亡和其他重要不良事件

将提供严重不良事件、死亡和其他重要不良事件（如果有）的单独列表。

9.8.3 实验室检查数据

将按照时间和治疗，列出每例受试者的临床化学、血液学和尿液分析参数。将标出参考范围外的数值。

另外，将按照时间和治疗总结安全性实验室参数。将给出从基线（即研究药物给药前的最后一次评估）到第 2 天访视（单次给药部分）或第 9 天访视（多次给药部分）变化的变化表。变化表将给出受试者总数、出现变化的受试者人数、每次访视出现变化的受试者比例，利用较低数值、正常值和较高数值进行分类。将根据本地实验室提供的参考范围定义较低数值、正常值和较高数值。

- 较低数值：低于参考范围下限的数值
- 正常值：在参考范围内的数值
- 较高数值：高于参考范围上限的数值

对于分类尿液分析参数，变化表将总结基线时数据‘缺失’以及给药期间‘出现’数值的受试者人数和比例。

- 缺失：测量变量没有获得数值。
- 出现：获得测量变量的任何数值。

将根据研究者的判断，利用分类“正常”、“异常，没有临床意义”和“异常，有临床意义”，准备相似的变化表。

9.8.4 生命体征

将按照时间展示生命体征的每个参数（血压、心率、腋下温度和呼吸频率），并按照治疗进行总结。将给出从基线（即研究药物用药前的最后一次评估）到试验结束检查的变化的变化表，按照研究部分进行总结。

9.8.5 ECG

将列出每例受试者的所有 12 导联 ECG 数据，并按照时间和给药进行总结。另外，将根据研究者的判断，利用分类“正常”、“异常但是没有临床意义”和“异常有临床意义”，准备变化表。

9.8.6 体格检查

将按照受试者列出筛选和试验结束检查时的体格检查结果。

9.9 中期分析

没有计划期中分析。

10 数据处理

10.1 原始数据和原始文件

原始数据-ICH 定义

原始数据是指通过临床发现、观察或试验中其他活动产生的原始记录和原始记录的合格副本中的全部信息，这些信息对于试验的复原和评价是必要的。原始数据包含在原始医疗文件中（原始记录或合格副本）。

原始医疗文件—ICH 定义

原始医疗文件是指最早的文件、数据和记录（例如：医院记录、临床和办公室图表、实验室记录、备忘录、受试者日记或评价表、药物发放记录、自动化仪器记录的数据、验证后被认定为准确复印件的合格复印件或副本、微缩胶片、照相底片、微缩胶卷或磁介质、X 光、受试者档案以及参加临床试验的药房、实验室和医药技术部门保存的记录）。

临床试验特定原始数据定义——辉凌制药

研究者必须保存受试者记录。

在特定原始数据表格中记录受试者病史、体格检查结果和其它临床相关结果、人口统计学数据和 AE，然后转录到每个受试者的的 CRF。实验室参数将从数据库中打印并粘贴在当地文件中。实验室结果将由研究者转录到 CRF 中。12 导联 ECG 评估结果将被转录到 CRF 的特定部分，并且标注为正常、异常没有临床意义或异常有临床意义。必要时，还将在 CRF 中对具体的异常情况进行详细的描述。

10.2 CRF

对于入组的每例受试者，将由研究者完成和签署 CRF。完成后的原始 CRF 是申办方的专属财产，没有申办方的书面许可，不能以任何形式提供给第三方，相关监管机构或 EC 的授权代表除外（参见第 14 节）。

研究者有责任确保完成、审查及批准所有的 CRF。所有 CRF 必须用黑色或蓝色墨水笔清楚地填写。进行更正时，用线划出错误的信息并写下修订内容。所有更改必须经过批准并注明日期。这些签名用于证明 CRF 中所含信息真实。研究者始终对 CRF 上输入的所有临床和实验室数据的准确度和真实性负有最终责任。受试者原始文件是在研究中心保存的医生记录的受试者病历。必须及时将试验数据输入到 CRF 中。

10.3 数据管理

数据管理将外包给有资质的合同研究组织（CRO）。将由 CRO 负责创建数据管理计划。将在数据收集开始前公布数据管理计划，并将描述所有功能、过程和数据收集、清理以及验证的规范。

将按照外包 CRO 的标准操作规程进行数据录入。用于数据采集的系统需要充分验证。将采用 MedDRA（版本 17.0 或以上）和 WHO 药物词典，对 AE、病史和合并用药进行医学编码。当所有数据已处理、疑问已解决、医学编码已完成以及方案违背和数据列表审查发现的任何问题解决时，将锁定数据库，并删除所有更新访问。最终数据库将根据辉凌的数据规范进行构建。

10.4 附加信息的提供

如果需要，研究者将向申办方提供试验相关附加数据或隐匿姓名的相关原始记录的副本。特殊情况下或政府查询时，可能需要提供完整的试验文件，但是需要根据适用的要求保护受试者的保密信息。

11 监查程序

11.1 定期监查

监查员将定期联系并拜访研究者，以确保试验遵守试验方案、国际协调会议-药物临床研究质量管理规范（ICH-GCP）、标准操作规程和相关法规的要求，确保保留了试验相关原始记录，并确保 CRF 内容与原始数据相比的完整性、准确性和可核实性，药物清点验证和安全性报告指南依从性。

研究者将允许监查员直接访问所有原始数据，包括电子医学记录和/或有助于数据验证的文件。研究者将配合监查员，确保解决可能发现的所有偏差。希望研究者能够在这些访视期间与监查员会面。首名受试者在研究中心被分配接受给药后不久即进行监查访视。

将进行 100%原始数据验证（SDV）。将在试验的监查手册中详细描述 SDV 的过程。

11.2 稽查和视查

在获得合理的通知后，研究者将提供所有研究相关的原始数据和记录给辉凌委任的质量保证稽查人员，或本国或外国的法规检查人员，或对试验进行稽查/检查的 EC 代表。

稽查或视察的主要目的是确认与试验药品评价有关的所有数据均依据 GCP 和相关法规的要求进行处理和报告。

研究者必须告知受试者，并且在知情同意文件中告知受试者授权的辉凌代表以及监管机构和 EC 代表可能都会查看她们的医疗记录。稽查/视察期间，稽查员/视察人员可能会复制医疗记录的相关部分。这些复印件上除了筛选/随机化编号，不会出现任何个人身份信息。

监管机构或 EC 进行视察时，研究者应立即通知辉凌。

11.3 受试者信息的机密性

研究者将确保受试者数据保密。在递交给辉凌的 CRF 或任何其他文件中，将无法通过姓名识别受试者，而是通过试验分配编号组成的识别系统进行识别。不递交给辉凌的文件，例如保密的受试者识别码和已签署的知情同意文件，将由研究者严格保密。

12 试验操作的变更

12.1 试验方案修订

本试验方案的任何变更都将记录在试验方案修正中，并在实施前由研究者与申办方达成一致。任何修正都将递交给 EC。

对于可能影响受试者安全或需要对试验范围/设计进行变更的任何修订，例如对研究药物的剂量或暴露时间增加、受试者人数增加、添加新检查或程序或为了监测安全性而去掉一项检查，均需要获得 EC 批准。

然而，为消除对试验受试者的直接危害而对方案进行的变更，可在 EC 批准前执行。

将根据当地法规递交方案修正，通知 EC 和 CFDA。

12.2 试验试验方案偏离

如果发生试验方案背离，研究者必须通知监查员，必须审查并讨论背离的相关意义。任何背离均必须记录，或者作为 CRF 中质疑的回答进行记录，或者在方案背离报告中记录，或者两者结合。方案背离报告表将由辉凌保管。方案背离报告以及支持性证明文件必须保留在研究者文件夹和试验主文件夹中。

12.3 提前终止试验

研究者（关于他/她的参与）和辉凌都将保留在任何时间终止试验的权利。如果有必要终止试验，将在咨询双方后就终止步骤达成一致。终止试验时，辉凌和研究者将确保充分考虑保护受试者的最佳利益。将通知监管机构和伦理委员会。

此外，辉凌将保留终止个别研究中心参与试验的权利。可能导致试验终止的情况包括但是不限于不能充分遵守方案要求和受试者入选不能达到可接受的比例。

13 报告和发表

13.1 临床试验报告

本试验期间收集的数据和信息，由辉凌撰写的临床试验报告，并递交给署名研究者审阅并署名。

13.2 研究数据的机密性和所有权

与研究药物或本试验有关的任何保密信息，包括源自本试验的任何数据和结果，为辉凌的专有财产。研究者和参加本试验的任何其他人员应保护这些属于辉凌的专有信息的保密性。

13.3 发表和公开披露

13.3.1 发表政策

试验结束时，研究者可以和辉凌公司合作撰写一篇或多篇文章，用以共同发表。

依据 ICMJE 标准（参见当前官方版本：http/www.ICMJE.org）确定著作权。作者总数基于相关期刊或者会议的指导原则确定。如果出版物的内容中存在任何分歧，将在出版物中公平且充分地体现研究者和辉凌公司的意见。

任何参加本试验实施的外部 CRO 或者实验室都没有本试验的发表权。

如果研究者希望独立发表/介绍关于试验的任何结果，稿件/出版物的草稿必须在投稿前以书面形式递交辉凌公司，以获得公司的意见。辉凌公司将在收到稿件草案的 4 周内给出意见。本声明除了限制透露辉凌公司的知识产权，并未授予辉凌公司对出版内容的任何编辑权。如果辉凌公司认为出版物中的内容可申请专利，在提交的专利申请公布前，不允许在科学出版物上发表。这种情况下，研究者可以决定修改或推迟发表，使辉凌公司有充足时间寻求该发明的专利保护。

13.3.2 公开披露政策

ICMJE 成员杂志已经采纳试验注册政策作为发表条件。该政策要求所有临床试验在一个公开的临床试验注册网上注册。因此，辉凌将负责在适当的公开注册网（即 CFDA 指定的 http://www.chinadrugtrials.org.cn/）上注册本试验。

14 伦理监管

14.1 伦理委员会

EC 将审查试验方案和任何修正以及招募所用的广告。EC 将审查受试者信息页和知情同意书及其更新（如果有）以及提供给受试者的任何书面材料。

14.2 监管当局授权/批准/通知

辉凌医药公司根据中国法律的相关规定，已从国家食品药品监督管理局获得本试验的药物临床试验批件（批件号：2012L01750）。在受试者接受任何试验相关程序包括入选资格筛选检查之前，必须获得所有相关伦理批准。

14.3 试验结束和试验结束通知

试验结束定义为试验中末例受试者进行末次访视的日期。将根据当地法规报告试验结束通知。

14.4 试验的伦理行为准则

本试验将根据来自于《赫尔辛基宣言》的伦理学原则、批准的试验方案、GCP 和适用的法规要求进行。

14.5 受试者告知信息和知情同意

在研究者（或研究者委托人员）适当解释试验目的、方法、预期受益、潜在危害、需要受试者配合、保险、保密性以及与受试者参加试验的决定有关的任何其他方面后，获得每名受试者自愿给出的书面同意。获得同意前，必须给予受试者足够时间考虑是否参与试验。知情同意文件必须在受试者暴露于任何试验相关步骤包括入选/排除标准核对筛查检查之前由受试者和研究者（将试验相关信息提供给受试者）签名。

研究者（或研究者委托人员）应解释受试者可完全自由的拒绝参加试验或在任何时间退出试验，不会对他/她的进一步治疗有任何影响，且无需说明原因。

受试者将收到受试者信息及其署名后的知情同意书的一份副本。

如果获得了与受试者继续参加本试验的意愿相关的新信息，将向 EC 递交一份新的受试者信息和知情同意书。将告知受试者该新信息，并重新获得其同意。

将根据适用的监管要求通知每名受试者，监查员、辉凌委派的质量保证稽查员，EC 代表或监管当局视察员可能会审查原始记录和数据。将按照国家/当地法规进行数据保护。

14.6 受试者信息卡

不提供受试者信息卡，因为从研究药物首次给药日至试验结束受试者都住在医院。

14.7 依从性参考文件

赫尔辛基宣言、综合 ICH-GCP、以及中国的其他国家法律，将构成伦理和监管行为的主要参考指导原则。

15 责任和保险

15.1 ICH-GCP/GCP 责任

辉凌、监查员和研究者的责任与 ICH-GCP 综合指导原则以及试验实施国的相关监管要求规定的相一致。研究者负责遵循 ICH-GCP 中规定的研究者责任，负责根据批准的研究方案或签署的修订方案分发研究药物以及整个试验期间研究药物的安全保存和安全处理。

15.2 责任和保险

为了防备受试者发生与研究药物或参加试验有关的任何损害或损伤，辉凌已经按照参与试验国家的法律，签订了涵盖辉凌、研究者和参与试验的其他人员责任的保险。

16 存档

16.1 研究者文档

研究者负责根据 ICH-GCP/GCP 保存所有记录，以便可以充分了解研究中心进行的试验。如果辉凌公司没有提供进一步的说明，则研究者需要至少保存 15 年的所有相关的试验完成或停止后的试验。

研究者负责完成和保存机密的患者识别码，该识别码提供有姓名的患者源记录和提供给辉凌的无名 CRF 数据之间唯一的关联。研究者必须安排保存此患者识别记录和签署的知情同意书到试验完成或停止后至少 15 年。

未经辉凌公司和研究者的书面授权，不得销毁试验文件。研究者必须在通知辉凌公司后，才能把试验文件转移存储地点或交由第三方保管。如果研究者退休或研究中心无法继续保管试验文件，试验文件可以移交给辉凌公司保管。

16.2 试验主文档

辉凌将按照 ICH-GCP/GCP 和适用的监管要求存档试验主文档。

17 参考文献

1. Jang ES, Lee DH, Kim J, et al. Age as a clinical predictor of relapse after induction therapy in ulcerative colitis. Hepatogastroenterology. 2009;56:1304-9.
2. Jie Zhao, Siew C. Ng, Yuan Lei, et al. First Prospective, Population-Based Inflammatory Bowel Disease Incidence Study in Mainland of China: The Emergence of “Western” Disease. Inflamm Bowel Dis. 2013; 9: 1839-45.
3. Qin OY, et al. Experts’consensus on the diagnosis and management of inflammatory bowel disease. Chin J Intern Med 2008; 47:73-9.
4. Baumgart DC and Sandborn WJ. Inflammatory bowel disease: clinical aspects and established and evolving therapies. Lancet. 2007; 369:1641-57.
5. Carter MJ, Lobo AJ, et al. Guidelines for the management of inflammatory bowel disease in adults. Gut. 2004; 53:V1-16.
6. Marteau P, Seksik P, et al. Recommendations for clinical practice for the treatment of ulcerative colitis. Gastroenterol Clin Biol. 2004; 28:955-60.
7. Jacobsen, BA, et al. Availability of mesalazine (5-aminosalicylic acid) from enemas and suppositories during steady-state conditions. Scand J Gastroenterol. 1991. 26: p. 374-8.
8. Ahnfelt-Ronne, I. and O.H. Nielsen, The antiinflammatory moiety of sulfasalazine, 5-aminosalicylic acid, is a radical scavenger. Agents Actions, 1987. 21(1-2): p. 191-4.
9. Fujiwara, M., K. Mitsui, and I. Yamamoto, Inhibition of proliferative responses and interleukin 2 productions by salazosulfapyridine and its metabolites. Jpn J Pharmacol, 1990. 54(2): p. 121-31.
10. Gionchetti, P., et al., Scavenger effect of sulfasalazine, 5-aminosalicylic acid, and olsalazine on superoxide radical generation. Dig Dis Sci, 1991. 36(2): p. 174-8.
11. Hoult, J.R. and P.K. Moore, Effects of sulphasalazine and its metabolites on prostaglandin synthesis, inactivation and actions on smooth muscle. Br J Pharmacol, 1980. 68(4): p. 719-30.
12. Wilding IR HG, Brown J, Sparrow RA, Kenyon CJ. Scintigraphic evaluation of four rectally administered PENTASA® formulations. Research report. Study code: PPL-104., Vanløse, Denmark: Ferring A/S; 1996.
13. Rambaud JC MP. A comparative study of systemic absorption of mesalazine enema and foam (1g/application). Clinical study report, Gentilly, France: Ferring S/A; 1996. Protocol no. PENTAMOUKIN/93/02.
14. Bondesen S., et al. Pharmacokinetics of 5-aminosalicylic acid in man following administration of intravenous bolus and per os slow-release formulation. Dig Dis Sci. 1991. 36: 1735-40.
15. Myers, B., et al. Metabolism and urinary excretion of 5-amino salicylic acid in healthy volunteers when given intravenously or released for absorption at different sites in the gastrointestinal tract. Gut. 1987. 28: p. 196-200.
16. CFDA.《化学药物临床药代动力学研究技术指导原则》.2005.

附件

附件 1 研究药物给药介绍

***注意：**本用法说明摘自药物标签并对患者自己给药做了示范。在本 PK 研究中将由试验护士给药。带有铝箔包装的研究药物瓶应提前在水浴中加热至 30~37℃。*

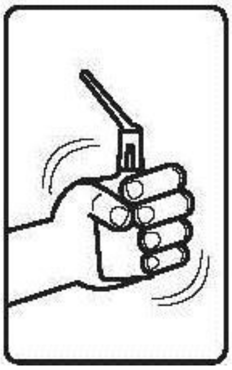

1. 在即刻使用前，将灌肠剂药瓶从铝箔包装中拿出并充分摇匀。

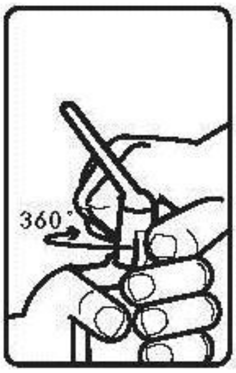

2. 顺时针旋转管口一圈以打开密封（管口应处于和旋转前相同的方向）。

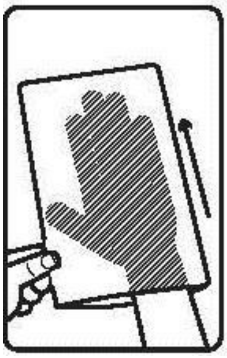

3. 将手放置于包装中提供的一个塑料袋中。

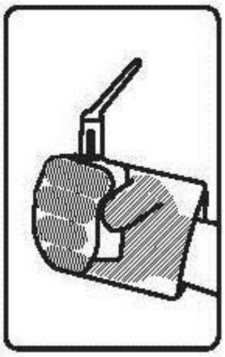

4. 如图示握住容器。

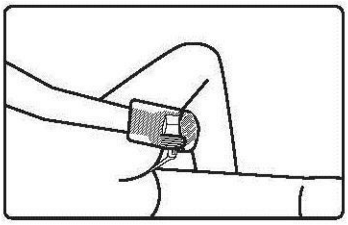

5. 左侧卧位躺下，左腿伸直，右腿弯曲，用于保持平衡并给药。小心的将灌肠剂药瓶的前端放入直肠中，并用手掌充分而稳定的力量将瓶内药物推入。瓶内药物应在最长 30 至 40 秒内完成给药。

6. 当瓶子清空后，撤回药瓶前端并保持药瓶处于压缩状态。

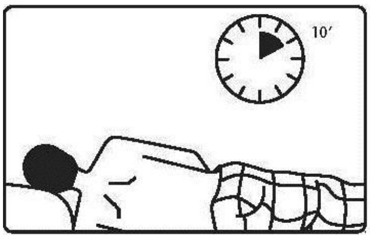

7. 灌肠剂应保留在肠道内。放松并保持给药姿势 5 至 10 分钟，或直到想排出灌肠剂的感觉消失为止。

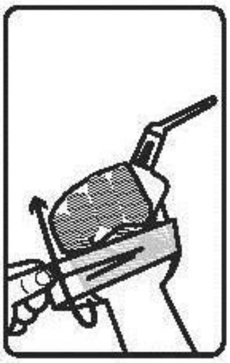

8. 将塑料袋套在空药瓶外面，丢弃并洗手。

附件 2 使用温水灌肠排空肠道的说明

1. 使用已在中国获批的灌肠器作为给药装置。

2. 加热 1000 mL 纯水至约 30~37℃。

3. 受试者采用左侧卧位。

4. 用凡士林润滑灌肠器的肛管，插入直肠，插入深度约为 10~15 cm。肛管与装有 1000 mL 水的水袋连接，水袋距肛门高度约 40~60 cm。（如果水袋容量小于 1000 mL，可分几次将水注入水袋，以保证总量为 1000 mL。）

5. 通过肛管将 500~1000 mL 水注入直肠。

6. 拔出肛管后受试者左侧卧位卧床 5-10 分钟，然后如厕并排空肠道。

7. 由研究者决定，步骤 4-6 可重复进行一次，例如第一次注入的水少于 500 mL 的情况。温水灌肠不会超过两次。每名受试者注水总量为 1000 mL。

CLINICAL TRIAL PROTOCOL

An Open-Label, Single and Multiple Dose Study Investigating Pharmacokinetics and Safety of PENTASA® Enema in Healthy Chinese Subjects

000166

EudraCT Number:	Not applicable
IND Number:	Not applicable
**Investigational Medicinal Product:**	PENTASA® enema (Mesalazine enema, 1 g/100 mL)
**Indication:**	Left-sided ulcerative colitis
**Phase:**	I
**Name and Address of Sponsor:**	Ferring International Pharma-Science Centre (China) Co. Ltd.<br>Suite 10, Tower B, No. 28 Jianguomennei Street<br>Minsheng Financial Centre, Dongcheng District, Beijing, China 100005
**GCP Statement:**	This trial will be performed in compliance with GCP.

The information in this document is confidential and is proprietary to Ferring International Pharma-Science Centre (China) Company Limited or another company within the Ferring Group. It is understood that information in this document shall not be disclosed to any third party, in any form, without prior written consent of an authorised officer of Ferring International Pharma-Science Centre (China) Company Limited or another company within the Ferring Group.

SYNOPSIS

**TITLE OF TRIAL**<br>An Open-Label, Single and Multiple Dose Study Investigating Pharmacokinetics and Safety of PENTASA® Enema in Healthy Chinese Subjects	
**SIGNATORY INVESTIGATOR(S)**<br>Prof. Jing Zhang, PhD	
**TRIAL SITE**<br>Huashan Hospital, Fudan University, 12 Wulumuqi Zhong Lu, Shanghai	
**PLANNED TRIAL PERIOD**<br>**First subject first visit** 06/2015<br>**Last subject last visit** 09/2015	**CLINICAL PHASE**<br>I

Background/Rationale

Mesalazine is the main first-line therapy for induction of remission and maintenance therapy of mild to moderate active Ulcerative colitis. Ferring medicinal product of mesalazine rectal suspension, PENTASA® enema had been approved in 71 countries as of February 2014.

This study is part of the clinical development program for PENTASA® enema in China, and is designed to determine the single and multiple dose pharmacokinetics (PK) and safety of PENTASA® enema in healthy Chinese subjects.

OBJECTIVES

Primary Objectives

To determine single and multiple dose pharmacokinetics of mesalazine (PENTASA®, 1 g/100 mL) after rectal administration in healthy Chinese subjects.

Secondary Objectives

To assess the safety of single and multiple dose of PENTASA® enema after rectal administration in healthy Chinese subjects.

To determine total cumulative amount of mesalazine and N-acetyl-mesalazine excreted in urine.

ENDPOINTS

Primary Endpoints

- Pharmacokinetics:
  - Single dose period (following the 1st dose): AUC_t, C_{max}, t_{max} of mesalazine.
  - Multiple dose period (following the 5th dose): AUC_{tau}, C_{max}, t_{max} of mesalazine
  - Multiple dose period (following the 7th dose): AUC_{tau}, C_{max}, t_{max} of mesalazine.

Secondary Endpoints

- Pharmacokinetics:
  - Single dose period (following the 1st dose): AUC, CL/F, V_z/F, λ_z and $t_{½}$ of mesalazine.
  - Multiple dose period (following the 5th dose): AUC, CL/F, V_z/F, C_{avg}, λ_z and $t_{½}$ of mesalazine.
  - Multiple dose period (following the 7th dose): AUC, CL/F, V_z/F, C_{avg}, λ_z and $t_{½}$ of mesalazine.
  - Total cumulative amount of mesalazine and N-acetyl-mesalazine excreted in the urine (Ae) up to 24 h following the 7th dose of mesalazine.
- Type, frequency and intensity of adverse events.
- Clinically significant changes in vital signs, 12-lead ECG, clinical chemistry, haematology, and urinalysis.

METHODOLOGY

This is an open-label, single and multiple dose pharmacokinetic study. There will be one dose panel with at least 8 healthy Chinese subjects.

Screening is from Day -14 to Day -1. During Day -14 to -2 at Screening period, subjects will undergo examinations and laboratory tests, including a general physical examination, vital signs, 12-lead ECG, digital rectal examination, stool test, serum pregnancy test (females), serology/virology, clinical chemistry, haematology, urinalysis, urine drug screen and alcohol breath test and enema retainment test (30 min).

Subjects who pass the tests during Day -14 to -2 will be admitted to trial unit on Day -1. Inclusion/exclusion criteria check, including vital signs, serum pregnancy test (females), urine drug screen, alcohol breath test, clinical chemistry, haematology, urinalysis and an enema retainment test for 8 h (including bowel cleaning by water enema prior to enema retainment test), will be performed on Day -1. Subjects who pass the tests on Day -1 are eligible to IMP treatment.

Eligible subjects will be admitted to Treatment Period (Day 1 to 8).They will receive a daily rectal dose of PENTASA® enema (1 g/100 mL) 9-10 p.m. on Day 1-7 and be required to retain the enema for at least 8 h following IMP dosing. Before 1st and 7th dose, bowel cleaning will be performed approximately 2 h before IMP administration using water enema. Subjects will be required to go to toilet after rectally being introduced 500~1000 mL purified water. Approximately 2 h before each of 2nd to 6th doses, subjects will be required to go to toilet and evacuate their bowls as clean as

possible, but water enema will not be used.

Blood samples for analysis of mesalazine will be collected repeatedly up to 24 h following 1st, 5th and 7th doses. The sampling times associated with each of the three doses are: immediately prior to dosing and 0.5, 1, 2, 3, 4, 5, 6, 7, 8, 10, 12, 14, 16, 20 and 24 h post-dose (before the next dose). Approximately 4 mL blood will be sampled at each time point.

Urine samples will be collected for the determination of mesalazine and its main metabolite, N-acetyl-mesalazine. 5~10 mL blank urine will be collected prior to the 1st dose and all urine during intervals of 0-12 h and 12-24 h following the 7th doses will be collected.

Vital signs will be measured on each day during Treatment Period (Day 1 to 8). Clinical chemistry, haematology and urinalysis will be performed on Day 2.

On Day 9, subjects will undergo an End-of-trial examination including physical examination (excluding height), vital signs, 12-lead ECG, serum pregnancy test (females), clinical chemistry, haematology, and urinalysis. Subjects will only be discharged from the trial site if results of End-of-trial examination are considered ‘normal’ or ‘abnormal, not clinically significant’. In case any indicator is considered ‘abnormal, clinically significant’ at this time, additional managements will be taken at the discretion of the Investigator before discharging of the subjects.

NUMBER OF SUBJECTS

No formal sample size calculations have been performed for this trial. However, based on CFDA guidance on clinical pharmacokinetic study, 8 subjects are considered sufficient to provide adequate information on the pharmacokinetics of mesalazine. Assuming a dropout rate of 20%, 10 eligible subjects will be recruited.

Subjects who withdraw from the trial could be replaced to achieve at least 8 completers, in which at least 2 completers of each sex.

CRITERIA FOR INCLUSION / EXCLUSION

Chinese subjects who meet the inclusion criteria and do not meet exclusion criteria

Inclusion Criteria

1. Signed written Informed Consent Document(s) (ICD)
2. 18 - 45 years of age. The subjects must be at least 18 years (including the 18th birthday) and no more than 45 years (up to the day before the 46th birthday) when they sign the ICD
3. Body weight ≥ 50 kg (male) or ≥45 kg (female) and body mass index (BMI) of 19-24 kg/m^2 (both inclusive)
4. Healthy according to medical history, physical examination, 12-lead ECG, vital signs, and laboratory profile of blood and urine

5. Healthy rectal and anal status based on digital rectal examination and stool test
6. Healthy intestinal track status according to medical history with normal defecation frequency (once daily to three times per week during past 3 months)
7. Capable to retain a placebo enema for at least 30 minutes during Day -14 to -2, with diaper weight increase no more than 20 g
8. Capable to retain a placebo enema for at least 8 hours on Day -1, with diaper weight increase no more than 20 g
9. Negative virology/serology for human immunodeficiency virus antibodies (HIV-Ab), hepatitis B surface antigen (HBs-Ag), hepatitis C virus antibodies (HCV-Ab), and treponema pallidum antibodies (TP-Ab) at Screening
10. Negative urine drug screen and alcohol breath test
11. Agrees to use a barrier contraception method (e.g. condom) from signing the ICD to 3 days after End-of-trial examination
12. Non-smoker or light smoker (less than five cigarettes, or equivalent, per day). In the latter case, the subject must abstain from smoking during the residential stay at trial site
13. For female subjects: a) history of normal menstrual cycles with a mean length of 28-35 days within 6 months prior to Screening, b) history of normal menstruation of 3-7 days within 6 months prior to Screening and, c) end-of-menstruation day is within 7 days prior to Day -1

Exclusion Criteria

1. Presence or a history of clinically significant diseases of the renal, hepatic, gastrointestinal, cardiovascular, musculoskeletal systems or presence or history of clinically significant psychiatric, immunological, endocrine or metabolic diseases
2. Cancer within the last 5 years except for adequately managed basal cell carcinoma and squamous cell carcinoma of the skin
3. Presence or history of severe allergy or anaphylactic reactions, such as hypersensitivity to salicylates
4. Present pregnancy or breastfeeding
5. History within the last two years or current abuse of drugs or alcohol (>40 g of alcohol/day equivalent to >1 L of beer/day, 0.5 L of wine/day, or 6 glasses (2 cL) of liquor/day)
6. History of previous gastrointestinal surgery
7. Present diseases of the ano-rectum, including fistulas, anatomic irregularities, rectal tone compromising the ability to hold the enema
8. Intake of prescribed medication, over-the-counter (OTC) medication, or herbal medicines, within 2 weeks or 5 half-lives of the drug, whichever is longer, prior to 1st dose of mesalazine. Topical treatments of bacterial or fungal infection are allowed if stopped before first dose of IMP
9. Participate in any clinical trial within the last 12 weeks preceding Screening or longer if judged by the investigator to possibly influence the outcome of the current study
10. High daily consumption of caffeine-containing beverages (e.g. more than five cups of coffee or equivalent) within the last 12 weeks preceding Screening with a risk of withdrawal symptoms arising during the study that may confound the safety evaluation
11. Blood donation or major blood loss (≥500 mL) within the last 12 weeks preceding the first day of dosing

12. Previously recruited in this study and has been treated with at least one IMP
13. Mental incapacity or language barrier precluding adequate understanding or co-operation
14. Female subjects whose menstruation likely overlaps the planned Treatment Period (Day 1 to 8)
15. Considered by the investigator to be unsuitable to participate in the study for any other reason

MEDICINAL PRODUCTS

Investigational medicinal product (IMP):
Mesalazine enema (PENTASA® enema, 1 g/100 mL)

Non-Investigational medicinal products (NIMP):
Placebo enema (100 mL)

DURATION OF TREATMENT

Subjects will be admitted to trial site on Day -1. PENTASA® enema will be administered as a daily dose on Day 1-7. Blood sampling will start on Day 1 and end on Day 8, and urine sampling will be from Day 7 to Day 8. Subjects will be discharged from trial site on Day 9. The total duration of staying at trial site is around 10 days.

TRIAL PROCEDURES / ASSESSMENTS

After signing Informed Consent Documents (ICD) at Screening, all subjects will undergo a general physical examination, vital signs, 12-lead ECG, digital rectal examination, stool test, serum pregnancy test (females), serology/virology, clinical chemistry, haematology, urinalysis, urine drug screen and alcohol breath test. Enema retainment test (30 min) will be performed to exclude subjects who are sensitive to rectal administration (enema retainment test must be done prior to DRE). A placebo enema (100 mL) will be administered to subjects. The placebo enema is pre-heated to 30~37°C, and dosed in the same way as IMP administration. A diaper, which has been weighted, will be applied to a subject. The diaper will be weighted again after 30 minutes post placebo enema administration. The diaper should be weighted no more than 10 minutes following being taken off. Eligible subjects should retain the enema for at least 30 minutes with less than or equivalent to 20 g diaper weight increase. Menstrual history (females) will be checked. The qualified female subjects' menstruation will not likely overlap Treatment Period. Inclusion/Exclusion criteria will be checked based on available information of subjects during Day -14 to -2.

On Day -1, subjects will undergo an inclusion/exclusion criteria check including vital signs, serum pregnancy test (females), urine drug screen, breath alcohol test, clinical chemistry, haematology, urinalysis and menstrual history (females). However, clinical chemistry, haematology, urinalysis will not be performed if they were performed within 7 days before Day -1 (i.e. between Day -8 to Day -2). On Day -1, an enema retainment test (8 h) will be performed. (Water enema will be used to clean the bowel approximated 2 h prior to the test.) A placebo enema (100 mL) will be

administered to subjects at least 22 h prior to 1st dose of IMP administration. A diaper, which has been weighted, will be applied to a subject. The diaper will be weighted again after 8 h post placebo enema administration. The diaper should be weighted no more than 10 minutes following being taken off. Eligible subjects should retain the enema for at least 8 h with less than or equivalent to 20 g diaper weight increase. Eligible subjects will be considered as successfully screened and allocated to the IMP dosing.

During Treatment Period (Day 1 to 8), subjects will receive a daily rectal dose of PENTASA® enema (1 g/100 mL) 9-10 p.m. on Day 1-7 and be required to retain the enema for at least 8 h after IMP dosing. The following procedures will be conducted to improve and evaluate the compliance of IMP retainment:

- Subjects will be fasted and abstained to water 2 h pre-dose (i.e. 7-8 p.m.) until 8 h post-dose (i.e. 5-6 a.m. in the morning of next day).
- Approximately 2 h before each dose, subjects will be required to go to toilet and evacuate their bowels as clean as possible. Water enema will be used to facilitate bowel evacuation before 1st and 7th doses, while it will not be used before 2nd-6th doses.
- A visit to toilet to evacuate bladder is required approximately 10 min before each dose.
- After IMP is pre-heated to 30~37°C inside its foil pack, the IMP will be wiped dry and weighted without foil pack. After IMP administration, the weight of used IMP will be weighted again. The weight of unused and used IMP will be recorded.
- Adult diaper will be used to evaluate the compliance of IMP retainment. The weight of a new (unused) diaper will be measured and recorded, and afterwards the diaper will be applied to a subject. After 8 h post-dose, the diaper will be weighed again. The diaper should be weighted no more than 10 minutes following being taken off. If diaper weight increases more than 20 g after 1st or 7th dose, or if diaper weight increases more than 50 g after one of 2nd to 6th dose, the corresponding subject will be withdrawn from the trial.
- Subjects who urinate within 8 h post-dose will use bedpans in bed.
- After diaper weight is measured, subjects will be required to visit to toilet during 8-10 h post-dose and the defecation time (as time interval) will be recorded.

Blood samples for analysis of mesalazine will be collected repeatedly up to 24 h following 1st, 5th and 7th doses. The sampling times associated with each of the three doses are: immediately prior to dosing and 0.5, 1, 2, 3, 4, 5, 6, 7, 8, 10, 12, 14, 16, 20 and 24 h post-dose (before the next dose). Approximately 4 mL blood will be sampled at each time point.

Urine samples will be collected for the determination of mesalazine and its main metabolite, N-acetyl-mesalazine. 5~10 mL blank urine will be collected prior to the 1st dose and all urine during intervals of 0-12 h and 12-24 h following the 7th doses will be collected.

Vital signs will be measured on each day from Day 1 to Day 8. Clinical chemistry, haematology, and urinalysis will be measured on Day 2.

On Day 9, subjects will undergo an End-of-trial examination including physical examination (excluding height), vital signs, 12-lead ECG, serum pregnancy test (females), clinical chemistry, haematology, and urinalysis. Subjects will only be discharged from the trial site if results of End-of-trial examination are considered 'normal' or 'abnormal, not clinically significant'. In case any indicator is considered 'abnormal, clinically significant' at this time, additional managements will be taken at the discretion of the Investigator before discharging of the subjects.

The duration of residential stay is from Day -1 to Day 9. Approximately total 260 mL will be sampled from each subject (200 mL for PK blood sampling and 60 mL for safety tests). Standardised meals will be provided during Day -1 to Day 9.

No concomitant medication except necessary for AEs is allowed during the study period. Concomitant medication must be recorded if administered.

STATISTICAL METHODS

Sample size:

No formal sample size calculations have been performed for this trial. However, based on CFDA guidance on clinical pharmacokinetic study, 8 subjects are considered sufficient to provide adequate information on the pharmacokinetics of mesalazine. Assuming a dropout rate of 20%, 10 eligible subjects will be recruited. Subjects who withdraw from the trial could be replaced to achieve at least 8 completers (at least 2 completers of each sex).

Pharmacokinetic analysis:

Single dose: PK parameters will be estimated using non-compartmental analysis. PK parameters will be calculated based on measurements taken from Day 1 to Day 2 and presented in summary tables, graphs and listings.

Multiple dose: PK parameters will be estimated using non-compartmental analysis. PK parameters will be calculated based on measurements taken from Day 5-6 and Day 7-8, respectively, and presented in summary tables, graphs and listings. Ae following the 7th dose will be calculated and presented in summary tables and listings.

Accumulation will be evaluated from AUC (AUC_t if AUC is not calculated) after 1st dose and AUC_{tau} after the 7th dose.

Safety analysis:

Adverse events will be presented in summary tables and listings. Safety laboratory examinations, vital signs and 12-lead ECG will be presented in summary tables and listings including change

from baseline. Data will also be presented in scatter plots and profile diagrams.

Table 1 Trial Flow Chart

Assessment	Screening		Treatment Period								End-of-trial examination
**Day**	**-14 to -2**	**-1**	**1**	**2**	**3**	**4**	**5**	**6**	**7**	**8**	**9**
Informed consent	X										
Inclusion/exclusion criteria	X	X	X								
Demographics	X										
Medical history	X										
Physical examination	X										X [a]
Digital rectal examination	X										
Stool test	X										
Vital signs [b]	X	X	X	X	X	X	X	X	X	X	X
12-lead ECG	X										X
Serum pregnancy test (females) [c]	X	X									X
Urine drug screen	X	X									
Alcohol breath test	X	X									
Serology/virology [c]	X										
Clinical chemistry [c]	X	X [d]		X [e]							X
Haematology [c]	X	X [d]		X [e]							X
Urinalysis [c]	X	X [d]		X [e]							X
Enema retainment test (30 min) [f]	X										
Menstrual history check (females) [g]	X	X									
Enema retainment test (8 h) [h]		X									
Admission to trial site		X									
Standardised meals		X	X	X	X	X	X	X	X	X	X
Fasting and water abstain [i]		X	X	X	X	X	X	X	X		
Evacuation of bowel [j]		X	X	X	X	X	X	X	X		
Evacuation of bladder [k]		X	X	X	X	X	X	X	X		
Adult diaper application and weight measurement [l]		X	X	X	X	X	X	X	X		
(N)IMP weighting [m]		X	X	X	X	X	X	X	X		
Administration of IMP [n]			X	X	X	X	X	X	X		
PK blood sampling [o]			X	X			X	X	X	X	
Urine sampling [p]			X						X	X	
IMP retainment [q]			X	X	X	X	X	X	X		
Bedpan application [r]		X	X	X	X	X	X	X	X		
Defecation and time record [s]			X	X	X	X	X	X	X	X	
Adverse events	X [t]	X	X	X	X	X	X	X	X	X	X
Prior/concomitant medication	X	X	X	X	X	X	X	X	X	X	X
Discharge from trial site [u]											X

[a] Body height will not be measured on End-of-trial examination.

[b] Vital signs (blood pressure, pulse rate, axillary temperature and respiratory rate) will be measured after 5 minutes resting during Day -14 to -2, on Day -1, on each day of Treatment Period (Day 1 to 8), and as part of the End-of-trial examination, between 8-10 a.m. on each day. Blood pressure will be measured in a supine position.

[c] Analyses performed in local laboratory.

[d] The tests will not be performed on Day -1 if they were performed within 7 days before Day -1 (i.e. between Day -8 to Day -2).

[e] Blood and urine samples will be taken before 2nd dose.

[f] Enema retainment test (30 min, performed during Day -14 to -2) must be done prior to DRE. A placebo enema (100 mL) will be administered to subjects. The placebo enema is pre-heated to 30~37°C, and dosed in the same way as IMP administration. A diaper, which has been weighted, will be applied to a subject. The diaper will be weighted again after 30 minutes post placebo enema administration. The diaper should be weighted no more than 10 minutes following being taken off. Eligible subjects should retain the enema for at least 30 minutes with no more than 20 g diaper weight increase.

[g] Menstrual history (females) will be checked during Day -14 to -2 and on Day -1. The qualified female subjects' menstruation will not likely overlap Treatment Period.

[h] An enema retainment test (8 h) will be performed on Day -1. A placebo enema (100 mL) will be administered to subjects at least 22 h prior to 1st dose of IMP administration. All the procedures associated with IMP compliance improvement and evaluation, including fasting and water abstain [i], evacuation of bowel [j], evacuation of bladder [k], adult diaper application and weight measurement [l], (N)IMP weighting [m], (N)IMP retainment [p], bedpan application [q], and defecation and time record [r], are the same as IMP administration. Eligible subjects should retain the enema for at least 8 hours with less than or equivalent to 20 g diaper weight increase.

[i] Subjects will be fasted and abstained to water 2 h pre-dose until at least 8 h post-dose.

[j] Water enema will be used to subjects approximately 2 h before NIMP administration on Day -1, 1st and 7th IMP doses to facilitate bowel evacuation. Approximately 2 h before 2nd to 6th IMP administrations, subjects will be required to go to toilet and empty their bowls as clean as possible, but water enema will not be used.

[k] A visit to toilet to evacuate bladder is required approximately 10 min before NIMP administration (Day -1) and before each IMP administration (Day 1 to 7).

[l] Adult diaper will be used to evaluate the compliance of (N)IMP retainment. New (unused) diaper weight will be measured and recorded, and afterwards the diaper will be applied to a subject. After 8 h post-dose, the diaper will be weighed again. The diaper should be weighted no more than 10 minutes following being taken off.

[m] After (N)IMP is pre-heated to 30~37°C inside its foil pack, the (N)IMP will be wiped dry and weighted without foil pack. After (N)IMP administration, the weight of used (N)IMP will be weighted again. The weight of unused and used (N)IMP will be recorded.

[n] IMP is administered approximately 9-10 p.m. on Day 1-7.

[o] Blood samples for analysis of mesalazine will be collected repeatedly up to 24 hours following 1st, 5th and 7th doses. The sampling times associated with 1st, 5th and 7th doses are: immediately pre-dose and 0.5, 1, 2, 3, 4, 5, 6, 7, 8, 10, 12, 14, 16, 20 and 24 h post-dose (before the next dose).

[p] For drug excretion study, urine will be sampled after the 7th dose, within the intervals of 0-12 h and 12-24 h post-7th dose. Blank urine will also be sampled prior to 1st dose.

[q] IMP should be retained at least 8 h post-dose.

[r] Subjects who urinate within 8 h post-dose will use bedpans in bed.

[s] After diaper weight is measured, subjects will be required to visit to toilet during 8-10 h post-dose and the defecation time (as time interval) will be recorded.

[t] Start to collect AE since the first visit.

[u] Subjects will only be discharged from the trial site if results of End-of-trial examination are considered 'normal' or 'abnormal, not clinically significant'. In case any indicator is considered 'abnormal, clinically significant' at this time, additional managements will be taken at the discretion of the Investigator before discharging of the subjects.

TABLE OF CONTENTS

SYNOPSIS **2**

LIST OF TABLES **16**

LIST OF FIGURES **16**

LIST OF ABBREVIATIONS AND DEFINITION OF TERMS **17**

1 INTRODUCTION **20**
1.1 Background 20
1.2 Scientific Justification for Conducting the Trial 21
1.3 Benefit / Risk Aspects 21

2 TRIAL OBJECTIVES AND ENDPOINTS **23**
2.1 Objectives 23
2.2 Endpoints 23

3 INVESTIGATIONAL PLAN **24**
3.1 Overall Trial Design 24
3.1.1 Trial Design Diagram 24
3.1.2 Overall Design and Control Methods 25
3.1.3 Trial Schedule 27
3.2 Planned Number of Trial Sites and Subjects 27
3.3 Interim Analysis 27
3.4 Data Monitoring Committee (DMC) 27
3.5 Discussion of Overall Trial Design and Choice of Control Groups 27
3.5.1 Trial Design 27
3.5.2 Selection of Endpoints 28
3.5.3 Blinding 29
3.5.4 Selection of Doses in the Trial 29
3.5.5 Selection and Timing of Dose for Each Subject 29
3.5.6 Selection of the Trial Population 29
3.5.7 Withdrawal Criteria 29
3.5.8 Follow-up Procedures 30

4 SELECTION OF TRIAL POPULATION **31**
4.1 Trial Population 31
4.1.1 Inclusion Criteria 31
4.1.2 Exclusion Criteria 32
4.2 Method of Assigning Subjects to Treatment Groups 33
4.2.1 Recruitment 33
4.3 Restrictions 33
4.3.1 Prior and Concomitant Therapies 33
4.3.2 Prohibited Therapy 33
4.3.3 Other Restrictions 34
4.4 Withdrawal Criteria 34

5 TREATMENTS **36**
5.1 Treatments Administered 36

5.1.1 Investigational Medicinal Product (IMP) 36
5.1.2 Non-Investigational Medicinal Product (NIMP) 36
5.2 Characteristics and Source of Supply 36
5.2.1 Investigational Medicinal Product 37
5.2.2 Non-Investigational Medicinal Product 37
5.3 Packaging and Labelling 37
5.3.1 Investigational Medicinal Product 37
5.3.2 Non-Investigational Medicinal Product 37
5.4 Conditions for Storage and Use 38
5.4.1 Investigational Medicinal Product 38
5.4.2 Non-Investigational Medicinal Product 38
5.5 Blinding / Unblinding 38
5.6 Treatment Compliance 38
5.6.1 Dispensing and Accountability 38
5.6.2 Assessment of Enema Retention 38
5.7 Return and Destruction of Medicinal Products 39

6 TRIAL PROCEDURES 40
6.1 Trial Flow Chart 40
6.2 Screening 42
6.3 Treatment Period 43
6.4 End-of-trial Examination 44

7 TRIAL ASSESSMENTS 46
7.1 Assessments Related to Endpoints 46
7.1.1 Pharmacokinetic Assessments 46
7.1.2 Vital Signs 46
7.1.3 Electrocardiogram 47
7.1.4 Adverse Events 47
7.1.5 Clinical Chemistry 47
7.1.6 Haematology 48
7.1.7 Urinalysis 50
7.1.8 Physical Examination 51
7.2 Other Assessments 51
7.2.1 Digital Rectal Examination 51
7.2.2 Stool test 52
7.2.3 Serology and Virology 52
7.2.4 Serum Pregnancy Test (females) 52
7.2.5 Urine Drug Screen and Alcohol Breath Test 52
7.2.6 Demographic and Baseline Data 53
7.2.7 Medical history 53
7.2.8 Prior and Concomitant Medication 53
7.2.9 Enema Retainment Test (30 min) 53
7.2.10 Enema Retainment Test (8 h) 54
7.3 Drug Concentration Measurements 54
7.4 Handling of Biological Samples 54

8 ADVERSE EVENTS 56
8.1 Adverse Event Definition 56
8.2 Collection and Recording of Adverse Events 56
8.2.1 Collection of Adverse Events 56
8.2.2 Recording of Adverse Events 57
8.3 Pregnancy and Pregnancy Outcome 59
8.4 Serious Adverse Events 60
8.4.1 Serious Adverse Event Definition 60
8.4.2 Collection, Recording and Reporting of Serious Adverse Events 61
8.5 Follow-up of Adverse Events and Serious Adverse Events 62
8.5.1 Follow-up of Adverse Events with Onset during the Trial 62
8.5.2 Collection of Serious Adverse Events with Onset after Last Trial 63

9 STATISTICAL METHODS 64
9.1 Determination of Sample Size 64
9.2 Subject Disposition 64
9.3 Protocol Deviations 64
9.4 Analysis Sets 64
9.4.1 Intention-to-Treat (ITT) Analysis Dataset 64
9.4.2 Full Analysis Set (FAS) 65
9.4.3 Per Protocol (PP) Dataset 65
9.4.4 Safety Dataset 65
9.5 Trial Population 65
9.5.1 Demographics and other Baseline Characteristics 65
9.5.2 Medical History, Concomitant Medication and Other Safety Evaluations 65
9.5.3 Treatment compliance 65
9.6 Pharmacokinetic Endpoint Assessments 65
9.6.1 General Considerations 66
9.6.2 Primary Pharmacokinetic Endpoints 66
9.6.3 Secondary Pharmacokinetic Endpoints 67
9.7 Treatment Compliance 67
9.8 Safety 67
9.8.1 General Considerations 67
9.8.2 Adverse Events 67
9.8.3 Safety Laboratory Variables 68
9.8.4 Vital Signs 69
9.8.5 ECG 69
9.8.6 Physical Examination 69
9.9 Interim Analyses 69

10 DATA HANDLING 70
10.1 Source Data and Source Documents 70
10.2 CRF 70
10.3 Data Management 71
10.4 Provision of Additional Information 71

11 MONITORING PROCEDURES 72
11.1 Periodic Monitoring 72

11.2 Audit and Inspection ... 72
11.3 Confidentiality of Subject Data ... 72

12 CHANGES IN THE CONDUCT OF THE TRIAL ... 74
12.1 Protocol Amendments ... 74
12.2 Deviations from the Protocol ... 74
12.3 Premature Trial Termination ... 74

13 REPORTING AND PUBLICATION ... 75
13.1 Clinical Trial Report ... 75
13.2 Confidentiality and Ownership of Trial Data ... 75
13.3 Publications and Public Disclosure ... 75
13.3.1 Publication Policy ... 75
13.3.2 Public Disclosure Policy ... 75

14 ETHICAL AND REGULATORY ASPECTS ... 76
14.1 Ethics Committee (EC) ... 76
14.2 Regulatory Authority(ies) Authorisation / Approval / Notification ... 76
14.3 End-of-Trial and End-of-Trial Notification ... 76
14.4 Ethical Conduct of the Trial ... 76
14.5 Subject Information and Consent ... 76
14.6 Subject Information Card ... 77
14.7 Compliance Reference Documents ... 77

15 LIABILITIES AND INSURANCE ... 78
15.1 ICH-GCP Responsibilities ... 78
15.2 Liabilities and Insurance ... 78

16 ARCHIVING ... 79
16.1 Investigator File ... 79
16.2 Trial Master File ... 79

17 REFERENCES ... 80

APPENDICES ... 81
Appendix 1 Instroduction to IMP Administration ... 82
Appendix 2 Introduction to Bowel Cleaning Using Water Enema ... 83

LIST OF TABLES

Table 1 Trial Flow Chart 10
Table 2 Trial Design Diagram 24
Table 3 Safety laboratory parameters - Clinical chemistry 48
Table 4 Safety laboratory parameters - Haematology 50
Table 5 Safety laboratory parameters - Urinalysis 51

LIST OF FIGURES

Figure 1 Trial Scheme 41
Figure 2 Procedures associated with IMP compliance improvement and assessment 43

LIST OF ABBREVIATIONS AND DEFINITION OF TERMS

List of Abbreviations

ADR	Adverse drug reaction
AE	Adverse Event
ATC	Anatomical Therapeutic Code
BMI	Body mass index
CD	Crohn's disease
CFDA	China Food and Drug Administration
CRF	Case Report Form
CRO	Contract Research Organisation
DMC	Data Monitoring Committee
DRE	Digital rectal examination
ECG	Electrocardiogram
EC	Ethics Committee
EU	European Union
FAS	Full Analysis Set
GCP	Good Clinical Practice
GMP	Good Manufacturing Practice
IBD	Inflammatory bowel disease
ICD	Informed Consent Documents
ICH	International Conference on Harmonisation
INN	International non-proprietary name
IEC	Independent Ethics Committee
IMP	Investigational Medicinal Product
IND	Investigational New Drug (Application)
ITT	Intention-to-Treat
LLQ	Lower limit of quantification
MedDRA	Medical Dictionary for Regulatory Activities
ND	Not detectable
NIMP	Non-Investigational Medicinal Product
PK	Pharmacokinetic(s)
PP	Per Protocol

SAE	Serious Adverse Event
SDV	Source Data Verification
SOC	System organ class
SUSAR	Suspected Unexpected Serious Adverse Reaction
UC	Ulcerative colitis
WHO	World Health Organisation

Definition of Terms

Enrolment	A subject is enrolled in the trial when the Informed Consent is signed.
Trial duration	Time from signing informed consent to discharge from trial site.
Treatment Period	Time from first dosing with investigational medicinal product (IMP) to the last PK blood sampling visit.
End of trial	The last contact with the last subject undergoing the trial.

PHARMACOKINETIC TERMS

NCA — Non-compartmental analysis

λ_z — First-order rate constant associated with the terminal (log-linear) portion of the plasma concentration-time curve estimated via linear regression of the time versus log concentration.

AUC — Area under the plasma concentration-time curve to infinity according to the following formula:

$$AUC = AUC_t + \frac{C_{last}}{\lambda_z}$$

where C_{last} is the last measurable plasma concentration.

AUC_t — Area under the plasma concentration-time curve from time zero up to time t, where t is the last time point at which the subject shows concentration above the lower limit of quantification (LLQ; time of last measurable (non-zero) concentration at t_{last}). AUC_t is calculated using the linear trapezoidal (linear interpolation) method according to following formula:

$$AUC\Big|_{t_1}^{t_2} = \delta t \times \frac{C_1 + C_2}{2}$$

where C_1 is the plasma concentration at time point one (t_1), C_2 is the plasma concentration at time point two (t_2) and δt is (t_2-t_1).

F — Bioavailability.

CL	Clearance.
CL/F	Apparent oral clearance = Dose/AUC.
C_{max}	Maximum plasma concentration observed.
t_{max}	Time of C_{max} after oral administration.
$t_{½}$	Terminal elimination half-life.
V_z/F	Apparent volume of distribution = $\lambda_z \cdot$Dose/AUC.
C_{avg}	Average plasma concentration at multiple dose period=AUC_{tau}/ time tau.
Ae	Total cumulative amount of mesalazine and N-acetyl-mesalazine excreted in the urine

1 INTRODUCTION

1.1 Background

Ulcerative colitis (UC), a form of inflammatory bowel disease (IBD), is a chronic inflammatory bowel disease of complex etiology and pathogenesis, and characterized by a continuous inflammation of the colonic mucosa starting from the rectum. The extent of the disease can vary from proctitis to left-sided colitis and extensive colitis or pancolitis. Ulcerative colitis is slightly more common in women than in men. Age of onset shows a bimodal pattern, with a peak at 15-25 years and a smaller peak at 55-65 years, although the disease can occur at any age [1]. The annual incidence of UC per 100,000 people is 10.4-12 cases in the US and 8 cases in Europe. The prevalence rate per 100,000 people is 35-100 cases in the US and 21-243 cases in Europe. There are some data showing the number of UC cases have been increasing during the past 2 decades in China, although the population-based incidence of UC was studied less extensively. A recent epidemiologic study conducted in Wuhan defined the annual incidence rate as 1.45 per 100,000 in this region of China [2]. And based on multi-hospital case calculation in China, the prevalence of UC in China is at least 11.6 cases per 100,000 people [3].

Active episodes of UC are marked by passing of blood and mucus with associated, diarrhea, and abdominal pain presenting as urgency, frequency and a feeling of incomplete evacuation (tenesmus) [4]. In the most severe forms, systemic signs comprising fever, anorexia and weight loss may occur. Approximately 60% of patients have a mild form, 25% a moderate form and 15% a severe disease. It is estimated that approximately 1 of every 2 patients with UC will experience a relapse within 1 year, and the cumulative probability of relapse is 80% within 2 years and 95% within 10 years [5].

Mesalazine is the main first-line therapy for induction of remission and maintenance therapy of mild to moderate active UC [6]. The therapeutic value of mesalazine after oral as well as rectal administration appears to be due to local effect on the inflamed intestinal tissue, rather than to systemic effect [7]. Increased leucocyte migration, abnormal cytokine production, increased production of arachidonic acid metabolites, particularly leukotriene B_4, and increased free radical formation in the inflamed intestinal tissue are all present in patients with IBD. Mesalazine has *in vitro* and *in vivo* pharmacological effects that inhibit leucocyte chemotaxis, decrease cytokine and leukotriene production, and scavenge for free radicals [8-11]. It is currently unknown which, if any, of these mechanisms play a predominant role in the clinical efficacy of mesalazine.

Ferring medicinal products of mesalazine, which brand name is PENTASA®, were first approved in Denmark in 1986 as PENTASA® prolonged release tablets 250 mg and PENTASA® rectal suspension 1 g/100 mL (enema). The products were launched shortly thereafter, and in the following years, several line extensions were approved: PENTASA® prolonged release tablets 500 mg, PENTASA® suppositories 1 g, and PENTASA® prolonged release granules 1 g and 2 g. As of February 2014, PENTASA® rectal suspension had been approved in 71 countries.

Three clinical pharmacokinetic studies have been completed using PENTASA® enema. Scintigraphic imaging study at four hours post 1g/100 mL mesalazine enema dose showed that in healthy male subjects, 72% of the suspension remained in the rectum and sigmoid colon, 25% had spread to the descending colon and 3% had travelled as far as the transverse colon [12]. The other two trials, both of which involved plasma pharmacokinetic study and urine drug recovery study, demonstrated a low systemic bioavailability of mesalazine following rectal administration. Based on urine recoveries under steady-state conditions, approximately 15-20% is absorbed after administration of rectal suspensions (enemas) in healthy subjects [7, 13]. However, the half-life of mesalazine was not determined following rectal suspension administration in these studies, while half-life of mesalazine following intravenous administration was determined at 0.5~1 h in other studies [14, 15].

1.2 Scientific Justification for Conducting the Trial

This study is part of the clinical development program for PENTASA® enema in China, and is designed to determine the single and multiple dose pharmacokinetics (PK) and safety and of PENTASA® enema in healthy Chinese subjects.

Although PENTASA® enema is considered as a topical therapy, the systemic bioavailability is not negligible. Studies in healthy subjects demonstrated a bioavailability of 15-20% of PENTASA® enema. However, the systemic exposure of mesalazine after rectal suspension administration has not been known in Chinese population. This study will determine single and multiple dose pharmacokinetics of mesalazine enema (PENTASA®, 1 g/100 mL) after rectal administration, and demonstrate the safety (e.g. drug accumulation after multiple dose) in healthy Chinese subjects.

1.3 Benefit / Risk Aspects

An extensive safety profile has been established since PENTASA® was first approved in 1986. In China, the PENTASA® tablets and suppository have been marketed since 1993, and they demonstrated similar safety and tolerability in Chinese patients as well.

As of 28 February 2013, based on product sales, the cumulative patient exposure for both oral and rectal formulations is estimated to be 8,036,415 patient-years worldwide, and the cumulative patient exposure for PENTASA® rectal formulations is estimated to be 432,533 worldwide. Estimates of exposure assume a daily dose of 1.5 g for oral formulations and either 1 suppository or 1 enema per day, regardless of strength, and 100% consumption of the purchased product. The number of patients exposed in clinical trials up to 28 Feb 2013 is approximately 10,600, including patients with ulcerative colitis (UC) and patients with Crohn's disease (CD) (CD is another indication of mesalazine).

Since the current study involves healthy subjects, there are no direct health benefits from participating. The study will reveal the physiological disposition of mesalazine enema in Chinese population.

PENTASA® has been marketed for more than 25 years. The common (⩾1% and <10%) AEs based on clinical studies and post-marketing surveillance with PENTASA® (all formulations) are headache, diarrhoea, abdominal pain, nausea, vomiting and rash (including urticaria, erythematous rash). All these AEs are mild.

Hypersensitivity reactions and drug fever may occasionally occur. Following rectal administration, local reactions such as pruritus, rectal discomfort, and urge may occur.

It is possible that the healthy subjects choosing to participate in this trial could experience any of the above identified events, or events that have not been reported previously. However, the possibility of clinically significant events occurring in subjects participating in the study for any of these risks is deemed low and ethically justifiable. Since there will be potential risks from participation, potential subjects for this trial will be fully informed of the possible risks from participation during the informed consent process (i.e. through reading the Informed Consent Documents [ICD] and in discussion with the Investigator) to allow them to reach an individual decision on whether they want to participate.

2 TRIAL OBJECTIVES AND ENDPOINTS

2.1 Objectives

Primary Objectives

To determine single and multiple dose pharmacokinetics of mesalazine (PENTASA®, 1 g/100 mL) after rectal administration in healthy Chinese subjects.

Secondary Objectives

To assess the safety of single and multiple dose of PENTASA® enema after rectal administration in healthy Chinese subjects.

To determine total cumulative amount of mesalazine and N-acetyl-mesalazine excreted in urine

2.2 Endpoints

Primary Endpoints

- Pharmacokinetics:
  - Single dose period (following the 1st dose): AUC_t, C_{max}, t_{max} of mesalazine.
  - Multiple dose period (following the 5th dose): AUC_{tau}, C_{max}, t_{max} of mesalazine
  - Multiple dose period (following the 7th dose): AUC_{tau}, C_{max}, t_{max} of mesalazine.

Secondary Endpoints

- Pharmacokinetics:
  - Single dose period (following the 1st dose): AUC, CL/F, V_z/F, λ_z and $t_{½}$ of mesalazine.
  - Multiple dose period (following the 5th dose): AUC, CL/F, V_z/F, C_{avg}, λ_z and $t_{½}$ of mesalazine.
  - Multiple dose period (following the 7th dose): AUC, CL/F, V_z/F, C_{avg}, λ_z and $t_{½}$ of mesalazine.
  - Total cumulative amount of mesalazine and N-acetyl-mesalazine excreted in the urine (Ae) up to 24 h following the 7th dose of mesalazine.
- Type, frequency and intensity of adverse events.
- Clinically significant changes in vital signs, 12-lead ECG, clinical chemistry, haematology, and urinalysis.

3 INVESTIGATIONAL PLAN

3.1 Overall Trial Design

3.1.1 Trial Design Diagram

Trial design diagram is shown in Table 2.

Table 2 Trial Design Diagram

Screening	Treatment Period	End-of-trial examination
Inclusion/exclusion	IMP Administration/ Assessments	Assessments
Day -14 to -1*	Day 1 to 7/Day 1 to 8	Day 9

* *Note*: There is no Day 0 in the study.

3.1.2 Overall Design and Control Methods

This is an open-label, single and multiple dose study in healthy Chinese subjects.

Subjects will be administered a daily dose of IMP for 7 days. Single dose PK will be studied after 1st dose. Multiple dose PK will be studied after 5th and 7th dose.

Three study visits are planned:

- Screening occurring between Day -14 to Day -1 before the 1st dose of IMP.
- One residential visit consisting 8 days (Day 1 to Day 8), during which period IMP will be dosed and blood and urine will be sampled.
- An End-of-trial examination visit, performed on Day 9.

After signing Informed Consent Documents (ICD) during Day -14 to Day -2 at Screening, all subjects will undergo a general physical examination, vital signs, 12-lead ECG, digital rectal examination, stool test, serum pregnancy test (females), serology/virology, clinical chemistry, haematology, urinalysis, urine drug screen and alcohol breath test. Enema retainment test (30 min) will be performed to exclude subjects who are sensitive to rectal administration (enema retainment test must be done prior to DRE). A placebo enema (100 mL) will be administered to subjects. The placebo enema is pre-heated to 30~37°C, and dosed in the same way as IMP administration. A diaper, which has been weighted, will be applied to a subject. The diaper will be weighted again after 30 minutes post placebo enema administration. The diaper should be weighted no more than 10 minutes following being taken off. Eligible subjects should retain the enema for at least 30 minutes with less than or equivalent to 20 g diaper weight increase. Menstrual history (females) will be checked. The qualified female subjects' menstruation will not likely overlap Treatment Period (Day 1 to 8). Inclusion/Exclusion criteria will be checked based on available information of subjects during Day -14 to -2.

On Day -1, subjects will undergo an inclusion/exclusion criteria check including vital signs, serum pregnancy test (females), urine drug screen, breath alcohol test, clinical chemistry, haematology, urinalysis and menstrual history (females). However, clinical chemistry, haematology, urinalysis will not be performed if they were performed within 7 days before Day -1 (i.e. between Day -8 to Day -2). On Day -1, an enema retainment test (8 h) will be performed. (Water enema will be used to clean the bowel approximated 2 h prior to the test.) A placebo enema (100 mL) will be administered to subjects at least 22 h prior to 1st dose of IMP administration. A diaper, which has been weighted, will be applied to a subject. The diaper will be weighted again after 8 h post placebo enema administration. The diaper should be weighted no more than 10 minutes following being taken off. Eligible subjects should retain the enema for at least 8 h with less than or equivalent to 20 g diaper weight increase. Eligible subjects will be considered as successfully screened and admitted to the IMP dosing.

At Treatment Period (Day 1 to 8), subjects will receive a daily rectal dose of PENTASA® enema (1 g/100 mL) 9-10 p.m. on Day 1-7 and be required to retain the enema for at least 8 h after IMP dosing. The following procedures will be conducted to improve and evaluate the compliance of IMP retainment:

- Subjects will be fasted and abstained to water 2 h pre-dose (i.e. 7-8 p.m.) until 8 h post-dose (i.e. 5-6 a.m. in the morning of next day).
- Approximately 2 h before each dose, subjects will be required to go to toilet and empty their bowels as clean as possible. Water enema will be used to facilitate bowel evacuation before 1st and 7th doses, while it will not be used before 2nd-6th doses.
- A visit to toilet to evacuate bladder is required approximately 10 min before each dose.
- After IMP is pre-heated to 30~37°C inside its foil pack, the IMP will be wiped dry and weighted without foil pack. After IMP administration, the weight of used IMP will be weighted again. The weight of unused and used IMP will be recorded.
- Adult diaper will be used to evaluate the compliance of IMP retainment. The weight of a new (unused) diaper will be measured and recorded, and afterwards the diaper will be applied to a subject. After 8 h post-dose, the diaper will be weighed again. The diaper should be weighted no more than 10 minutes following being taken off. If diaper weight increases more than 20 g after 1st or 7th dose, or if diaper weight increases more than 50 g after one of 2nd to 6th dose, the corresponding subject will be withdrawn from the trial.
- Subjects who urinate within 8 h post-dose will use bedpans in bed.
- After diaper weight is measured, subjects will be required to visit to toilet during 8-10 h post-dose and the defecation time (as time interval) will be recorded.

Blood samples for analysis of mesalazine will be collected repeatedly up to 24 h following 1st, 5th and 7th doses. The sampling times associated with each of the three doses are: immediately prior to dosing and 0.5, 1, 2, 3, 4, 5, 6, 7, 8, 10, 12, 14, 16, 20 and 24 h post-dose (before the next dose). Approximately 4 mL blood will be sampled at each time point.

Urine samples will be collected for the determination of mesalazine and its main metabolite, N-acetyl-mesalazine. 5~10 mL blank urine will be collected prior to the 1st dose and all urine during intervals of 0-12 h and 12-24 h following the 7th doses will be collected.

Vital signs will be measured on each day from Day 1 to Day 8. Clinical chemistry, haematology, and urinalysis will be measured on Day 2.

On Day 9, subjects will undergo an End-of-trial examination including physical examination (excluding height), vital signs, 12-lead ECG, serum pregnancy test (females), clinical chemistry, haematology, and urinalysis. Subjects will only be discharged from the trial site if results of End-of-trial examination are considered 'normal' or 'abnormal, not clinically significant'. In case any indicator is considered 'abnormal, clinically significant' at this time, additional managements will be taken at the discretion of the Investigator before discharging of the subjects.

The duration of residential stay is from Day -1 to Day 9. Standardised meals will be provided during Day -1 to Day 9.

Approximately total 260 mL will be sampled from each subject 200 mL for PK blood sampling and 60 mL for safety tests).

No concomitant medication except necessary for AEs is allowed during the study period. The medication used for the treatment of AEs will be recorded.

3.1.3 Trial Schedule

Screening is planned to start in June 2015. IMP dosing and PK blood and urine sampling is planned between June to September in 2015. Last Subject Last Visit is planned in September 2015.

3.2 Planned Number of Trial Sites and Subjects

The study will be conducted at a single Phase I unit in China. 10 eligible subjects of both sexes will be admitted to the trial to achieve 8 completers, in which at least 2 completers of each sex.

Subjects who do not complete the study may be replaced to achieve at least 8 completers.

3.3 Interim Analysis

No formal statistical interim analysis is planned.

3.4 Data Monitoring Committee (DMC)

There will be no Data Monitoring Committee in this study.

3.5 Discussion of Overall Trial Design

3.5.1 Trial Design

The current study design is an open-label, single and multiple dose study in healthy Chinese subjects.

Since it is a PK study of IMP, there will be no control and hence it will be open-label. Healthy Chinese subjects are selected as trial population in order to reflect the nature of IMP, which is the general requirement by China Food and Drug Administration (CFDA) [16].

Only one dose level is selected (see Section 3.5.4 for more information) to reflect the dose level for clinical application. PK after single (the 1st) and the 7th dose will be studied, and PK parameters (AUC after 1st dose and AUC_{tau} after the 7th dose) will be compared in order to reveal potential drug accumulation after multiple dose.

The systemic exposure following rectal administration of mesalazine is low, and depends on the dose, the formulation, and the extent of spread. 8 h is considered as the general duration of IMP

retainment in clinical practice. The PK blood sampling will last for 24 h for each of the three doses, to capture the plasma mesalazine concentration-time profiles during a dose interval.

The primary objective of this study is to determine single and multiple dose pharmacokinetics of mesalazine (PENTASA®, 1 g/100 mL) after rectal administration in healthy Chinese subjects. PK parameters (i.e. AUC) for single (1st) and multiple (7th) dose will compared to evaluate drug accumulation after multiple dose. Therefore, IMP administration related procedures should be identical for 1st and 7th dose, and factors (other than dose times) that may impact on mesalazine absorption should also be diminished or identical for both 1st and 7th dose. Hence, in order to avoid potential effect of intestinal content on mesalazine absorption, bowels will be cleaned using water enema before 1st and 7th dose. In clinical practise, a visit to toilet is recommended before PENTASA® enema administration. In this study, a visit to toilet is required before 2nd to 6th IMP doses (and NIMP dose on Day -1) but water enema will not be used. The drug accumulation in the body (if any) caused by 2nd to 6th doses should reflect its accumulation in clinical practise, as same pre-administration procedure (i.e. visit to toilet) is used in clinical practise and in this trial.

However, one concern regarding water enema application is that the procedure is not completely the same with clinical practise. Although the PK profiles obtained following 1st and 7th doses reflect maximum absorption potential in clinical practise, the PK profiles under real condition in clinical practise, i.e. bowels may be filled with intestinal content, cannot be studied after 1st or 7th dose. Therefore, PK profile after the 5th dose, before which dose water enema will not be used, will be also studied.

Since retaining time of enema likely affect systemic exposure of mesalazine, it is pivotal for this trial that the subjects are able to retain the enema during required period, i.e. 8 h. Therefore, enema retainment tests with required enema retaining time 30 minutes and 8 hours will be performed during Day -14 to -2 and on Day -1, respectively. The subjects will be dosed with placebo enema, which is identical in appearance with Pentasa® enema but does not contain active component, mesalazine. The 30 minutes test is a pre-screening in order to exclude subjects who are sensitive to rectal administration. The 8 h test is used to exclude subjects who are unable to retain enema for 8 hours. Meanwhile, potential subjects should be aware the discomfort caused by enema administration and hence decide whether to continue to participate in the trial.

It is also necessary to evaluate the compliance of IMP retainment. Adult diaper will be used to evaluate the compliance of IMP retainment. A new diaper will be applied to a subject prior to each IMP dosing, and the weight of the diaper will be measured pre-dose and 8 h post-dose. In order to avoid weight loss caused by evaporation, diaper should be weighted no more than 10 minutes following being taken off.

3.5.2 Selection of Endpoints

The primary endpoint is based on standard PK parameters for both single and multiple dose studies.

3.5.3 Blinding

As this study is open-label, no blinding will take place.

3.5.4 Selection of Doses in the Trial

The IMP is a rectal suspension of mesalazine, 1 g in 100 mL aqueous acetate buffer. It is a topical therapy administered once daily. The dose of IMP is not likely to be adjusted in clinical practice. Therefore, only one dose level at 1 g/100 mL is selected.

3.5.5 Selection and Timing of Dose for Each Subject

All subjects will be administered a fixed dose of 1 g/100 mL. IMP will be administered at bedtime (9-10 pm) as approximately the same time as clinical application. The administration at bedtime also facilitates IMP retainment mostly because subjects will lie in bed following administration.

IMP will be administered once daily for 7 days, in order to achieve steady state (if steady state can be achieved after multiple doses administered), at which condition the PK will be studied. Around 10 subjects will be exposed to IMP from Day 1 to Day 7.

Subjects will be fasted and abstained to water 2 h pre-IMP dosing and 8 h post-IMP dosing. This restriction will minimize the activities of subjects, i.e. going to toilet after IMP administration, and in turn to minimize the potential of enema flow-out.

3.5.6 Selection of the Trial Population

The trial population is selected to represent healthy Chinese subjects of both genders.

The IMP is dosed rectally and is spread from rectum to splenic flexure, in which part of gastrointestinal tract mesalazine is absorbed into systemic circulation. Therefore subjects with healthy gastrointestinal status will be recruited. Subjects who has presence or a history of clinically significant diseases of gastrointestinal diseases, who has a history of gastrointestinal surgery, or who has diseases of the ano-rectum, including fistulas, anatomic irregularities, rectal tone compromising the ability to hold the enema will be excluded. Recruited subjects should be in healthy anorectal status, confirmed by medical history, digital rectal examination and stool test performed at Screening. Recruited subjects should also pass the enema retainment test at Screening.

3.5.7 Withdrawal Criteria

The subjects who are considered non-compliant of IMP administration and retainment should be withdrawn from the trial. In this study, IMP retainment compliance is defined as ⩾80% enema retained after 8 h post 1st and 7th dose, and ⩾50% enema retained after 8 h post 2nd to 6th dose. Adult diaper will be used to evaluate the compliance of IMP retainment. The weight of a new (unused) diaper will be measured and recorded, and afterwards the diaper will be applied to a subject. After 8 h post-dose, the diaper will be weighed again. If diaper weight increases more than

20 g after 1st or 7th dose, or if diaper weight increases more than 50 g after one of 2nd to 6th dose, the corresponding subject will be withdrawn from the trial.

Females whose menstruation likely overlaps the planned Treatment Period (Day 1 to 8) will be excluded from the trial population, for the reason that menstruation makes it impossible to evaluate IMP retainment compliance using diaper weight gain method. However, in the case that female subjects starting menses during treatment period (Day 1 to 8), she should be withdrawn from the trial in the following situation:

- Menstruation starts before 7th dose of IMP administration (i.e. 9-10 p.m. on Day 7).
- Menstruation starts after 7th dose of IMP administration and the 7th diaper weight increases more than 20 g.

3.5.8 Follow-up Procedures

No follow-up procedure is planned since the systemic exposure of mesalazine is estimated negligible on Day 9.

4 SELECTION OF TRIAL POPULATION

4.1 Trial Population

Chinese subjects who meet the inclusion criteria and do not meet exclusion criteria

4.1.1 Inclusion Criteria

1. Signed written Informed Consent Document(s) (ICD)
2. 18 - 45 years of age. The subjects must be at least 18 years (including the 18th birthday) and no more than 45 years (up to the day before the 46th birthday) when they sign the ICD
3. Body weight ⩾ 50 kg (male) or ⩾45 kg (female) and body mass index (BMI) of 19-24 kg/m2 (both inclusive)
4. Healthy according to medical history, physical examination, 12-lead ECG, vital signs, and laboratory profile of blood and urine
5. Healthy rectal and anal status based on digital rectal examination and stool test
6. Healthy intestinal track status according to medical history with normal defecation frequency (once daily to three times per week during past 3 months)
7. Capable to retain a placebo enema for at least 30 minutes during Day -14 to -2, with diaper weight increase no more than 20 g
8. Capable to retain a placebo enema for at least 8 hours on Day -1, with diaper weight increase no more than 20 g
9. Negative virology/serology for human immunodeficiency virus antibodies (HIV-Ab), hepatitis B surface antigen (HBs-Ag), hepatitis C virus antibodies (HCV-Ab), and treponema pallidum antibodies (TP-Ab) at Screening
10. Negative urine drug screen and alcohol breath test
11. Agrees to use a barrier contraception method (e.g. condom) from signing the ICD to 3 days after End-of-trial examination
12. Non-smoker or light smoker (less than five cigarettes, or equivalent, per day). In the latter case, the subject must abstain from smoking during the residential stay at trial site
13. For female subjects: a) history of normal menstrual cycles with a mean length of 28-35 days within 6 months prior to Screening, b) history of normal menstruation of 3-7 days within 6 months prior to Screening and, c) end-of-menstruation day is within 7 days prior to Day -1

4.1.2 Exclusion Criteria

1. Presence or a history of clinically significant diseases of the renal, hepatic, gastrointestinal, cardiovascular, musculoskeletal systems or presence or history of clinically significant psychiatric, immunological, endocrine or metabolic diseases

2. Cancer within the last 5 years except for adequately managed basal cell carcinoma and squamous cell carcinoma of the skin

3. Presence or history of severe allergy or anaphylactic reactions, such as hypersensitivity to salicylates

4. Present pregnancy or breastfeeding

5. History within the last two years or current abuse of drugs or alcohol (>40 g of alcohol/day equivalent to >1 L of beer/day, 0.5 L of wine/day, or 6 glasses (2 cL) of liquor/day)

6. History of previous gastrointestinal surgery

7. Present diseases of the ano-rectum, including fistulas, anatomic irregularities, rectal tone compromising the ability to hold the enema

8. Intake of prescribed medication, over-the-counter (OTC) medication, or herbal medicines, within 2 weeks or 5 half-lives of the drug, whichever is longer, prior to 1st dose of mesalazine. Topical treatments of bacterial or fungal infection are allowed if stopped before first dose of IMP

9. Participate in any clinical trial within the last 12 weeks preceding Screening or longer if judged by the investigator to possibly influence the outcome of the current study

10. High daily consumption of caffeine-containing beverages (e.g. more than five cups of coffee or equivalent) within the last 12 weeks preceding Screening with a risk of withdrawal symptoms arising during the study that may confound the safety evaluation

11. Blood donation or major blood loss (≥500 mL) within the last 12 weeks preceding the first day of dosing

12. Previously recruited in this study and has been treated with at least one IMP

13. Mental incapacity or language barrier precluding adequate understanding or co-operation

14. Female subjects whose menstruation likely overlaps the planned Treatment Period (Day 1 to 8)

15. Considered by the investigator to be unsuitable to participate in the study for any other reason

4.2 Method of Assigning Subjects to Treatment Groups

4.2.1 Recruitment

Screened subjects will receive a unique screening number independent of the subject number. The screening number is a unique number, which is allocated to a subject when he/she first attends the trial site for the Screening. Eligible subjects who are assigned to IMP administration will have subject numbers in the sequence: 01, 02, 03 … assigned sequentially.

4.3 Restrictions

4.3.1 Prior and Concomitant Therapies

A prior medication is defined as any medication which was used at any time from signing ICD to 1st dose of IMP. A concomitant medication is defined as any medication that was administered at any time after the first dose of IMP up to completion of the End-of-trial examination.

No concomitant medication is allowed, except necessary treatment for AEs. Any prior or concomitant medication taken during the study will be recorded in the Case Report Form (CRF), including dose and dosage regimen together with the main reason for its prescription.

4.3.2 Prohibited Therapy

No therapy or medication will be allowed, except what is mentioned in Section 4.3.1. If a subject uses concomitant medication that is prohibited per protocol (e.g. any medication other than those given to treat an AE), the Investigator will decide (if possible after agreement with the Sponsor) if the subject can be permitted to continue the trial.

The following drugs as concomitant therapy for the treatment of AEs are prohibited, for the reason of potential drug interactions:

- Corticoids: the concomitant administration of corticoids may increase the danger of bleedings in the gastro-intestinal tract.
- Anticoagulants: in case of concomitant administration of anticoagulants, the haemorrhagic tendency may increase.
- Sulphonyl urea-like oral antidiabetic drugs: The hypoglycaemic action may be increased.
- Spironolactone, furosemide: the diuretic action may be decreased.
- Probenecide, sulfinpyrazone: the uricosuric effect may be decreased.
- Antimetabolites (e.g. Methotrexat, Mercaptopurin, Azathioprin): their toxicity may possibly increase.

- Rifampicin: the antituberculous action may be decreased.

ATC codes will be used to identify these prohibited therapies.

4.3.3 Other Restrictions

To avoid a positive result on the alcohol breath test used in this study, subjects must agree to abstain from drinking alcoholic beverages between signing ICD and End-of-trial examination.

Subjects must abstain from drinking large volumes of caffeine containing beverages (> 5 cups of coffee or equivalent) between signing ICD and End-of-trial examination.

Subjects are not eligible for this trial if they participated in clinical trials within 12 weeks of Screening, or longer if the Investigator feels the compound could influence this trial.

Subjects are not eligible for this trial if they took salicylates within 2 weeks prior to 1st dose of IMP, or longer if the Investigator feels the compound(s) could influence this trial (e.g. interfere the bioanalysis of mesalazine in plasma).

Subjects should abstain from strenuous physical activity that is not within their normal weekly routine beginning 48 hours before 1st dose of IMP until completion of the End-of-trial visit.

Only current non-smokers are eligible for the trial. Subjects are not allowed to smoke for the duration of their participation in the study.

Female subjects whose menstruation is likely to overlap Treatment Period (Day 1 to 8) are not eligible for the trial.

Subjects are not eligible if they are hypersensitivity to salicylates, such as acetylsalicylic acid and sodium salicylate.

4.4 Withdrawal Criteria

Subjects have the right to withdraw from the trial at any time for any reason without the need to justify their decision. However, where disclosed, the Investigator should record the main reason for the subject's withdrawal in the subject's CRF. The Investigator also has the right to withdraw subjects due to safety concerns or if they are non-compliant with study procedures to an extent judged likely to affect the validity of the study.

Subjects who flow out more than 20 g of enema after 1st or 7th dose, or flow out more than 50 g of enema after one of 2nd to 6th doses, are considered non-compliance of enema administration and maintaining. Adult diaper will be used to evaluate IMP compliance. If diaper weight increases more than 20 g after 1st or 7th dose, or if diaper weight increases more than 50 g after one of 2nd to 6th dose, the corresponding subject will be withdrawn from the trial.

In the case that female subjects starting menses during treatment period (Day 1 to 8), she should be withdrawn from the trial in the following situation:

- Menstruation starts before 7th dose of IMP administration (i.e. 9-10 p.m. on Day 7).
- Menstruation starts after 7th dose of IMP administration and the 7th diaper weight increases more than 20 g.

Any subject found to be pregnant at any time during the trial should be withdrawn immediately. The sponsor must be informed immediately of any pregnancy (see Section 8.3).

For any discontinuation or withdrawal, the Investigator must obtain all protocol-required details including the date of the premature termination which will be documented along with the main reason for withdrawal in the subject's CRF.

Subjects who discontinue or are withdrawn from study should be assessed for unreported AEs or concomitant medication use beforehand, if possible. In addition, they should have complete follow-assessments including a physical examination (excluding height), vital signs, 12-lead ECG, serum pregnancy test (females), clinical chemistry, haematology, and urinalysis at End-of-trial examination.

If the reason for discontinuation or withdrawal is an AE, the specific event or the main laboratory abnormality (-ies) will be recorded in the CRF. All subjects who discontinue or are withdrawn because of AEs will be followed up at suitable intervals in order to evaluate the course of the AE and to ensure reversibility or stabilisation. The outcome of the event will be recorded. Refer to Section 8.5 for further information on follow-up of AEs.

5 TREATMENTS

5.1 Treatments Administered

5.1.1 Investigational Medicinal Product (IMP)

The IMP is a rectal suspension of mesalazine, 1 g in 100 mL aqueous acetate buffer with a pH value between 4.4 and 5.0. The use of a suspension instead of a solution is considered advantageous as more drug substance will be in contact with the inflamed mucosa for a longer time. This is important as the therapeutic action of mesalazine depends on the contact of the drug with the disease areas of the intestinal mucosa.

Subjects will receive 1 g/100 mL mesalazine enema once daily at bedtime for 7 days. IMP will be administered by authorised staff at the trial site to ensure IMP compliance. The enema will be pre-heated to 30~37°C, inside its aluminium foil pack. The first dose of IMP should be administered at least 22 h post placebo enema administration.

Instruction of IMP administrion: Immediately before use take the enema bottle out of the aluminium foil pack and shake it well. To break the seal twist the nozzle clockwise one full turn (the nozzle should then be in the same direction as before turning). Authorised staff puts his/her hand in one of the plastic disposal bags provided in the pack. To administer the enema, the subjects should lie on his/her left side with the left leg straight and the right leg bent forward for balance. Carefully insert the applicator tip into the rectum. Maintain sufficient steady hand pressure while dispersing the bottle content. The bottle content should be applied within max. 30-40 seconds. Once the bottle is empty, withdraw the tip with the bottle still compressed. The enema should be retained in the bowel. Remain relaxed in the administration position for 5-10 minutes or until the urge to pass the enema has disappeared. Roll the plastic disposal bag over the empty bottle. Discard it and wash your hands. More IMP administration details refer to Introduction of IMP Administration (See 17Appendix 1).

5.1.2 Non-Investigational Medicinal Product (NIMP)

Placebo enema (100 mL) is identical to IMP in appearance, but does not contain mesalazine. The placebo enema is used to test the ability of enema retainment of subjects at Screening. Subject will receive two doses of placebo enema. The first dose will be administered during Day -14 to -2 and the enema will be required to be retained for 30 minutes. The second dose will be administered on Day -1 and the enema will be required to be retained for 8 h. The placebo enema is administered as the same way as IMP administration, including pre-heating.

5.2 Characteristics and Source of Supply

Although IMP is not approved in China, it is commercially available in other 71 countries. IMP is provided by Ferring Pharmaceuticals A/S. IMP is manufactured and handled according to the

principles of Good Manufacturing Practice (GMP). No modification will be made from its usual commercial state, other than application of trial specific labelling.

Placebo enema is provided by Ferring Pharmaceuticals A/S. It is manufactured and handled according to the principles of Good Manufacturing Practice (GMP).

5.2.1 Investigational Medicinal Product

Brand name:	PENTASA®.
Active substance:	Mesalazine (5-Aminosalicylic acid, 5-ASA).
Formulation:	Rectal suspension.
Strength:	1 g/100 mL.
Source of supply:	Ferring Pharmaceuticals A/S, Copenhagen, Denmark

5.2.2 Non-Investigational Medicinal Product

Brand name:	Not applicable.
Active substance:	None.
Formulation:	Rectal suspension.
Strength:	100 mL.
Source of supply:	Ferring Pharmaceuticals A/S, Copenhagen, Denmark

5.3 Packaging and Labelling

5.3.1 Investigational Medicinal Product

Packaging and labelling of IMP will be performed under the responsibility of the IMP department at Ferring Pharmaceuticals A/S in accordance with GMP and national regulatory requirements.

IMP will be supplied kits labelled with trial specific labels. The labels will also contain a self-adhesive tear-off part to be affixed to the drug accountability log.

5.3.2 Non-Investigational Medicinal Product

Packaging and labelling of NIMP will be performed under the responsibility of the IMP department at Ferring Pharmaceuticals A/S in accordance with GMP and national regulatory requirements.

NIMP will be supplied kits labelled with trial specific labels. The labels will also contain a self-adhesive tear-off part to be affixed to the drug accountability log.

5.4 Conditions for Storage and Use

The investigator will ensure that the IMP and NIMP will be stored in appropriate conditions in a secure location with controlled access. The storage compartments must be monitored regularly with Temperature Monitors and the values shall be documented. Any deviation from stated storage temperature must be reported to the Sponsor without delay and the investigational product must not be used until further instructions from the sponsor are received.

5.4.1 Investigational Medicinal Product

The IMP will only be administered to subjects who meet the inclusion/exclusion criteria. IMP should be stored below 25°C and not be refrigerated or freezed. IMP should be stored in the original package, as the product is sensitive to light.

5.4.2 Non-Investigational Medicinal Product

NIMP should be stored below 25°C and not be refrigerated or freezed. NIMP should be stored in the original package.

5.5 Blinding / Unblinding

As this trial is open-labelled and has no control group, no blinding will take place.

5.6 Treatment Compliance

5.6.1 Dispensing and Accountability

Dispensing of IMP and NIMP will be performed by authorised trial personnel (e.g., trial nurse or pharmacist). The Investigator (or his/her designated personnel, e.g., trial nurse) will maintain a Drug Dispensing Log detailing the dates and quantities of IMP and NIMP dispensed to, and used by, each subject, as well as the batch numbers. The trial monitor will verify the drug accountability during the trial.

5.6.2 Assessment of Enema Retention

IMP retainment compliance is defined as ⩾80% enema retained after 8 h post 1st and 7th doses, and ⩾50% enema retained after 8 h post 2nd to 6th doses. The compliance of treatment will be evaluated using the weight gain of a diaper. Before each administration, a new (unused) adult diaper weight will be measured and recorded, and then the diaper will be applied. The diaper weight will be measured and recorded again 8 h post-dose. The diaper should be weighted no more than 10 minutes following being taken off. If diaper weight increases more than 20 g after 1st or 7th dose, or if diaper weight increases more than 50 g after one of 2nd to 6th dose, the corresponding subject is considered not compliant with IMP administration and will be withdrawn from the trial.

5.7 Return and Destruction of Medicinal Products

All unused IMP and NIMP will be destroyed/returned for destruction, as instructed by Ferring IMP Department and in accordance with local requirements, after the drug accountability has been finalised, verified by the monitor, and signed off by the Investigator.

6 TRIAL PROCEDURES

6.1 Trial Flow Chart

Refer to Table 1 for the trial flow chart and Figure 1.

Assessment	Screening		Treatment Period								End-of-trial examination
**Day**	**-14 to -2**	**-1**	**1**	**2**	**3**	**4**	**5**	**6**	**7**	**8**	**9**
Informed consent	X										
Inclusion/exclusion criteria	X	X	X								
Demographics	X										
Medical history	X										
Physical examination	X										X [a]
Digital rectal examination	X										
Stool test	X										
Vital signs [b]	X	X	X	X	X	X	X	X	X	X	X
12-lead ECG	X										X
Serum pregnancy test (females) [c]	X	X									X
Urine drug screen	X	X									
Alcohol breath test	X	X									
Serology/virology [c]	X										
Clinical chemistry [c]	X	X [d]		X [e]							X
Haematology [c]	X	X [d]		X [e]							X
Urinalysis [c]	X	X [d]		X [e]							X
Enema retainment test (30 min) [f]	X										
Menstrual history check (females) [g]	X	X									
Enema retainment test (8 h) [h]		X									
Admission to trial site		X									
Standardised meals		X	X	X	X	X	X	X	X	X	X
Fasting and water abstain [i]		X	X	X	X	X	X	X	X		
Evacuation of bowel [j]		X	X	X	X	X	X	X	X		
Evacuation of bladder [k]		X	X	X	X	X	X	X	X		
Adult diaper application and weight measurement [l]		X	X	X	X	X	X	X	X		
(N)IMP weighting [m]		X	X	X	X	X	X	X	X		
Administration of IMP [n]			X	X	X	X	X	X	X		
PK blood sampling [o]			X	X			X	X	X	X	
Urine sampling [p]			X						X	X	
IMP retainment [q]			X	X	X	X	X	X	X		
Bedpan application [r]		X	X	X	X	X	X	X	X		
Defecation and time record [s]			X	X	X	X	X	X	X	X	
Adverse events	X [t]	X	X	X	X	X	X	X	X	X	X
Prior/concomitant medication	X	X	X	X	X	X	X	X	X	X	X
Discharge from trial site [u]											X

[a] Body height will not be measured on End-of-trial examination.

[b] Vital signs (blood pressure, pulse rate, axillary temperature and respiratory rate) will be measured after 5 minutes resting during Day -14 to -2, on Day -1, on each day of Treatment Period (Day 1 to 8), and as part of the End-of-trial examination, between 8-10 a.m. on each day. Blood pressure will be measured in a supine position.

[c] Analyses performed in local laboratory.

[d] The tests will not be performed on Day -1 if they were performed within 7 days before Day -1 (i.e. between Day -8 to Day -2).

[e] Blood and urine samples will be taken before 2nd dose.

[f] Enema retainment test (30 min, performed during Day -14 to -2) must be done prior to DRE. A placebo enema (100 mL) will be administered to subjects. The placebo enema is pre-heated to 30~37°C, and dosed in the same way as IMP administration. A diaper, which has been weighted, will be applied to a subject. The diaper will be weighted again after 30 minutes post placebo enema administration. The diaper should be weighted no more than 10 minutes following being taken off. Eligible subjects should retain the enema for at least 30 minutes with no more than 20 g diaper weight increase.

[g] Menstrual history (females) will be checked during Day -14 to -2 and on Day -1. The qualified female subjects' menstruation will not likely overlap Treatment Period.

[h] An enema retainment test (8 h) will be performed on Day -1. A placebo enema (100 mL) will be administered to subjects at least 22 h prior to 1st dose of IMP administration. All the procedures associated with IMP compliance improvement and evaluation, including fasting and water abstain [i], evacuation of bowel [j], evacuation of bladder [k], adult diaper application and weight measurement [l], (N)IMP weighting [m], (N)IMP retainment [p], bedpan application [q], and defecation and time record [r], are the same as IMP administration. Eligible subjects should retain the enema for at least 8 hours with less than or equivalent to 20 g diaper weight increase.

[i] Subjects will be fasted and abstained to water 2 h pre-dose until at least 8 h post-dose.

[j] Water enema will be used to subjects approximately 2 h before NIMP administration on Day -1, 1st and 7th IMP doses to facilitate bowel evacuation. Approximately 2 h before 2nd to 6th IMP administrations, subjects will be required to go to toilet and empty their bowls as clean as possible, but water enema will not be used.

[k] A visit to toilet to evacuate bladder is required approximately 10 min before NIMP administration (Day -1) and before each IMP administration (Day 1 to 7).

[l] Adult diaper will be used to evaluate the compliance of (N)IMP retainment. New (unused) diaper weight will be measured and recorded, and afterwards the diaper will be applied to a subject. After 8 h post-dose, the diaper will be weighed again. The diaper should be weighted no more than 10 minutes following being taken off.

[m] After (N)IMP is pre-heated to 30~37°C inside its foil pack, the (N)IMP will be wiped dry and weighted without foil pack. After (N)IMP administration, the weight of used (N)IMP will be weighted again. The weight of unused and used (N)IMP will be recorded.

[n] IMP is administered approximately 9-10 p.m. on Day 1-7.

[o] Blood samples for analysis of mesalazine will be collected repeatedly up to 24 hours following 1st, 5th and 7th doses. The sampling times associated with 1st, 5th and 7th doses are: immediately pre-dose and 0.5, 1, 2, 3, 4, 5, 6, 7, 8, 10, 12, 14, 16, 20 and 24 h post-dose (before the next dose).

[p] For drug excretion study, urine will be sampled after the 7th dose, within the intervals of 0-12 h and 12-24 h post-7th dose. Blank urine will also be sampled prior to 1st dose.

[q] IMP should be retained at least 8 h post-dose.

[r] Subjects who urinate within 8 h post-dose will use bedpans in bed.

[s] After diaper weight is measured, subjects will be required to visit to toilet during 8-10 h post-dose and the defecation time (as time interval) will be recorded.

[t] Start to collect AE since the first visit.

[u] Subjects will only be discharged from the trial site if results of End-of-trial examination are considered 'normal' or 'abnormal, not clinically significant'. In case any indicator is considered 'abnormal, clinically significant' at this time, additional managements will be taken at the discretion of the Investigator before discharging of the subjects.

Figure 1 Trial Scheme

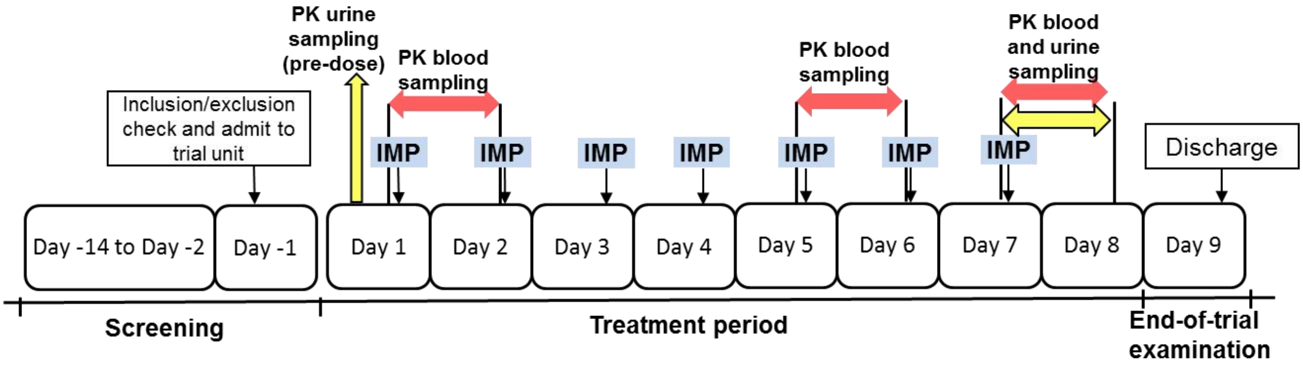

Note: There is no Day 0 in the study.

6.2 Screening

Day -14 to -2

All subjects will provide informed consents and undergo an entry examination to evaluate their health status. This examination will be conducted between Day -14 and Day -2 before the planned administration of the first dose of IMP on Day 1.

This screening examination will consist of the following:

- Medical history as specified in Section 7.2.7;
- Physical examination as specified in Section 7.1.8;
- Collection of demographic information Section 7.2.6;
- Vital signs as specified in Section 7.1.2;
- 12-lead ECG as specified in Section 7.1.3;
- Clinical Chemistry as specified in Section 7.1.5;
- Haematology as specified in Section 7.1.6;
- Urinalysis as specified in Section 7.1.7;
- Urine drug screen and alcohol breath test as specified in Section 7.2.5;
- Serology and virology as specified in Section 7.2.3;
- Digital rectal examination as specified in Section 7.2.1;
- Stool test as specified in Section 7.2.2;
- Prior medication as specified in Section 7.2.8;
- Serum pregnancy test (females) as specified in Section 7.2.4;
- Enema retainment test (30 min) as specified in Section 7.2.9;
- Menstrual history (females) of past 6 months, including mean length of menstrual cycle, mean length of menstruation and last start date of menses.

Day -1

Subjects will be admitted to the trial site on Day -1. Subjects will undergo a brief check of inclusion/exclusion criteria and an inclusion/exclusion criteria check on Day -1 including the following:

- Vital signs as specified in Section 7.1.2;
- Serum pregnancy test (females) as specified in Section 7.2.4;
- Urine drug screen and alcohol breath test as specified in Section 7.2.5;
- Clinical Chemistry as specified in Section 7.1.5;
- Haematology as specified in Section 7.1.6;
- Urinalysis as specified in Section 7.1.7;

- Prior medication as specified in Section 7.2.8;
- Enema retainment test (8 h) as specified in Section 7.2.10;
- Assessment of pre-treatment AEs as specified in Section 7.1.4;
- Last end-of-menstruation day of female subjects will be checked and the day should be within 7 days prior to Day 1 (Day -7 to Day -1).

Clinical chemistry, haematology and urinalysis will not be performed if they were performed within 7 days before Day -1 (i.e. between Day -8 to Day -2).

Eligible subjects will be considered as successfully screened and admitted to the IMP dosing.

6.3 Treatment Period

The duration of Treatment Period is from Day 1 to 8.

Prior medication and pre-treatment AEs will be assessed on Day 1 before 1st dose of IMP administration.

Figure 2 Procedures associated with IMP compliance improvement and assessment

The following procedures will be conducted before or after each IMP administration.

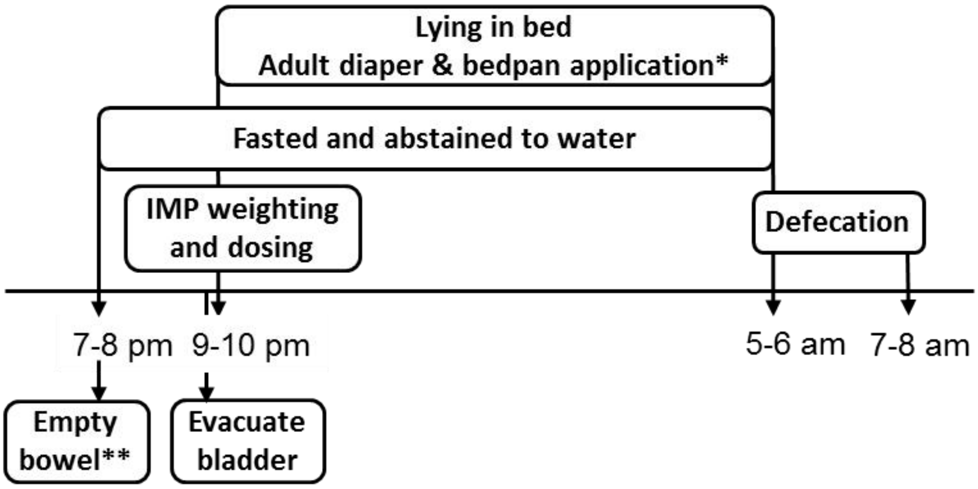

* Bedpan will be applied if needed.
** Water enema will be used before 1st and 7th dose to facilitate bowel empty.

Subjects will receive a daily rectal dose of PENTASA® enema (1 g/100 mL) 9-10 p.m. on Day 1-7 and be required to retain the enema for at least 8 h after IMP dosing. The following procedures (Figure 2) will be conducted before or after each IMP administration to improve and evaluate the compliance of IMP retainment (unless special instructions):

- Subjects will be fasted and abstained to water 2 h pre-dose (i.e. 7-8 p.m.) until 8 h post-dose (i.e. 5-6 a.m. in the morning of next day).

- Approximately 2 h before each dose, subjects will be required to go to toilet and empty their bowels as clean as possible. Water enema will be used to subjects to facilitate bowel evacuation before 1st and 7th doses, while water enema will not be used before 2nd-6th doses.
- A visit to toilet to evacuate bladder is required approximately 10 min before each dose.
- After IMP is pre-heated to 30~37°C inside its foil pack, the IMP will be wiped dry and weighted without foil pack. After IMP administration, the weight of used IMP will be weighted again. The weight of unused and used IMP will be recorded.
- Adult diaper will be used to evaluate the compliance of IMP retainment. The weight of a new (unused) diaper will be measured and recorded, and afterwards the diaper will be applied to a subject. After 8 h post-dose, the diaper will be weighed again. The diaper should be weighted no more than 10 minutes following being taken off. If diaper weight increases more than 20 g after 1st or 7th dose, or if diaper weight increases more than 50 g after one of 2nd to 6th dose, the corresponding subject will be withdrawn from the trial.
- Subjects who urinate within 8 h post-dose will use bedpans in bed.
- After diaper weight is measured, subjects will be required to visit to toilet during 8-10 h post-dose and the defecation time (as time interval) will be recorded.

Blood samples for analysis of mesalazine will be collected repeatedly up to 24 h following 1st, 5th and 7th doses. The sampling times associated with each of the three doses are: immediately prior to dosing and 0.5, 1, 2, 3, 4, 5, 6, 7, 8, 10, 12, 14, 16, 20 and 24 h post-dose (before the next dose). One additional blood sample will be taken immediately prior to the 6th dose. Approximately 4 mL blood will be sampled at each time point.

Urine samples will be collected for the determination of mesalazine and its main metabolite, N-acetyl-mesalazine. 5~10 mL blank urine will be collected prior to the 1st dose and all urine during intervals of 0-12 h and 12-24 h following the 7th doses will be collected.

Vital signs as described in Section 7.1.2 will be performed on each day from Day 1 to Day 8.

Besides vital signs, the following tests will be performed on Day 2:
- Clinical Chemistry as specified in Section 7.1.5;
- Haematology as specified in Section 7.1.6;
- Urinalysis as specified in Section 7.1.7.

Subjects will be interviewed by trained staff at the trial site to elicit information on possible AEs and concomitant medication use. If AEs are reported, the investigator may decide to perform/repeat a clinical examination and/or laboratory tests.

6.4 End-of-trial Examination

The End-of-trial examination will be performed on Day 9. This examination will consist of:

- Physical examination (excluding height), as specified in Section 7.1.8;
- Vital signs as specified in Section 7.1.2;
- 12-lead ECG as specified in Section 7.1.3;
- Clinical Chemistry as specified in Section 7.1.5;
- Haematology as specified in Section 7.1.6;
- Urinalysis as specified in Section 7.1.7;
- Serum pregnancy test (females) as specified in Section 7.2.4;
- Assessments of AEs and concomitant medication.

Subjects will only be discharged from the trial site if results of End-of-trial examination are considered ‘normal’ or ‘abnormal, not clinically significant’. In case any indicator is considered ‘abnormal, clinically significant’ at this time, additional managements will be taken at the discretion of the Investigator before discharging of the subjects.

On-going AEs will be followed-up according to the procedures specified in Section 8. Clinically significant laboratory values will be followed up until they return to within the reference range or until the medical condition of the subject is stable.

7 TRIAL ASSESSMENTS

The majority of assessments in this trial are based on laboratory tests. A Laboratory Manual will describe the procedures associated with the quantitation of mesalazine in plasma including sampling and shipment procedures details, contact information, storage conditions, etc. The manual will be provided to the trial site before the start of the trial. All other laboratory procedures will be performed in the local laboratory according to local practice.

Time points for PK blood sampling and other trial assessments are provided in Table 1 Trial Flow Chart.

7.1 Assessments Related to Endpoints

7.1.1 Pharmacokinetic Assessments

The primary PK endpoints are based on the determination of plasma mesalazine concentration. Bioanalysis of plasma concentrations of mesalazine will be performed by a qualified bioanalytical laboratory using a validated LC-MS/MS method.

Blood samples for analysis of mesalazine will be collected repeatedly up to 24 h following 1st, 5th and 7th doses. The sampling times associated with each of the three doses are: immediately prior to dosing and 0.5, 1, 2, 3, 4, 5, 6, 7, 8, 10, 12, 14, 16, 20 and 24 h post-dose (before the next dose). One additional blood sample will be taken immediately prior to the 6th dose. Approximately 4 mL blood will be sampled at each time point.

One of the secondary PK endpoints, to determine the total cumulative amount of mesalazine and N-acetyl mesalazine excreted in urine, is based on the determination of urine mesalazine and N-acetyl mesalazine concentration and urine volume. Total urine volume during each interval of 0-12 h and 12-24 h following the 7th dose will be measured by appropriate measuring cylinders at the trial site. Simultaneous determination of urine concentrations of mesalazine and N-acetyl mesalazine will be performed by a qualified bioanalytical laboratory using a validated LC-MS/MS method.

Urine samples for analysis of mesalazine and its main metabolite, N-acetyl mesalazine will be collected prior to 1st dose and during intervals of 0-12 h and 12-24 h following the 7th doses.

The actual sampling times will be recorded in CRF and used for PK parameter calculation.

7.1.2 Vital Signs

Vital signs comprise supine blood pressure, pulse, axillary temperature and respiratory rate. Vital signs will be measured during Day -14 to Day-2, on Day -1, on each day of Treatment Period (Day 1 to 8), and on Day 9 End-of-trial examination.

Systolic and diastolic blood pressure will be measured on each occasion using the same arm and after the subject has been in the supine position for at least 5 minutes. All recordings will be performed using standard equipment; either automatic or manual measurements are acceptable, though the Investigator should continue to use the same method for all assessments of an individual subject as was used for the first measurement. Clinically significant abnormal values will be reported as AEs.

7.1.3 Electrocardiogram

A 12-lead ECG will be recorded at Screening (during Day -14 to -2) and on Day 9 End-of-trial examination.

ECG recordings will capture at least four QRS complexes, i.e. three evaluable RR intervals. The Investigator or designee will evaluate the clinical significance of the ECG as ‘normal’, ‘abnormal not clinically significant’, or ‘abnormal clinically significant’. Any occurrence of de- or re-polarisation disorders, arrhythmic disorders, or other abnormalities will be assessed and any changes compared with the pre-IMP record will be commented upon. Abnormal, clinically significant events will be reported as AEs.

7.1.4 Adverse Events

Information on AEs will be collected from the time a subject signs the ICD until completion of the End-of-trial examination. For each AE the following parameters will be recorded on the Adverse Event Log: description of the event, date of onset, intensity, causal relationship to IMP, action taken with the IMP, other actions taken, seriousness of the AE, date of outcome, and outcome.

For further information on definitions and reporting of AEs and SAEs, see Section 8.

7.1.5 Clinical Chemistry

Clinical chemistry parameters listed in Table 3 will be performed by the Local Laboratory during Day -14 to -2, on Day -1 and Day 2 of treatment period (Day 1 to 8) and on Day 9 End-of-trial examination. The tests will not be performed on Day -1 if they were performed within 7 days before Day -1 (i.e. between Day -8 to Day -2). All data will be entered onto the CRF by the Investigator.

Clinical chemistry performed during Screening and again on Day -1 (if applicable) will be used to determine whether the subject is eligible for participation in the trial. Day -1 clinical chemistry data will serve as baseline for the analyses of the safety study results; however, if the tests are waived on Day -1, the data created during Day -14 to Day -2 will be used as baseline. Subjects with clinically relevant abnormal laboratory values in the opinion of the Investigator at Screening or on Day -1 should not be enrolled to treatment on Day 1.

Further clinical chemistry will be performed on Day 2 and Day 9.

The Investigator will review all clinical chemistry results and evaluate whether they are low, normal, or high based on the Local Laboratory reference ranges. In addition, the Investigator will assess and document the clinical significance of each result as 'normal', 'abnormal not clinically significant' or 'abnormal and clinically significant'. Laboratory Reports will be signed and dated by the Investigator. 'Abnormal and clinically significant' changes in laboratory values should be reported as AEs (see Section 8).

Table 3 Safety laboratory parameters - Clinical chemistry

Albumin
Alkaline phosphatase
Alanine aminotransferase
Aspartate aminotransferase
Chloride
Calcium
(Total) Cholesterol
Creatinine
Creatinine clearance
Gamma-glutamyltransferase
Potassium
Sodium
Total bilirubin
BUN (blood urea nitrogen)
Uric acid

7.1.6 Haematology

Haematology parameters listed in Table 4 will be performed by the Local Laboratory at Screening (during Day -14 to -2), on Day -1 and Day 2 of treatment period (Day 1 to 8) and on Day 9 End-of-trial examination. The tests will not be performed on Day -1 if they were performed within 7 days

before Day -1 (i.e. between Day -8 to Day -2). All data will be entered onto the CRF by the Investigator.

Haematology performed during Screening and again on Day -1 (if applicable) will be used to determine whether the subject is eligible for participation in the trial. Day -1 haematology data will serve as baseline for the analyses of the safety study results; however, if the tests are waived on Day -1, the data created during Day -14 to Day -2 will be used as baseline. Subjects with clinically relevant abnormal laboratory values in the opinion of the Investigator at Screening (during Day -14 to -2) or on Day -1 should not be enrolled to treatment on Day 1.

Further haematology will be performed on Day 2 and Day 9.

The Investigator will review all haematology results and evaluate whether they are low, normal, or high based on the Local Laboratory reference ranges. In addition, the Investigator will assess and document the clinical significance of each result as 'normal', 'abnormal not clinically significant' or 'abnormal and clinically significant'. Laboratory Reports will be signed and dated by the Investigator. 'Abnormal and clinically significant' changes in laboratory values should be reported as AEs (see Section 8).

Table 4 Safety laboratory parameters - Haematology

Haematocrit
Haemoglobin
Mean cell haemoglobin concentration
Mean cell volume
Platelet count
Red blood cell count
White blood cell count:
Differential count:
Neutrophils
Eosinophils
Basophils
Lymphocytes
Monocytes

7.1.7 Urinalysis

Urinalysis parameters listed in Table 5 will be performed by the Local Laboratory during Day -14 to Day -2, on Day -1 and Day 2 of treatment period (Day 1 to 8) and on Day 9 End-of-trial examination. The tests will not be performed on Day -1 if they were performed within 7 days before Day -1 (i.e. between Day -8 to Day -2). The urinalysis assessments should be performed using a sample of mid-stream urine. All data will be entered onto the CRF by the Investigator.

Urinalysis performed during Day -14 to Day -2 and again on Day -1 (if applicable) will be used to determine whether the subject is eligible for participation in the trial. Day -1 urinalysis data will serve as baseline for the analyses of the safety study results; however, if the tests are waived on Day -1, the data created during Screening will be used as baseline. Subjects with clinically relevant abnormal laboratory values in the opinion of the Investigator during Day -14 to Day -2 or on Day -1 should not be enrolled to treatment at bedtime on Day 1.

Further urinalysis will be performed on Day 2 and Day 9.

The Investigator will review all urinalysis results and evaluate whether they are low, normal, or high based on the Local Laboratory reference ranges. In addition, the Investigator will assess and document the clinical significance of each result as ‘normal’, ‘abnormal not clinically significant’ or ‘abnormal and clinically significant’. Laboratory Reports will be signed and dated by the Investigator. ‘Abnormal and clinically significant’ changes in laboratory values should be reported as AEs (see Section 8).

Table 5 Safety laboratory parameters - Urinalysis

Blood
Glucose
Ketones
Leucocytes
pH
Protein

7.1.8 Physical Examination

A full physical examination will be performed during Day -14 to Day -2 to ensure eligibility according to the inclusion and exclusion criteria. Height and body weight will be measured for BMI calculation during Day -14 to Day -2. A physical examination will also be performed before discharge from the trial site at the End-of-trial Exam; however, height will only be measured at Screening visit.

The physical examination will include: general appearance, central and peripheral nervous system, head and neck (including ears, eyes, nose, mouth and throat), respiratory system, cardiovascular system, gastrointestinal system, lymphatic system, urinary system, reproductive system including breasts, musculoskeletal system and skin. All abnormal findings will be reported as AEs.

7.2 Other Assessments

7.2.1 Digital Rectal Examination

Digital rectal examination (DRE) is a physical examination to test abnormalities in the lower rectum, and nearby organs or tissues. A DRE will be performed during Screening (during Day -14 to -2) to ensure healthy rectal and anal status of enrolled subjects. The enrolled subjects should have healthy rectal and anal status based on DRE. Subjects with other findings according to DRE, such

as abnormalities in prostate gland, could be enrolled at the investigator's discretion according to Inclusion/Exclusion criteria in Section 4.1.

7.2.2 Stool test

A stool test will be performed at Screening (during Day -14 to -2) only. Subjects positive for any of the following are not eligible for the trial:

- Occult blood;
- Blood cells;
- Mucus;
- Eggs and segments of parasites.

7.2.3 Serology and Virology

Serology and virology assessments (measured in serum) will be performed at Screening only. Subjects positive for any of the following are not eligible for the trial:

- HIV-AB;
- HBs-AG;
- HCV-AB;
- TP-AB.

7.2.4 Serum Pregnancy Test (females)

Serum beta-human chorionic gonadotrophin (βhCG) will be determined during Day -14 to Day -2, on Day -1 and Day 9 using validated standard methods. The results of all serum pregnancy tests (females) will be entered into the CRF by the Investigator.

Female subjects with a positive pregnancy test/who become pregnant at any time during the study are ineligible to continue participation and should be immediately withdrawn (see Section 4.4) with the pregnancy reported to the Sponsor as specified in Section 8.3.

7.2.5 Urine Drug Screen and Alcohol Breath Test

Illicit drug use is not permitted at any time during the study. Alcohol is not permitted from signing ICD to End-of-trial examination (see Section 4.3.3).

Drug and alcohol screen assessments will be performed on Day -1. Subjects positive for ethanol (measured with Alkomat, breath test) or any of the following drugs are not eligible for IMP administration:

- Amphetamines
- Barbiturates
- Benzodiazepines
- Cannabinoids
- Tricyclic
- Methylenedioxymethamphetamine

(Marijuana)	Antidepressants (Nortriptyline)	(Ecstasy)
• Methadone	• Cocaine	• Morphine/Opiates
• Phencyclidine		

The drug-screening should be performed using fresh mid-stream urine.

7.2.6 Demographic and Baseline Data

Demographic and baseline data will be collected during the Screening including gender and date of birth. These data will be entered into the CRF.

7.2.7 Medical history

Information on clinically significant previous and concomitant illness (or signs and symptoms if a diagnosis has not been made) present before signing the ICD will be recorded in the CRF as medical history at the Screening. Subjects with certain past or current illnesses may not be eligible for the study (refer to the inclusion/exclusion criteria in Section 4.1).

7.2.8 Prior and Concomitant Medication

Prior and concomitant medications are defined in Section 4.3.1.

All prior medication and concomitant medication taken during the course of the study, from the initial Screening until the Day 9 End-of-trial examination, will be recorded in CRF by the Investigator using the brand name when available (otherwise the generic name). Other information that will be recorded includes the main reason for taking the medication, route of administration, and duration of treatment. If the concomitant medicine is a traditional Chinese medicine, it should be recorded as “traditional Chinese medicine”, along with the brand name and the essential components, if known.

7.2.9 Enema Retainment Test (30 min)

Enema retainment test (30 min) will be performed during Day -14 to -2 at Screening. It must be done prior to DRE, for the reason that DRE may impact on the function of sphincter ani, which may in turn impact on enema retainment test.

A placebo enema (100 mL) will be administered to subjects. The placebo enema is pre-heated to 30~37°C, and dosed in a same way as IMP administration (Appendix 1). The subjects will be required to retain the enema for at least 30 minutes.

A diaper will be used for the evaluation of the placebo enema retainment compliance. The weight of a new (unused) diaper will be measured and recorded, and afterwards the diaper will be applied to a subject. After 30 minutes post placebo enema dose, the diaper will be weighed again. The

diaper should be weighted no more than 10 minutes following being taken off. Subjects whose diaper weight increases more than 20 g will not be eligible for enrolment.

7.2.10 Enema Retainment Test (8 h)

Enema retainment test (8 h) will be performed on Day -1 using placebo enema (100 mL). The placebo enema must be dosed at least 22 h prior to 1st dose of IMP administration. All the procedure used for IMP compliance improvement and evaluation will be used in this test, including:

- Subjects will be fasted and abstained to water 2 h pre-dose until 8 h post-dose.
- Approximately 2 h before dose, water enema will be used for bowel cleaning.
- A visit to toilet to evacuate bladder is required approximately 10 min before dose.
- After NIMP is pre-heated to 30~37°C inside its foil pack, the NIMP will be wiped dry and weighted without foil pack. After NIMP administration, the weight of used NIMP will be weighted again. The weight of unused and used NIMP will be recorded.
- Adult diaper will be used to evaluate the compliance of NIMP retainment. The weight of a new (unused) diaper will be measured and recorded, and afterwards the diaper will be applied to a subject. After 8 h post-dose, the diaper will be weighed again. The diaper should be weighted no more than 10 minutes following being taken off.
- Subjects who urinate within 8 h post-dose will use bedpans in bed.
- After diaper weight is measured, subjects will be required to visit to toilet during 8-10 h post-dose and the defecation time (as time interval) will be recorded.

Subjects whose diaper weight increases more than 20 g will not be admitted to IMP administration.

7.3 Drug Concentration Measurements

The determinations of mesalazine in human plasma and mesalazine and N-acetyl-mesalazine in human urine will be outsourced to a qualified bioanalytical laboratory in China. The determinations will be based on LC-MS/MS methods and stable isotope-labelled internal standard is preferable. The method validation and sample analysis will be implemented according to relevant guidance [16].

7.4 Handling of Biological Samples

Plasma and urine samples for bioanalysis:

Each sample will be labelled to indicate the trial number, subject number, sampling date, and sampling time relative to dosing. Handling of samples including shipping information will be detailed in the Laboratory Manual. Bioanalysis samples will be stored for 2 years after finalization of Clinical Trial Report. In case a subject withdraws consent requesting his/her destruction of biological samples, the samples will be destructed immediately. The destruction of samples must be approved by Sponsor and documented. The confirmation of destruction must be sent to Sponsor.

Other biological samples:

Other than samples for bioanalysis of mesalazine (and N-acetyl-mesalazine), all other laboratory-based assessments will be performed in the local laboratories. Unused samples will be destroyed immediately after completion of testing according to local policy.

Irrespective of where they are analysed, no additional analyses beyond those specified in this protocol will be performed on the collected biological samples unless additional approval/consent has been gained from the Ethics Committee (EC), regulatory authorities (CFDA), and/or, to the extent possible, trial subjects beforehand. If, after reasonable efforts have been made, subjects still cannot be contacted to gain consent, but EC and/or CFDA approval has been given, then the additional analyses can be performed.

8 ADVERSE EVENTS

8.1 Adverse Event Definition

An adverse event is any untoward medical occurrence in a subject participating in a clinical trial. It includes:

- Any unfavourable and unintended sign, symptom or disease temporally associated with the use of the IMP, whether or not considered to be caused by the IMP.
- Adverse events commonly observed and adverse events anticipated based on the pharmacological effect of the IMP.
- Any laboratory abnormality, vital sign or finding from physical examination assessed as clinically significant by the investigator (note: findings from assessments and examinations done during screening are not adverse events, but are recorded as medical history).
- Accidental injuries, reasons for any change in medication (drug and/or dose), reasons for any medical, nursing or pharmacy consultation, or reasons for admission to hospital or surgical procedures.
- Overdoses and medication errors with and without clinical consequences.

Pre-treatment AE:

Any AE occurring after signing informed consent and before start of IMP, or a pre-existing medical condition that worsens in intensity after signed informed consent but before start of IMP.

Treatment Emergent AE:

Any AE occurring after start of IMP up to the date of completion of the Day 9 End-of-trial examination, or pre-treatment AE or pre-existing medical condition that worsens

8.2 Collection and Recording of Adverse Events

8.2.1 Collection of Adverse Events

The investigator must monitor the condition of the subject throughout the trial from the time of obtaining informed consent until the last visit.

The sources of adverse events cover:

- The subject's response to questions about his/her health (a standard non-leading question such as "How have you been feeling since your last visit?" is asked at each visit).
- Symptoms spontaneously reported by the subject.
- Investigations and examinations where the findings are assessed by the investigator to be clinically significant changes or abnormalities.

- Other information relating to the subject's health becoming known to the investigator (e.g. hospitalisation).

8.2.2 Recording of Adverse Events

The investigator must record all adverse events in the Adverse Event Log provided in each subject's CRF with information about:

- Adverse event
- Date and time of onset (time can be omitted, if applicable)
- Intensity
- Causal relationship to IMP
- Action taken to IMP
- Other action taken
- Date and time of outcome (time can be omitted, if applicable)
- Outcome
- Seriousness.

Each of the items in the Adverse Event Log is described in detail in the following sections.

Adverse Event

Adverse events should be recorded as diagnoses, if available. If not, separate signs and symptoms should be recorded. One diagnosis/symptom should be entered per record.

If a subject suffers from the same adverse event more than once and the subject recovers in between the events, the adverse events should be recorded separately. If an adverse event changes in intensity, a worst-case approach should be used when recording the event, i.e. the highest intensity and the longest duration of the event.[a]

Note the following: A procedure is not an adverse event; the reason for conducting the procedure is. Hospitalisation is not an adverse event; the reason for hospitalisation is. Death is not an adverse event, but the cause of death is (an exception is sudden death of unknown cause, which is an adverse event).

Date and Time of Onset

[a] Exception: if an adverse event with onset before the first IMP administration (i.e. a pre-treatment adverse event) changes in intensity, this must be recorded as two separate events. The initial adverse event should be recorded with outcome "not yet recovered" and the date and time of outcome is when the intensity changed. The second adverse event should be recorded with date and time of onset when the intensity changed.

The date of onset is the date when the first sign(s) or symptom(s) were first noted. If the adverse event is an abnormal clinically significant laboratory test or outcome of an examination, the onset date is the date the sample was taken or the examination was performed.

Intensity

The intensity of an adverse event must be classified using the following 3-point scale:

Mild: Awareness of signs or symptoms, but no disruption of usual activity.

Moderate: Event sufficient to affect usual activity (disturbing).

Severe: Inability to work or perform usual activities (unacceptable).

Causal Relationship to IMP

The possibility of whether the IMP caused the adverse event must be classified as one of the following:

Reasonable possibility:

There is evidence or argument to suggest a causal relationship between the IMP and the adverse event. The adverse event may occur as part of the pharmacological action of the IMP or may be unpredictable in its occurrence.

Examples:

- Adverse events that are uncommon but are known to be strongly associated with IMP exposure.
- Adverse events that are not commonly associated with IMP exposure, but the event occurs in association with other factors strongly suggesting causation, such as a strong temporal association or the event recurs on rechallenge.

No reasonable possibility:

There is no reasonable evidence or argument to suggest a causal relationship between the IMP and the adverse event.

Examples:

- Known consequences of the underlying disease or condition under investigation.
- Adverse events common in the trial population, which are also anticipated to occur with some frequency during the course of the trial, regardless of IMP exposure.

Action Taken to IMP

The action taken to the IMP in response to an adverse event must be classified as one of the following:

- No change (medication schedule maintained or no action taken)
- Withdrawn

Other Action Taken

Adverse events requiring therapy must be treated with recognised standards of medical care to protect the health and well-being of the subject. Appropriate resuscitation equipment and medicines must be available to ensure the best possible treatment of an emergency situation.

If medication is administered to treat the adverse event, this medication should be entered in the Concomitant Medication Log.

Date and Time of Outcome

The date and time the subject recovered or died.

Outcome

The outcome of an adverse event must be classified as one of the following:

- Recovered (fully recovered or the condition has returned to the level observed at initiation of trial treatment)
- Recovered with sequelae (resulted in persistent or significant disability/incapacity)
- Recovering
- Not yet recovered
- Fatal.

8.3 Pregnancy and Pregnancy Outcome

If a pregnancy occurs in female subjects, the IMP should be immediately stopped, the pregnant subjects should be withdrawn from the trial and Global Pharmacovigilance (GPV) at Ferring Pharmaceuticals must be informed using Pregnancy Report Form within three days. Note that pregnancy itself is not an SAE. The mother and the foetus must be followed-up at least until the birth of the infant and one month after the birth of the infant. In general, the follow-up will include the course; duration and the outcome of the pregnancy as well as neonatal health. If a pregnancy results in an abnormal outcome (such as birth defect, congenital anomaly, still birth, neonatal death etc.) this must be reported as a serious adverse event (SAE) to Ferring and China Food and Drug Administration (CFDA) according to the procedure described in Section 8.4.2. Any abnormal pregnancy outcome which the Investigator and/or Sponsor consider to be related to the IMP will be treated as an expedited report.

For male subjects, in cases in which a foetus may have been exposed through transmission of the IMP via semen following paternal exposure, and the pregnancy results in an abnormal outcome (such as birth defect, congenital anomaly, still birth, neonatal death etc.) this must be reported as a SAE to GPV at Ferring Pharmaceuticals and CFDA according to the procedure described in Section 8.4.2. Any abnormal outcome of the pregnancy which the Investigator and/or Sponsor consider to be related to the IMP will be treated as an expedited report.

8.4 Serious Adverse Events

8.4.1 Serious Adverse Event Definition

Serious Adverse Events during the Trial

An event is defined a serious adverse event if it:	Guidance
results in **death**	Any event resulting in a fatal outcome must be fully documented and reported, including deaths occurring within four weeks after the treatment ends and irrespective of the causal relationship to the IMP. The death of a subject enrolled in a trial is *per se* not an event, but an outcome.
is **life-threatening**	The term life-threatening refers to an AE in which the subject was at immediate risk of death at the time of the event. It does not refer to an event, which may have caused death if it were more severe.
requires in-patient **hospitalisation** or prolongation of existing hospitalisation	The term hospitalisation means that the subject was admitted to hospital or that existing hospitalisation was extended as a result of an event. Hospitalisation describes a period of at least 24 hours. Over-night stay for observation, stay at emergency room or treatment on an out-patient basis do not constitute a hospitalisation. However, medical judgement must always be exercised and when in doubt the case should be considered serious (i.e. if case fulfils the criterion for a medically important event). Hospitalisations for administrative or social purposes do not constitute an SAE. Hospital admissions and/or surgical operations planned before trial inclusion are not considered adverse events, if the illness or disease existed before the subject was enrolled in the trial, provided that the condition did not deteriorate during the trial.
results in persistent or significant **disability/incapacity**	Disability/incapacity means a substantial disruption of a person's ability to conduct normal life functions. In doubt, the decision should be left to medical judgement by the investigator.
is a **congenital anomaly/birth defect**	Congenital anomaly/birth defect observed in any offspring of the subject conceived during treatment with the IMP.
is an **important medical event**	Important medical events are events that may not be immediately life-threatening or result in death or hospitalisation but may jeopardise the subject or may require intervention to prevent one of the other outcomes listed in the definition above. Examples of important medical events include AEs that

An event is defined a serious adverse event if it:	Guidance
	suggest a significant hazard, contraindication or precaution, occurrence of malignancy or development of drug dependency or drug abuse. Medical and scientific judgement should be exercised in deciding whether events qualify as medically important.<br><br>Important medical events include any suspected transmission of an infectious agent via a medicinal product. Any organism virus or infectious particle (e.g. prion protein transmitting Transmissible Spongiform Encephalopathy), pathogenic or non-pathogenic, is considered an infectious agent. A transmission of an infectious agent may be suspected from clinical symptoms or laboratory findings indicating an infection in a subject exposed to a medicinal product.

Serious Adverse Events during Post-trial Follow-up

If an Investigator becomes aware of an SAE after the end of the trial, and he/she assesses the SAE to be related to the IMP, the case will have to be reported to Ferring according to the procedure specified in Section 8.4.2; regardless how long after the end of the trial this takes place. Such reports will be considered for expedited reporting and managed as such by Ferring.

8.4.2 Collection, Recording and Reporting of Serious Adverse Events

SAE Reporting by the Investigator

When a SAE has occurred, all related information should be fully and promptly documented as detailed as possible by the investigator. The investigator should submit the SAE report forms to CFDA and Ferring within 24 hours of his/her awareness of SAE occurrence.

The required SAE report forms which investigators should submit are listed as following:

- Report to authority (CFDA) (Fax to 010 88363228)
    - within 24 hours (Day 1) of the investigators awareness (Day 0) by applying SAE report form released by CFDA in Chinese language
- Report to Ferring (*i.e.,* to local safety officer (LSO) of Ferring International Pharma-Science Centre(China) Co. Ltd) (email: SafetyMailboxChinaBeijing_BDC@ferring.com and/or Fax to 010 85295335)

- within 24 hours (Day 1) of the investigators awareness (Day 0) by applying SAE report forms released by CFDA in Chinese language
- within 24 hours (Day 1) of the investigators awareness (Day 0) by applying SAE report forms released by Ferring in English language

All reported SAE forms will be analysed and a Council of International Organization of Medical Sciences (CIOMS) forms will be sent to the investigator. The investigator should submit the SAE with this CIOMS form to Ethic Committee within 15 days.

Both CFDA SAE reporting form and Ferring Approved SAE Report Form should include the following information regarding the reported case of SAE: its severity, its relationship to the study drug, type of procedures carried out to the study drug and its current status.

Expedited Reporting by Ferring

Ferring will report all adverse events that are **serious, unexpected and with a reasonable possible causality to the IMP** as judged by either the investigator or Ferring to the relevant parties within the stipulated timelines.

Additionally all SAEs occurring in China, regardless of causality, will be processed by Ferring for reporting to the Chinese Regulators **as soon as possible**.

SAEs will be considered reportable regardless of whether or not the IMP was used in accordance with the provisions in the protocol, Investigator's Brochure and Company Core-Data Sheet (CCDS) of Pentasa (effective date 03 January 2014).

8.5 Follow-up of Adverse Events and Serious Adverse Events

8.5.1 Follow-up of Adverse Events with Onset during the Trial

During the trial, the investigator must follow-up on each adverse event until it is resolved or until the medical condition of the subject is stable.

After the subject's last visit, the investigator must follow-up on any adverse event classified as serious or considered to have a reasonable possible causality to the IMP until it is resolved or until the medical condition of the subject is stable. All such relevant follow-up information must be reported to Ferring. If the event is a chronic condition, the investigator and Ferring may agree that further follow-up is not required.

8.5.2 Collection of Serious Adverse Events with Onset after Last Trial

If an investigator becomes aware of an SAE after the subject's last visit, and he/she assesses the SAE to have a reasonable possible causality to the IMP, the case will have to be reported to Ferring, regardless how long after the end of the trial this takes place.

9 STATISTICAL METHODS

The Global Biometrics department of Ferring Pharmaceuticals A/S will be responsible for the statistical analyses. All analyses will be detailed in a separate Statistical Analysis Plan available before first subject first visit.

9.1 Determination of Sample Size

No formal sample size calculations have been performed for this trial. However, based on CFDA guidance on clinical pharmacokinetic study [16], 8 subjects who complete the study and are eligible for inclusion in the PP analysis set are considered sufficient to provide adequate information on the PK of PENTASA® enema.

Assuming a combined 20% rate of major protocol deviations (e.g. non-compliance of IMP retainment) and adverse events (AEs) leading to withdrawal, 10 eligible subjects will be recruited. 8 subjects will complete the study and are eligible for inclusion in the primary (PP analysis set) PK analysis population. Withdrawn subjects will be replaced to achieve at least 8 completers, in which at least 2 completers will be of the same gender.

9.2 Subject Disposition

All subjects who are screened and pass inclusion/exclusion criteria check on Day -1 will be accounted for. All post- inclusion/exclusion criteria check withdrawals and discontinuations will be summarised by time of and main reason for withdrawal or discontinuation.

If subjects are found not to be eligible and therefore are withdrawn from the trial prior to first IMP dosing, they are regarded screening failures. The number of screening failures will be reported in the clinical trial report. Screening failures will not otherwise be accounted for.

9.3 Protocol Deviations

Major protocol violations, such as significant non-compliance or other serious unforeseen violations deemed to invalidate the data and affect the conclusions of the trial will lead to exclusion of data from the PP analysis set.

Data will not be excluded from the data analysis in case of minor protocol violations.

The rating of protocol violations in ‘minor’ and ‘major’ will be agreed between the Investigator and the Sponsor on the basis of a review of data before the declaration of a clean file and lock of the database.

9.4 Analysis Sets

9.4.1 Intention-to-Treat (ITT) Analysis Dataset

The ITT analysis set will comprise data from all subjects found eligible on Day 1. The ITT dataset will be used for presentation of subject disposition.

9.4.2 Full Analysis Set (FAS)

The FAS will comprise data from all dosed subjects. The FAS will be used for presentation of compliance and all Baseline characteristics (demographics, medical history, prior and concomitant medication, and physical examination).

9.4.3 Per Protocol (PP) Dataset

The PP dataset will comprise data from all dosed subjects, except those excluded as a result of major protocol violations as defined in Section 9.3. The PP analysis set will be used for presentation of PK endpoints.

9.4.4 Safety Dataset

The safety analysis set comprises all treated subjects and is analysed according to the actual treatment received.

9.5 Trial Population

9.5.1 Demographics and other Baseline Characteristics

All Baseline information will be presented in data listings. Selected Baseline data will be tabulated by treatment group using the FAS.

9.5.2 Medical History, Concomitant Medication and Other Safety Evaluations

All data will be presented using descriptive statistics for the FAS.

Medical history will be coded using Medical Dictionary of Regulatory Activities (MedDRA), Version 17.0 or higher. Medical history will be summarised.

Prior and concomitant medication will be coded using the World Health Organisation dictionary (WHO Drug; version effective at the start of the trial) using the international non-proprietary name (INN; also called ‘generic name’) and therapeutic use (Anatomical Therapeutic Code; ATC), and summarised by ATC classification 1st level (alphabetically), ATC classification 2nd level (in decreasing order of frequency) and treatment arm/dose level.

9.5.3 Treatment compliance

Time of dose, number of administrations of IMP, and increased diaper weight will be listed per subject.

9.6 Pharmacokinetic Endpoint Assessments

The primary and secondary endpoints relate to the PK of the IMP. The analysis, statistical analyses, and presentation of these data are described below.

9.6.1 General Considerations

For each dose and study part, all individual concentration data and PK parameters will be listed and tabulated by treatment group using the PP data set. Missing values will not be imputed.

All estimated PK parameters will be presented in summary tables, graphs and listings.

9.6.2 Primary Pharmacokinetic Endpoints

The PK analysis will be performed by Department of Experimental Medicine, Ferring Pharmaceuticals A/S. The PK parameters will be calculated by non-compartmental analysis (NCA) using the software Phoenix® (Pharsight Corporation, USA). Actual sampling time points relative to dosing will be used for the NCA and on the individual plots of plasma concentration versus time. Plasma concentration values below LLQ will be presented as 0 and included in NCA if the corresponding sampling time is before t_{max}. Values below LLQ will be presented as “Not Detectable, ND” and excluded from NCA if the corresponding sampling time is after t_{max}. Missing values (e.g. no blood sample collected or no value obtained at analysis) will be excluded from the NCA. No formal analysis of “outliers” is planned.

PK parameters will be estimated based on measurements from Day 1 to Day 2 in the single dose part, from Day 5-6 and from Day 7-8 in the multiple dose part.

Selection of data points for calculation of $t_{½}$ via λ_z will be based on the following considerations:

- AUC will be calculated by the linear trapezoidal method;
- The automatic range selection used in Phoenix® will be used to propose an optimal number of time points to use for the calculation of λ_z;
- At least three samples above LLQ obtained during the log-linear elimination phase will be included in the calculation of the λ_z;
- The final selection of samples for calculation of λ_z will be based on visual inspection of log concentration-time plots of individual profiles.

From the plasma concentration-time data of mesalazine, the following single-dose and multiple-dose PK parameters will be estimated, if possible: AUC, AUC_t, AUC_{tau}, C_{max}, C_{avg}, t_{max}, CL/F, V_z/F, $t_{½}$ and λ_z of mesalazine. Summary statistics will be provided for the PK parameters.

Possible drug accumulation after multiple dose will be evaluated from AUC (AUC_t if AUC is not calculated) after 1st dose and AUC_{tau} after the 7th multiple dose.

9.6.3 Secondary Pharmacokinetic Endpoints

For urine concentration of mesalazine and N-acetyl-mesalazine and volume after the 7th dose, Ae will be estimated if possible. Summary statistics will be provided for the Ae.

9.7 Treatment Compliance

The number of doses administered and the number of subjects available to be dosed in treatment period (Day 1 to 8) will be presented by treatment for the FAS and PP analysis sets.

9.8 Safety

9.8.1 General Considerations

Safety parameters will be evaluated for the safety analysis data set.

AEs will be coded according to MedDRA, Version 17.0 or higher. All data will be listed by subject.

AEs will be classified as either pre-treatment emergent or treatment emergent (see Section 8.1 for definitions). Only treatment emergent AEs will be presented in summary tables. A separate data listing will be provided for AEs that are defined as pre-treatment.

Missing values will be treated as missing, except for causality, intensity, seriousness, and outcome of AEs. A “worst case” approach will be used: if causality is missing, the AE will be regarded as related to the IMP; if the intensity of an AE is missing, the AE will be regarded as severe; if seriousness is missing the AE will be regarded as serious; if outcome is missing and no date of outcome is present the outcome is regarded as ‘not yet recovered’.

9.8.2 Adverse Events

Pre-treatment adverse events

Events occurring between the subject signing the informed consent form and receiving the first dose of IMP on Day 1 will be listed by subject.

Overview of treatment emergent adverse events

A treatment emergent AEs summary table will be presented including for each treatment the number and percentage of subjects reporting at least one AE and the total number of events reported for each of the following categories:

- All AEs;
- Severe AEs;
- SAEs;
- Adverse drug reactions (ADRs)

AEs with the causal relationship to IMP judged as 'no reasonable possibility' are categorised as unrelated to the IMP. AEs with the causal relationship to IMP judged as 'a reasonable possibility' will be categorised as related to IMP and considered to be ADRs;

- AEs leading to withdrawal;
- Deaths.

Incidence of treatment emergent adverse events

Summary tables will be prepared for the incidence of treatment emergent AEs per treatment, by MedDRA system organ class (SOC) and preferred term. The number and percentage (%) of subjects reporting an AE, and the number of events reported will be presented. Summary tables will be prepared for:

- All treatment emergent AEs;
- AEs by causality;
- AEs by intensity;
- ADRs by intensity.

Serious adverse events, deaths, and other significant adverse events

Separate listings will be provided for SAEs, deaths, and other significant AEs, if any.

9.8.3 Safety Laboratory Variables

Clinical chemistry, haematology, and urinalysis parameters will be listed per subject by time and treatment. Values outside the reference range will be flagged.

In addition, safety laboratory parameters will be summarised by time and treatment. Shift tables will be presented for changes from Baseline (i.e. the last assessment prior to IMP administration) to the Day 2 visit (single-dose part) or Day 9 visit (multiple-dose part). Shift tables will present the total number of subjects, number of subjects having a shift, and percentage of subjects having a shift using a categorisation of low, normal, and high values at each visit. Low, normal, and high will be defined according to the reference ranges provided by the Local Laboratory.

- Low: Values which are below the lower reference range limit.
- Normal: Values which are within the reference range.
- High: Values which are above the upper reference range limit.

For categorical urinalysis parameters, shift tables will summarise the number and percentage of subjects with 'absent' values at Baseline and 'present' values during the treatment period.

- Absent: No value for measured value.
- Present: Any value obtained for measured variable.

Similar shift tables will be prepared using the categories 'normal', 'abnormal not clinically significant', and 'abnormal clinically significant' as judged by the Investigator.

9.8.4 Vital Signs

Vital signs (blood pressure, heart rate, axillary temperature and respiratory rate) will be presented by time for each parameter and summarised by treatment. Shift tables will be presented for changes from Baseline (i.e., the last assessment prior to IMP administration) to End-of-trial examination, summarised by study part.

9.8.5 ECG

All 12- lead ECG data will be listed for each subject and summarised by time and treatment part. In addition, shift tables will be prepared using categorizations of 'normal', 'abnormal, not clinically significant', or 'abnormal, clinically significant' as judged by the Investigator.

9.8.6 Physical Examination

Physical examination findings at Screening and End-of-trial examination will be listed by subject.

9.9 Interim Analyses

No interim analysis is planned.

10 DATA HANDLING

10.1 Source Data and Source Documents

Source Data – ICH Definition

Source data are defined as all information in original records and certified copies of original records of clinical findings, observations, or other activities in a clinical trial necessary for the reconstruction and evaluation of the trial. Source data are contained in source documents (original records or certified copies).

Source Documents - ICH Definition

Source documents are defined as original documents, data, and records (e.g. hospital records, clinical and office charts, laboratory notes, memoranda, pharmacy dispensing records, recorded data from automated instruments, copies or transcriptions certified after verification as being accurate copies, microfiches, photographic negatives, microfilm or magnetic media, x-rays, subject files, and records kept at the pharmacy, at the laboratories and at medico-technical departments involved in the clinical trial).

Trial-specific Source Data Requirements – Ferring

The investigator must maintain subject records.

Subject's medical history, results of the physical examination and other clinically relevant findings, demographic data, and AEs will be documented on special source data sheets and then transferred to the individual CRF. Laboratory parameters will be printed out from a database and pasted on the local document. The laboratory results will be transposed into CRF by the Investigator. The results of 12-lead ECG assessments will be transcribed to special sections on the CRF, and annotated as normal, abnormal not clinically significant, or abnormal clinically significant. Where necessary, a detailed description of the exact abnormality will also be documented in the CRF.

10.2 CRF

For each subject enrolled, a CRF will be completed and signed by the Investigator. The completed original CRFs are the sole property of the Sponsor and should not be made available in any form to third parties, except the authorized representatives of appropriate regulatory authorities or the EC, without written permission from the Sponsor (see Section 14).

It is the Investigator's responsibility to ensure completion and to review and approve all CRFs. All CRFs must be filled out legibly in black or blue ink. Corrections are to be made by lining out the incorrect information and writing in revisions. All corrections must be approved and dated. These signatures serve to attest that the information contained on the CRFs is true. At all times, the Investigator has final responsibility for the accuracy and authenticity of all clinical and laboratory

data entered on the CRFs. Subject source documents are the physician's subject records maintained at the study site. Trial data must be entered into the CRF in a timely manner.

10.3 Data Management

Data management will be outsourced to a qualified Contract Research Organization (CRO). A data management plan will be created under the responsibility of the CRO. The data management plan will be issued before data collection begins and will describe all functions, processes, and specifications for data collection, cleaning and validation.

Data entry will be performed in accordance with the standard operating procedures of the outsourced CRO. The system used for data capture is fully validated. For medical coding of AEs, medical history and concomitant medication MedDRA (version 17.0 or higher) and WHO-Drug will be used. When all data have been processed, queries resolved, medical coding completed and any issues from review of protocol violations and data listings resolved, the database will be locked and all update access removed. The final database will be structured according to Ferring's data specifications.

10.4 Provision of Additional Information

On request, the Investigator will provide the Sponsor with additional data relating to the trial, or copies of relevant source records, duly anonymized. In case of particular issues or governmental queries, it may be necessary to have access to the complete trial documents, provided that the subjects' confidentiality is protected in accordance with applicable requirements.

11 MONITORING PROCEDURES

11.1 Periodic Monitoring

The monitor will contact and visit the investigator periodically to ensure adherence to the protocol, International Conference of Harmonisation-Good Clinical Practice (ICH-GCP), standard operating procedures and applicable regulatory requirements, maintenance of trial-related source records, completeness, accuracy and verifiability of CRF entries compared to source data, verification of drug accountability and compliance to safety reporting instructions.

The investigator will permit the monitor direct access to all source data, including electronic medical records, and/or documents in order to facilitate data verification. The investigator will co-operate with the monitor to ensure that any discrepancies that may be identified are resolved. The investigator is expected to be able to meet the monitor during these visits. When the first subject is allocated to treatment at the trial site a monitoring visit will take place shortly afterwards.

100% source data verification (SDV) will be performed. The process of SDV will be described in detail in the Monitoring manual for the trial.

11.2 Audit and Inspection

The investigator will make all the trial-related source data and records available to quality assurance auditors mandated by Ferring, or to domestic/foreign regulatory inspectors or representatives from EC who may audit/inspect the trial after giving reasonable notice.

The main purposes of an audit or inspection are to confirm that all data relevant for the evaluation of the investigational product have been processed and reported in compliance with GCP and applicable regulatory requirements.

The subjects must be informed by the investigator and in the Informed Consent Documents that authorised Ferring representatives and representatives from regulatory authorities and EC may wish to inspect their medical records. During audits/inspections the auditors/inspectors may copy relevant parts of the medical records. No personal identification apart from the screening/randomisation number will appear on these copies.

The investigator should notify Ferring without any delay of any inspection by a regulatory authority or EC.

11.3 Confidentiality of Subject Data

The investigator will ensure that the confidentiality of the subjects' data will be preserved. On CRFs or any other documents submitted to Ferring, the subjects will not be identified by their names, but by an identification system, which consists of an assigned number in the trial. Documents that are not for submission to Ferring, e.g. the confidential subject identification code

and the signed Informed Consent Documents, will be maintained by the investigator in strict confidence.

12 CHANGES IN THE CONDUCT OF THE TRIAL

12.1 Protocol Amendments

Any change to this protocol will be documented in a protocol amendment agreed by the Investigator and the Sponsor prior to its implementation. Any amendments will be submitted to the EC.

An EC approval is required for any amendment which could affect the safety of the subjects, or which entails a change of the scope/design of the trial, such as an increase in dose or duration of exposure to the IMP, an increase in the number of subjects, the addition of a new test or procedure, or the dropping of a test intended to monitor safety.

However, changes to the protocol to eliminate immediate hazard(s) to trial subjects may be implemented prior to EC approval.

Protocol amendments will be submitted for notification of ECs and the CFDA, in accordance with local regulations.

12.2 Deviations from the Protocol

If deviations from the protocol occur, the investigator must inform the monitor, and the implications of the deviation must be reviewed and discussed. Any deviation must be documented, either as answer to a query from the CRF, in a protocol deviation report or a combination of both. A log of protocol deviation reports will be maintained by Ferring. Protocol deviation reports and supporting documentation must be kept in the Investigator's File and the Trial Master File.

12.3 Premature Trial Termination

Both the investigator (with regard to his/her participation) and Ferring reserve the right to terminate the trial at any time. Should this become necessary, the procedures will be agreed upon after consultation between the two parties. In terminating the trial, Ferring and the investigator will ensure that adequate consideration is given to the protection of the best interests of the subjects. Regulatory authorities and ECs will be informed.

In addition, Ferring reserves the right to terminate the participation of individual trial sites. Conditions that may warrant termination include, but are not limited to, insufficient adherence to protocol requirements and failure to enter subjects at an acceptable rate.

13 REPORTING AND PUBLICATION

13.1 Clinical Trial Report

The data and information collected during this trial will be reported in a clinical trial report prepared by Ferring and submitted for comments and signature to the signatory investigator(s).

13.2 Confidentiality and Ownership of Trial Data

Any confidential information relating to the IMP or the trial, including any data and results from the trial will be the exclusive property of Ferring. The investigator and any other persons involved in the trial will protect the confidentiality of this proprietary information belonging to Ferring.

13.3 Publications and Public Disclosure

13.3.1 Publication Policy

At the end of the trial, one or more manuscripts for joint publication may be prepared in collaboration between the investigator(s) offered authorship and Ferring.

Authorship is granted based on the ICMJE criteria (see current official version: http/www.ICMJE.org). The total number of authors is based on the guideline from the relevant journal or congress. In the event of any disagreement in the content of a publication, both the investigator’s and Ferring’s opinion will be fairly and sufficiently represented in the publication.

Any external CRO or laboratory involved in the conduct of this trial has no publication rights regarding this trial.

If the investigator wishes to independently publish/present any results from the trial, the draft manuscript/presentation must be submitted in writing to Ferring for comment prior to submission. Comments will be given within four weeks from receipt of the draft manuscript. This statement does not give Ferring any editorial rights over the content of a publication, other than to restrict the disclosure of Ferring’s intellectual property. If the matter considered for publication is deemed patentable by Ferring, scientific publication will not be allowed until after a filed patent application is published. Under such conditions the publication will be modified or delayed at the investigator’s discretion, to allow sufficient time for Ferring to seek patent protection of the invention.

13.3.2 Public Disclosure Policy

ICMJE member journals have adopted a trials-registration policy as a condition for publication. This policy requires that all clinical trials be registered in a public, clinical trials registry. Thus, it is the responsibility of Ferring to register the trial in an appropriate public registry, i.e. http://www.chinadrugtrials.org.cn/ which is designated by CFDA.

14 ETHICAL AND REGULATORY ASPECTS

14.1 Ethics Committee (EC)

An EC will review the protocol and any amendments and advertisements used for recruitment. The IEC/IRB will review the Subject Information Sheet and the Informed Consent Form, their updates (if any), and any written materials given to the subjects.

14.2 Regulatory Authority(ies) Authorisation / Approval / Notification

Regulatory permission from CFDA to perform the trial will be obtained in accordance with relevant provision in China's law (2012L01750). All ethical and regulatory approvals must be available before a subject is exposed to any trial-related procedure, including screening tests for inclusion/exclusion criteria check.

14.3 End-of-Trial and End-of-Trial Notification

End-of-trial is defined as the date the last subject performs the last visit in the trial. End-of-trial notification will be reported according to local regulations.

14.4 Ethical Conduct of the Trial

This trial will be conducted in accordance with the ethical principles that have their origins in the Declaration of Helsinki, in compliance with the approved protocol, GCP and applicable regulatory requirements.

14.5 Subject Information and Consent

The investigator (or the person delegated by the investigator) will obtain a freely given written consent from each subject after an appropriate explanation of the aims, methods, anticipated benefits, potential hazards, required cooperation, insurance, confidentiality, and any other aspects of the trial which are relevant to the subject's decision to participate. The trial subject must be given ample time to consider participation in the trial, before the consent is obtained. The Informed Consent Documents must be signed and dated by the subject and the investigator who has provided information to the subject regarding the trial before the subject is exposed to any trial-related procedure, including screening tests for inclusion/exclusion criteria check.

The investigator (or the person delegated by the investigator) will explain that the subject is completely free to refuse to enter the trial or to withdraw from it at any time, without any consequences for his/her further care and without the need to justify his/her decision.

The subject will receive a copy of the Subject Information and his/her signed Informed Consent Form.

If new information becomes available that may be relevant to the trial subject's willingness to continue participation in the trial, a new Subject Information and Informed Consent Form will be

forwarded to the EC. The trial subjects will be informed about this new information and re-consent will be obtained.

Each subject will be informed that the monitor(s), quality assurance auditor(s) mandated by Ferring, EC representatives or regulatory authority inspector(s), in accordance with applicable regulatory requirements, may review his/her source records and data. Data protection will be handled in compliance with national/local regulations.

14.6 Subject Information Card

Subject Information Card will not be provided, since subjects will have a residential stay at hospital from the day of first IMP dosing until End-of-trial.

14.7 Compliance Reference Documents

The Helsinki Declaration, the consolidated ICH-GCP, and other national law(s) in China shall constitute the main reference guidelines for ethical and regulatory conduct.

15 LIABILITIES AND INSURANCE

15.1 ICH-GCP Responsibilities

The responsibilities of Ferring, the monitor and the investigator are defined in the ICH-GCP consolidated guideline, and applicable regulatory requirements in the country where the trial takes place. The investigator is responsible for adhering to the ICH-GCP responsibilities of investigators, for dispensing the IMP in accordance with the approved protocol or an approved amendment, and for its secure storage and safe handling throughout the trial.

15.2 Liabilities and Insurance

In case of any damage or injury occurring to a subject in association with the IMP or the participation in the trial, Ferring has contracted an insurance which covers the liability of Ferring, the investigator and other persons involved in the trial in compliance with the laws in the countries involved.

16 ARCHIVING

16.1 Investigator File

The investigator is responsible for maintaining all the records, which enable the conduct of the trial at the site to be fully understood, in compliance with ICH-GCP. The trial documentation including all the relevant correspondence should be kept by the investigator for at least 15 years after the completion or discontinuation of the trial, if no further instructions are given by Ferring.

The investigator is responsible for the completion and maintenance of the confidential subject identification code which provides the sole link between named subject source records and anonymous CRF data for Ferring. The investigator must arrange for the retention of this Subject Identification Log and signed Informed Consent Documents for at least 15 years after the completion or discontinuation of the trial.

No trial site document may be destroyed without prior written agreement between the investigator and Ferring. Should the investigator elect to assign the trial documents to another party, or move them to another location, Ferring must be notified. If the investigator retires and the documents can no longer be archived by the site, Ferring can arrange having the Investigator File archived at an external archive.

16.2 Trial Master File

Ferring will archive the Trial Master File in accordance with ICH-GCP and applicable regulatory requirements.

17 REFERENCES

1. Jang ES, Lee DH, Kim J, et al. Age as a clinical predictor of relapse after induction therapy in ulcerative colitis. Hepatogastroenterology. 2009;56:1304-9.
2. Jie Zhao, Siew C. Ng, Yuan Lei, et al. First Prospective, Population-Based Inflammatory Bowel Disease Incidence Study in Mainland of China: The Emergence of “Western” Disease. Inflamm Bowel Dis. 2013; 9: 1839-45.
3. Qin OY, et al. Experts’consensus on the diagnosis and management of inflammatory bowel disease. Chin J Intern Med 2008; 47:73-9.
4. Baumgart DC and Sandborn WJ. Inflammatory bowel disease: clinical aspects and established and evolving therapies. Lancet. 2007; 369:1641-57.
5. Carter MJ, Lobo AJ, et al. Guidelines for the management of inflammatory bowel disease in adults. Gut. 2004; 53:V1-16.
6. Marteau P, Seksik P, et al. Recommendations for clinical practice for the treatment of ulcerative colitis. Gastroenterol Clin Biol. 2004; 28:955-60.
7. Jacobsen, BA, et al. Availability of mesalazine (5-aminosalicylic acid) from enemas and suppositories during steady-state conditions. Scand J Gastroenterol. 1991. 26: p. 374-8.
8. Ahnfelt-Ronne, I. and O.H. Nielsen, The antiinflammatory moiety of sulfasalazine, 5-aminosalicylic acid, is a radical scavenger. Agents Actions, 1987. 21(1-2): p. 191-4.
9. Fujiwara, M., K. Mitsui, and I. Yamamoto, Inhibition of proliferative responses and interleukin 2 productions by salazosulfapyridine and its metabolites. Jpn J Pharmacol, 1990. 54(2): p. 121-31.
10. Gionchetti, P., et al., Scavenger effect of sulfasalazine, 5-aminosalicylic acid, and olsalazine on superoxide radical generation. Dig Dis Sci, 1991. 36(2): p. 174-8.
11. Hoult, J.R. and P.K. Moore, Effects of sulphasalazine and its metabolites on prostaglandin synthesis, inactivation and actions on smooth muscle. Br J Pharmacol, 1980. 68(4): p. 719-30.
12. Wilding IR HG, Brown J, Sparrow RA, Kenyon CJ. Scintigraphic evaluation of four rectally administered PENTASA® formulations. Research report. Study code: PPL-104., Vanløse, Denmark: Ferring A/S; 1996.
13. Rambaud JC MP. A comparative study of systemic absorption of mesalazine enema and foam (1g/application). Clinical study report, Gentilly, France: Ferring S/A; 1996. Protocol no. PENTAMOUKIN/93/02.
14. Bondesen S., et al. Pharmacokinetics of 5-aminosalicylic acid in man following administration of intravenous bolus and per os slow-release formulation. Dig Dis Sci. 1991. 36: 1735-40.
15. Myers, B., et al. Metabolism and urinary excretion of 5-amino salicylic acid in healthy volunteers when given intravenously or released for absorption at different sites in the gastrointestinal tract. Gut. 1987. 28: p. 196-200.
16. CFDA. Guidance on clinical pharmacokinetics study of chemical drugs.2005.

APPENDICES

Appendix 1 Introduction to IMP Administration

Note*: The instruction was adapted from the drug label and demonstrates patient-self administration. IMP will be administered by trial nurse in this PK study. The IMP should be pre-heated to 30~37°C in water bath, with the bottle in aluminium foil pack.*

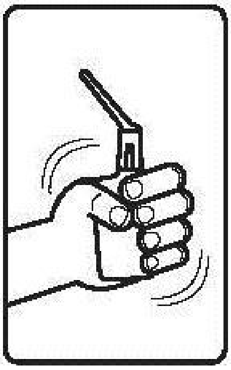

1. Immediately before use take the enema bottle out of the aluminium foil pack and shake it well.

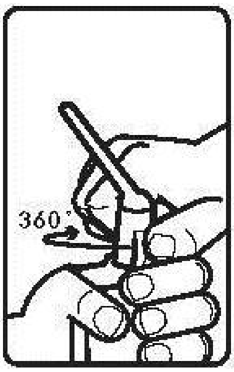

2. To break the seal twist the nozzle clockwise one full turn (the nozzle should then be in the same direction as before turning).

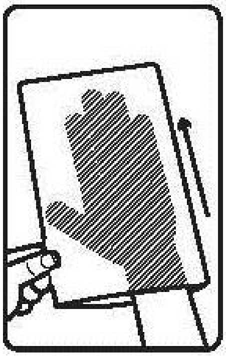

3. Put your hand in one of the plastic disposal bags provided in the pack.

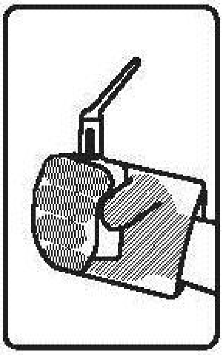

4. Hold the container as shown in the picture.

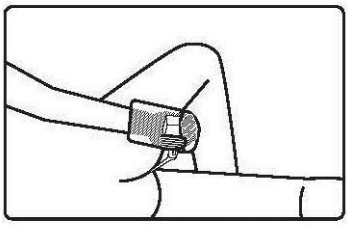

5. To administer the enema, lie on your left side with the left leg straight and the right leg bent forward for balance. Carefully insert the applicator tip into the rectum. Maintain sufficient steady hand pressure while dispersing the bottle content. The bottle content should be applied within max. 30-40 seconds.

6. Once the bottle is empty, withdraw the tip with the bottle still compressed.

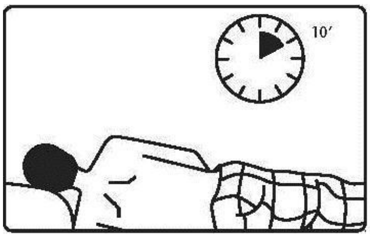

7. The enema should be retained in the bowel. Remain relaxed in the administration position for 5-10 minutes or until the urge to pass the enema has disappeared.

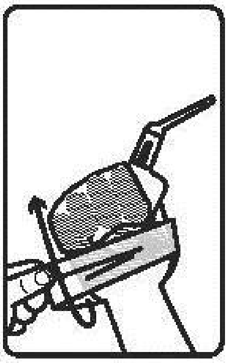

8. Roll the plastic disposal bag over the empty bottle. Discard it.

Appendix 2 Introduction to Bowel Cleaning Using Water Enema

1. Use enemator which are approved in China as applicator.
2. Warm 1000 mL purified water to approximately 30~37°C.
3. Subjects will lie on their left side.
4. Insert vaseline-lubricated cannula of enemator into rectum, about 10~15 cm in depth. The cannula is connected to a bag containing 1000 mL water which is about 40~60 above the anus. (If the capacity of the bag is less than 1000 mL, water could be poured into the bag in several times, to achieve the total volume of 1000 mL.)
5. Introduce 500~1000 mL water into rectum by rectal cannula.
6. Subjects will keep lying in bed on their left side for 5-10 minutes after extracting cannula, and then go to toilet and evacuate their bowels.
7. Step 4-6 could be repeated once at the Investigator's discretion, e.g., less than 500 mL water is induced at first time. Dosing of water enema will not be performed more than 2 times. The total volume of introduced water is n 1000 mL for a single subject.
